# Supplementary material for: Clinically SUspected ScaPhoid fracturE: treatment with supportive bandage or CasT? ‘Study protocol of a multicenter randomized controlled trial’ (SUSPECT study)
Source: BMJ Open. 2020 Sep 29;10(9):e036998. doi: 10.1136/bmjopen-2020-036998 (PMC7526317; doi:10.1136/bmjopen-2020-036998)
Supplement: Supplementary data [file bmjopen-2020-036998supp001.pdf]

Proefpersoneninformatie voor deelname aan medisch-wetenschappelijk onderzoek

Titel van het onderzoek

Polspijn zonder afwijkingen op de röntgenfoto's

Geachte heer/mevrouw,

U heeft de spoedeisende hulp bezocht vanwege pijn in uw pols. Wij willen u vragen of u wilt deelnemen aan een medisch-wetenschappelijk onderzoek. U beslist zelf of u wilt meedoen. Voordat u de beslissing neemt, is het belangrijk om meer te weten over het onderzoek. Lees daarom deze informatiebrief rustig door. Bespreek het met partner, vrienden of familie. Ook is er een onafhankelijke persoon, die veel weet van het onderzoek. Lees ook de Algemene brochure. Daar staat veel algemene informatie over medisch-wetenschappelijk onderzoek in. Heeft u na het lezen van de informatie nog vragen? Dan kunt u terecht bij de arts-onderzoeker. In bijlage C vindt u haar contactgegevens.

1. Algemene informatie

Dit onderzoek is opgezet door het Erasmus MC en het Reinier de Graaf Gasthuis en wordt gedaan door artsen in verschillende ziekenhuizen.
Voor dit onderzoek zijn 180 proefpersonen nodig die polspijn hebben na een ongeval.
De medisch-ethische toetsingscommissie van het Erasmus MC heeft dit onderzoek goedgekeurd.
Algemene informatie over de toetsing van onderzoek vindt u in de brochure 'Medisch-wetenschappelijk onderzoek'. Deze brochure is ook online beschikbaar. Zie hiervoor de volgende link: file:///H:/Downloads/medisch-wetenschappelijk+onderzoek+algemene+informatie+voor+de+proefpersoon.pdf

2. Wat is het doel van het onderzoek?

Polspijn na een ongeval kan veroorzaakt worden door een kneuzing of een botbreuk. Om het onderscheid te kunnen maken tussen een kneuzing of een botbreuk zijn er bij u een aantal röntgenfoto's gemaakt. Er zijn op dit moment op uw röntgenfoto's geen botbreuken te zien zijn, maar een breuk van het scheepvormige botje kan soms pas te zien zijn na 2 weken. Daarom worden alle patiënten met verdenking op een botbreuk van het scheepvormige botje behandeld met onderarmgips en na 2 weken teruggezien. Aangezien uiteindelijk maar 1 van de 10 patiënten een

botbreuk heeft worden dus veel patiënten onnodig behandeld met gips. Met dit onderzoek willen we daarom kijken of de behandeling niet even goed is met een drukverband gedurende 3 dagen in plaats van een onderarmgips voor 2 weken.

3. Wat wordt er onderzocht?

In dit onderzoek wordt een drukverband vergeleken met een onderarm gips. Om dit te kunnen beoordelen willen wij u vragen om gedurende de studie een aantal vragenlijsten in te vullen. Voor het onderzoek is het belangrijk dat u dit zo objectief mogelijk invult. Daarnaast zullen wij de functie van de pols beoordelen.

4. Wat meedoen inhoudt?

Als u meedoet, duurt dat totaal ongeveer 1 jaar voor u. De studie gaat in totaal vier jaar duren.

Behandeling

Als u besluit om mee te doen zal er worden geloot tussen de behandeling met onderarmgips gedurende 2 weken en de behandeling met drukverband gedurende 3 dagen. Uw behandelend arts en de onderzoekers hebben geen invloed op de uitslag van de loting.

Bezoek en metingen

Gedurende de studie zullen wij op vijf momenten vragen om vragenlijsten in te vullen (ongeveer 15 minuten per meetmoment). Via deze vragenlijsten zullen wij het dagelijks functioneren van de arm, kwaliteit van leven en de kosten met betrekking tot medische zorg en arbeidsverzuim evalueren. Daarnaast zullen wij tijdens twee bezoeken aan het ziekenhuis de onderarmen onderzoeken op bewegelijkheid en kracht. Ook zullen er röntgenfoto's worden gemaakt om de botten van de pols goed in beeld te brengen. Op het moment dat er tijdens deze controles een breuk wordt ontdekt op de röntgenfoto's zal de behandeling zich richten op herstel van deze breuk. In bijlage B vindt u een overzicht van alles wat wordt gepland als u deelneemt.

Anders dan gebruikelijke zorg

Als u aan het onderzoek deelneemt dan krijgt u afhankelijk van de loting een drukverband. Dit is momenteel niet de standaardbehandeling. Indien u inloot voor de groep met het onderarmgips, dan krijgt u gewoon de behandeling die u ook zou krijgen als u niet meedeed aan dit onderzoek.
Na twee weken wordt er nogmaals een röntgenfoto gemaakt. Dit maakt onderdeel uit van de standaardbehandeling en zal dus ook gebeuren als u niet meedoet aan dit onderzoek. Mocht er dan toch een botbreuk op de röntgenfoto's te zien zijn, wordt u alsnog behandeld voor de botbreuk. Eerder onderzoek heeft aangetoond dat latere behandeling van een botbreuk van het scheepvormige botje geen nadelige gevolgen heeft voor het uiteindelijk herstel. Indien u deelneemt aan het onderzoek krijgt u een extra poliklinische controle na 1 jaar. Deze extra poliklinische controle

is voor beide groepen hetzelfde. Hier zullen voor u geen extra kosten aan verbonden zijn. De reguliere behandeling bestaat uit een onderarmgips en poliklinische controle met röntgenfoto’s twee weken na het ongeval (zie bijlage B).

5. Wat wordt er van u verwacht?

Om het onderzoek goed te laten verlopen is het belangrijk dat u zich aan de volgende afspraken houdt.

De afspraken zijn dat u:

- de gips of verband behandeling volgt volgens de uitleg.
- niet ook nog aan een ander medisch-wetenschappelijk onderzoek meedoet, die invloed heeft op de huidige studie.
- afspraken voor bezoeken nakomt.

Het is belangrijk dat u contact opneemt met de onderzoeker:

- als u in een ziekenhuis wordt opgenomen of behandeld.
- als u plotseling gezondheidsklachten krijgt.
- als u niet meer wilt meedoen aan het onderzoek.
- als uw contactgegevens wijzigen.

6. Welke bijwerkingen en risico’s kunt u verwachten?

De behandeling van polspijn zonder afwijkingen op de röntgenfoto’s brengt niet veel bijwerkingen of risico’s met zich mee. De behandeling met gips zal meer kans geven op drukplekken van het gips en stijfheid van de pols. De behandeling met drukverband geeft mogelijk een groter risico op trager herstel van een mogelijk botbreuk van het scheepvormig botje alhoewel eerdere studies dit ontkrachten.

7. Wat zijn mogelijke voor- en nadelen van deelname aan dit onderzoek?

U heeft zelf geen voordeel van deelname aan dit onderzoek. Voor de toekomst kan het onderzoek wel nuttige gegevens opleveren. Een nadeel van deelname aan de studie is dat wij extra tijd van u vragen voor het invullen van de vragenlijsten en de extra metingen.

8. Wat gebeurt er als u niet wenst deel te nemen aan dit onderzoek?

U beslist zelf of u meedoet aan het onderzoek. Deelname is vrijwillig. Als u besluit niet mee te doen, hoeft u verder niets te doen. U hoeft niets te tekenen. U hoeft ook niet te zeggen waarom u niet wilt meedoen. U krijgt natuurlijk alle noodzakelijke medische behandelingen. Als u wel meedoet, kunt u zich altijd bedenken en toch nog stoppen. Dat kan ook tijdens het onderzoek. Wel moet u dit direct

melden aan de onderzoeker. Wij vragen u dan wel of we enkele gegevens uit uw patiëntendossier mogen gebruiken voor het onderzoek.
De gegevens die tot dat moment zijn verzameld, worden gebruikt voor het onderzoek.
Als er nieuwe informatie over het onderzoek is die belangrijk voor u is, laat de onderzoeker dit aan u weten. U wordt dan gevraagd of u blijft meedoen.

9. Wat gebeurt er als het onderzoek is afgelopen?

Uw deelname aan het onderzoek stopt als

- alle bezoeken [volgens het schema/zoals beschreven onder punt 4] voorbij zijn
- u zelf kiest om te stoppen
- de onderzoeker het beter voor u vindt om te stoppen
- de subsidiegever van het onderzoek, de overheid of de beoordelende medisch-ethische toetsingscommissie, besluit om het onderzoek te stoppen.

Het hele onderzoek is afgelopen als alle deelnemers klaar zijn.
Na het verwerken van alle gegevens informeert de onderzoeker u over de belangrijkste uitkomsten van het onderzoek.

10. Gebruik en bewaren van uw gegevens

Voor dit onderzoek is het nodig dat uw medische gegevens worden verzameld en gebruikt. Elke proefpersoon krijgt een code die op de gegevens komt te staan. Uw naam en andere persoonlijke gegevens die u direct kunnen identificeren worden daarbij weggelaten

Uw gegevens

Al uw gegevens blijven vertrouwelijk.
Sommige mensen mogen uw medische en persoonlijke gegevens inzien. Dit is om te controleren of het onderzoek goed en betrouwbaar uitgevoerd is. Algemene informatie hierover vindt u in de brochure ‘Medisch-wetenschappelijk onderzoek’.
Mensen die uw gegevens kunnen inzien zijn: het onderzoeksteam, een controleur die voor de opdrachtgever van het onderzoek werkt, de Inspectie voor de Gezondheidszorg. Zij houden uw gegevens geheim. De onderzoeker van het coördinerend centrum (Erasmus MC) ontvangt uw gegevens voor het versturen van de vragenlijsten. Deze gegevens zullen na het einde van het onderzoek vernietigd worden.
Als u de toestemmingsverklaring ondertekent, geeft u toestemming voor het verzamelen, bewaren en inzien van uw medische en persoonlijke gegevens.
De onderzoeker bewaart uw gegevens 15 jaar.

Later gebruik gegevens

Wij willen uw gegevens graag bewaren. Misschien kunnen we daar later extra onderzoek mee doen. Op het toestemmingsformulier kunt u aangeven of u hiermee akkoord gaat. U kunt deze toestemming altijd weer intrekken.

Inzien gegevens en intrekken toestemming

U heeft te allen tijde het recht om de over u verzamelde gegevens in te zien, te wijzigen of uw toestemming voor gebruik van uw persoonsgegevens weer in te trekken. De onderzoeksgegevens die zijn verzameld tot het moment dat u uw toestemming intrekt worden nog wel gebruikt in het onderzoek.

Meer informatie over uw rechten bij verwerking van gegevens

Voor algemene informatie over uw rechten bij verwerking van uw persoonsgegevens kunt u de website van de Autoriteit Persoonsgegevens raadplegen (https://autoriteitpersoonsgegevens.nl).

Bij vragen over uw rechten kunt u contact opnemen met de verantwoordelijke voor de verwerking van uw persoonsgegevens. Voor dit onderzoek is dat:

Erasmus Medisch Centrum: Zie bijlage C voor contactgegevens.

Bij vragen of klachten over de verwerking van uw persoonsgegevens raden we u aan eerst contact op te nemen met de onderzoekslocatie. U kunt ook contact opnemen met de Functionaris voor de Gegevensbescherming van de instelling [contactgegevens in bijlage C] of de Autoriteit Persoonsgegevens.

11. Verzekering voor proefpersonen

Voor iedereen die meedoet aan dit onderzoek is een verzekering afgesloten. De verzekering dekt schade als gevolg van het onderzoek. Dit geldt voor schade die naar boven komt gedurende het onderzoek. In bijlage A vindt u de verzekerde bedragen, de uitzonderingen en de adresgegevens van de verzekeraar.

12. Informeren huisarts

Uw huisarts wordt geïnformeerd over uw deelname aan het onderzoek. Dit is voor uw eigen veiligheid. Als u dit niet goed vindt, kunt u niet meedoen aan dit onderzoek. U kunt niet deelnemen aan het onderzoek als u geen huisarts heeft.

13. Zijn er extra kosten/is er een vergoeding wanneer u besluit aan dit onderzoek mee te doen?

Er zullen geen extra kosten in rekening worden gebracht bij deelname aan het onderzoek. Het bezoek aan de spoedeisende hulp is standaard verzekerde zorg. Het extra poliklinische bezoek na 1

jaar en de daarbij behorende röntgenfoto's zullen betaald worden vanuit onderzoeksgeld en dus niet aan u worden doorberekend.

14. Wilt u verder nog iets weten?

Wilt u graag een onafhankelijk advies over meedoen aan dit onderzoek? Dan kunt u terecht bij een onafhankelijke arts. Zijn gegevens vindt u in *lokale informatie onafhankelijk arts* (bijlage C).
Indien u een klacht heeft, dan kunt u deze het beste eerst bespreken met degene die er rechtstreeks bij betrokken is. Kunt u uw klacht of ongenoegen, om welke reden dan ook, niet rechtstreeks bespreken of bent u na het bespreken van uw klacht niet tevreden, dan helpt de klachtenfunctionaris u graag verder. De gegevens van de klachtenfunctionaris vindt u in bijlage C.

15. Ondertekening toestemmingsformulier

Indien u toestemming geeft over deelname aan dit onderzoek, zullen wij u vragen deze op de bijbehorende toestemmingsverklaring schriftelijk te bevestigen. Door uw schriftelijke toestemming geeft u aan dat u de informatie heeft begrepen en instemt met deelname aan het onderzoek.
Het handtekeningenblad wordt door de onderzoeker bewaard. U krijgt een kopie of een tweede exemplaar van deze toestemmingsverklaring.

Dank voor uw aandacht.

Orthopedisch chirurgen Erasmus MC en het onderzoeksteam

Polspijn zonder afwijkingen op de röntgenfoto's

Bijlagen

A. Informatie over de verzekering
B. Overzicht metingen onderzoek
C. Lokale informatie
D. Toestemmingsverklaring

Cohen A, *et al. BMJ Open* 2020; 10:e036998. doi: 10.1136/bmjopen-2020-036998

Bijlage A: Informatie over de verzekering

Voor iedereen die meedoet aan dit onderzoek is een verzekering afgesloten. De verzekering dekt schade die het gevolg is van deelname aan het onderzoek. Dit geldt voor schade die naar boven komt tijdens het onderzoek, of binnen vier jaar na het einde van het onderzoek. U moet de schade ook binnen die 4 jaar aan de verzekeraar hebben gemeld.

In geval van schade kunt u zich direct wenden tot de verzekeraar.

De verzekeraar van het onderzoek is:

CAN Insurance Company Limited,

Mevrouw Esther van Herk

+31(0)205737274

Esther.vanherk@cnahardy.com

De verzekering biedt een maximum dekking van € 650.000 per proefpersoon, met een maximumbedrag van € 5.000.000 voor het gehele onderzoek. Indien de opdrachtgever van dit onderzoek meerdere onderzoeken heeft lopen, geldt een maximumbedrag van € 7.500.000 euro per verzekeringsjaar voor álle onderzoeken. De dekking van specifieke schades en kosten is verder tot bepaalde bedragen beperkt. Deze vindt u in het *Besluit verplichte verzekering bij medisch wetenschappelijk onderzoek met mensen*. Informatie hierover kunt u vinden op de website van de Centrale Commissie Mensgebonden Onderzoek: www.ccmo.nl.

Voor deze verzekering gelden voorts een aantal uitsluitingen. De verzekering dekt niet:

- schade waarvan op grond van de aard van het onderzoek (nagenoeg) zeker was dat deze zich zou voordoen;
- schade aan de gezondheid die ook zou zijn ontstaan als u niet aan het onderzoek had deelgenomen;
- schade die het gevolg is van het (niet) volledig nakomen van aanwijzingen of instructies;
- schade aan nakomelingen, als gevolg van een nadelige inwerking van het onderzoek op u of uw nakomeling;
- bij onderzoek naar bestaande behandelmethoden: schade die het gevolg is van één van deze behandelmethoden;
- bij onderzoek naar de behandeling van specifieke gezondheidsproblemen: schade die het gevolg is van uitblijvende verbetering of van verslechtering van deze gezondheidsproblemen.

Polspijn zonder afwijkingen op de röntgenfoto's

	**T1** Start studie	**T 2** 2 weken na start studie	**T3** 6 weken na start studie	**T4** 3 maanden na start studie	**T5** 6 maanden na start studie	**T6** 9 maanden na start studie	**T7**12 maanden na start studie
Vragenlijsten (thuis)	+	+	+	+	+	+	+
Telefonisch contact					+	+	
Lichamelijk onderzoek (polikliniek)	*	*					+
Röntgenfoto's (polikliniek)	*	*					+

Bijlage B: overzicht metingen onderzoek

+ = extra metingen voor onderzoek, wordt uitgevoerd / afgenomen bij deze controle
* = metingen die behoren bij gebruikelijke zorg, wordt uitgevoerd bij dit bezoek

Cohen A, *et al. BMJ Open* 2020; 10:e036998. doi: 10.1136/bmjopen-2020-036998

Polspijn zonder afwijkingen op de röntgenfoto's

Bijlage C. Lokale informatie Erasmus Medisch Centrum

Hoofdonderzoeker:
dr. J.W. Colaris 010-7034686
orthopedisch Chirurg
(via secretariaat Orthopedie)

Onafhankelijk arts:
dr. D.E. Meuffels, 010-7034686
orthopaedisch chirurg Erasmus MC
(via secretariaat Orthopaedie)

Arts-onderzoeker:
Abigaël Cohen 06-24788338
suspect@erasmusmc.nl

Klachtencommissie:
Klachtenfunctionarissen Erasmus MC 010-7033198

Functionaris voor de Gegevensbescherming van de instelling:
Voor meer informatie over uw rechten: 010-7034986
(via secretariaat afdeling Juridische Zaken)

Autoriteit Persoonsgegevens https://autoriteitpersoonsgegevens.nl

Bijlage D: Toestemmingsformulier proefpersoon

Titel van het onderzoek: Polspijn zonder afwijkingen op de röntgenfoto's

- Ik heb de informatiebrief voor de proefpersoon gelezen. Ik kon aanvullende vragen stellen. Mijn vragen zijn genoeg beantwoord. Ik had genoeg tijd om te beslissen of ik meedoe.
- Ik weet dat meedoen helemaal vrijwillig is. Ik weet dat ik op ieder moment kan beslissen om toch niet mee te doen. Daarvoor hoef ik geen reden te geven.
- Ik geef toestemming om mijn huisarts te vertellen dat ik meedoe aan dit onderzoek.
- Ik weet dat sommige mensen mijn gegevens kunnen zien. Die mensen staan vermeld in deze informatiebrief.
- Ik geef toestemming om mijn gegevens te gebruiken, voor de doelen die in de informatiebrief staan.
- Ik geef toestemming om mijn onderzoeksgegevens 15 jaar na afloop van dit onderzoek te bewaren.
- Ik geef toestemming om in de toekomst opnieuw benaderd te worden voor vervolg onderzoek. Indien ik dit niet wens, zal ik dat kenbaar maken.
- Ik wil meedoen aan dit onderzoek.

Naam proefpersoon: Handtekening:

Datum : __ / __ / __

Ik verklaar hierbij dat ik deze proefpersoon volledig heb geïnformeerd over het genoemde onderzoek.

Als er tijdens het onderzoek informatie bekend wordt die de toestemming van de proefpersoon zou kunnen beïnvloeden, dan breng ik hem/haar daarvan tijdig op de hoogte.

Naam onderzoeker (of diens vertegenwoordiger):

Handtekening: Datum: __ / __ / __

* Doorhalen wat niet van toepassing is.
