# Supplementary material for: Clinically SUspected ScaPhoid fracturE: treatment with supportive bandage or CasT? ‘Study protocol of a multicenter randomized controlled trial’ (SUSPECT study)
Source: BMJ Open. 2020 Sep 29;10(9):e036998. doi: 10.1136/bmjopen-2020-036998 (PMC7526317; doi:10.1136/bmjopen-2020-036998)
Supplement: Author's manuscript [file bmjopen-2020-036998.draft_revisions.pdf]

BMJ Open is committed to open peer review. As part of this commitment we make the peer review history of every article we publish publicly available.

When an article is published we post the peer reviewers' comments and the authors' responses online. We also post the versions of the paper that were used during peer review. These are the versions that the peer review comments apply to.

The versions of the paper that follow are the versions that were submitted during the peer review process. They are not the versions of record or the final published versions. They should not be cited or distributed as the published version of this manuscript.

BMJ Open is an open access journal and the full, final, typeset and author-corrected version of record of the manuscript is available on our site with no access controls, subscription charges or pay-per-view fees (http://bmjopen.bmj.com).

If you have any questions on BMJ Open's open peer review process please email info.bmjopen@bmj.com

BMJ Open

clinically SUspected ScaPhoid fracture: treatment with supportive bandage or CasT? "Study protocol of a multicenter randomized controlled trial" (SUSPECT study)

Journal:	*BMJ Open*
Manuscript ID	bmjopen-2020-036998
Article Type:	Protocol
Date Submitted by the Author:	14-Jan-2020

Complete List of Authors:	Cohen, Abigael; Erasmus MC, Department of Orthopaedics<br>Reijman, M; Erasmus MC, Department of Orthopaedics<br>Kraan, Gerald; Reinier de Graaf Hospital, Department of Orthopaedics<br>Mathijssen, Nina; Reinier de Graaf Hospital, Department of Orthopaedics<br>Koopmanschap, Marc; Erasmus University Rotterdam, Erasmus School of Health Policy and Management<br>Verhaar, Jan; Erasmus MC, Department of Orthopaedics<br>Mol, Sander; Franciscus Gasthuis en Vlietland, Department of Emergency Medicine<br>Colaris, Joost; Erasmus MC, Department of Orthopaedics<br>van der Zwaal, Peer; Medical Centre Haaglanden, Department of Orthopaedics<br>van Loon, Merel; Medical Centre Haaglanden, Department of Emergency Medicine<br>Rhemrev, Steven; Medical Centre Haaglanden, Department of Surgery<br>van Rijssel, Daphne; Reinier de Graaf Hospital, Department of Orthopaedics<br>de Vries, Mark; Reinier de Graaf Hospital, Department of Surgery<br>van Beek, Flip; Franciscus Gasthuis en Vlietland, Department of Surgery<br>Brown, Vanessa; Franciscus Gasthuis en Vlietland, Department of Emergency Medicine<br>Hagenaars, Tjebbe; Erasmus MC, Trauma Research Unit, Department of Surgery<br>Jansen, Els; Erasmus MC, Department of Emergency Medicine<br>Greeven, Alexander; Haga Teaching Hospital, Department of Surgery<br>Scholtens, Lenneke ; Haga Teaching Hospital, Department of Emergency Medicine<br>Deijkers, Ruud; Haga Teaching Hospital, Department of Orthopaedics<br>Schep, Niels W.L.; Maasstad Ziekenhuis, Department of Surgery<br>Ringburg, Akkie; Ikazia Ziekenhuis, Department of Surgery<br>Bruijninckx, Milko; IJsselland Hospital, Department of Surgery<br>van Meerten, Kirsten; Albert Schweitzer Hospital, Department of Emergency Medicine<br>Bosman, Willem-Maarten; Albert Schweitzer Hospital, Department of Surgery

Keywords:	Adult orthopaedics < ORTHOPAEDIC & TRAUMA SURGERY, Trauma management < ORTHOPAEDIC & TRAUMA SURGERY, Hand & wrist < ORTHOPAEDIC & TRAUMA SURGERY, ACCIDENT & EMERGENCY

	MEDICINE

I, the Submitting Author has the right to grant and does grant on behalf of all authors of the Work (as defined in the below author licence), an exclusive licence and/or a non-exclusive licence for contributions from authors who are: i) UK Crown employees; ii) where BMJ has agreed a CC-BY licence shall apply, and/or iii) in accordance with the terms applicable for US Federal Government officers or employees acting as part of their official duties; on a worldwide, perpetual, irrevocable, royalty-free basis to BMJ Publishing Group Ltd ("BMJ") its licensees and where the relevant Journal is co-owned by BMJ to the co-owners of the Journal, to publish the Work in this journal and any other BMJ products and to exploit all rights, as set out in our licence.

The Submitting Author accepts and understands that any supply made under these terms is made by BMJ to the Submitting Author unless you are acting as an employee on behalf of your employer or a postgraduate student of an affiliated institution which is paying any applicable article publishing charge ("APC") for Open Access articles. Where the Submitting Author wishes to make the Work available on an Open Access basis (and intends to pay the relevant APC), the terms of reuse of such Open Access shall be governed by a Creative Commons licence – details of these licences and which Creative Commons licence will apply to this Work are set out in our licence referred to above.

Other than as permitted in any relevant BMJ Author's Self Archiving Policies, I confirm this Work has not been accepted for publication elsewhere, is not being considered for publication elsewhere and does not duplicate material already published. I confirm all authors consent to publication of this Work and authorise the granting of this licence.

For peer review only

clinically SUspected ScaPhoid fracture: treatment with supportive bandage or CasT? "Study protocol of a multicenter randomized controlled trial" (SUSPECT study)

Abigael Cohen[1], Max Reijman[1] , Gerald A Kraan[2], Nina M C Mathijssen[2], Marc A Koopmanschap[3], Jan A N Verhaar[1], Sander Mol[5], Joost W Colaris[1], on behalf of the SUSPECT study group

SUSPECT study group:

Peer van der Zwaal[6], Merel van Loon[7], Steven J Rhemrev[8], Daphne A van Rijssel[9], Mark R de Vries[10], Flip van Beek[11], Vanessa Brown[5], Tjebbe Hagenaars[12], Els H Jansen[13], Alexander P A Greeven[14], Lenneke C M Scholtens[15], Ruud L M Deijkers[16], Niels W L Schep[17], Akkie N Ringburg[18], Milko M M Bruijninckx[19], Kirsten F van Meerten[20], Willem-Maarten P F Bosman[21]

1 Department of Orthopaedic Surgery, Erasmus MC University Medical Center, Rotterdam, The Netherlands

2 Department of Orthopaedic Surgery, Reinier de Graaf Hospital, Delft, The Netherlands

3 Erasmus School of Health Policy and Management, Erasmus University Rotterdam, Rotterdam, The Netherlands

5 Department of Emergency Medicine, Franciscus Hospital, Rotterdam, The Netherlands

6 Department of Orthopaedic Surgery, Haaglanden Medical Centre, The Hague, The Netherlands

7 Department of Emergency Medicine, Haaglanden Medical Centre, The Hague, The Netherlands

8 Department of Surgery, Haaglanden Medical Centre, The Hague, The Netherlands,

9 Department of Emergency Medicine, Reinier de Graaf Hospital, Delft, The Netherlands

10 Department of Surgery, Reinier de Graaf Hospital, Delft, The Netherlands

11 Department of Surgery, Franciscus Hospital, Rotterdam, The Netherlands

For peer review only

For peer review only - http://bmjopen.bmj.com/site/about/guidelines.xhtml

12 Trauma Research Unit, Department of Surgery, Erasmus MC University Medical Center, Rotterdam, The Netherlands

13 Department of Emergency Medicine, Erasmus MC University Medical Center, Rotterdam, The Netherlands

14 Department of Surgery, Haga Teaching Hospital, The Hague, The Netherlands

15 Department of Emergency Medicine, Haga Teaching Hospital, The Hague, The Netherlands

16 Department of Orthopaedic Surgery, Haga Teaching Hospital, The Hague, The Netherlands

17 Department of Surgery, Maasstad Hospital, Rotterdam, The Netherlands

18 Department of Surgery, Ikazia Hospital, Rotterdam, The Netherlands

19 Department of Surgery, Ijsselland Hospital, Capelle aan den Ijssel, The Netherlands

20 Department of Emergency Medicine, Albert Schweitzer Hospital, Dordrecht, The Netherlands

21 Department of Surgery, Albert Schweitzer Hospital, Dordrecht, The Netherlands

Corresponding Author: Abigael Cohen, Telephone +31107043982, Email: a.cohen.1@erasmusmc.nl Department of Orthopedic Surgery, Erasmus MC University Medical Center, PO box 2040, 3000 CA Rotterdam, the Netherlands

Trial sponsor: Prof. dr. J.A.N. Verhaar, Email: j.verhaar@erasmusmc.nl Department of Orthopedic Surgery, Erasmus University MC Medical Center, PO box 2040, 3000 CA Rotterdam, the Netherlands

Authors' Contributions

MR and JC conceived the study idea and secured funding. AC, MR, GK, NM, MK, JV, SM and JC were involved with the initial study design. MR and MK were involved with the statistical analysis for the study. AC, GK, SM, JC, SUSPECT study group are involved with the implementation of the protocol

For peer review only - http://bmjopen.bmj.com/site/about/guidelines.xhtml

and the data collection. AC is conducting the study. All authors provided critical comments to the manuscript and approved the final version before submitting.

Funding statement

For this study, we received a grant from ZonMw, a Dutch organization for health research and care innovation and co-funding from CZ, a Dutch health insurance company. Both organizations were not involved in the study design.

Competing interests

None of the authors declared competing interests.

Trial registration

This study was prospectively registered at the Netherlands National Trial Register on February 28th, 2018, (www.trialregister.nl, NL6976)

Word count: 3816

Keywords: Scaphoid Bone, Fractures, Bone, Recovery of Function, Cost-Benefit Analysis, Wrist injuries.

ABSTRACT

Introduction: Some scaphoid fractures become visible on radiographs weeks after a trauma which makes normal radiographs directly after trauma unreliable. Untreated scaphoid fractures can lead to scaphoid non-union progressing to osteoarthritis. Therefore, the general treatment for patients with a clinically suspected scaphoid fracture and normal initial radiographs, is immobilization with below-elbow cast for 2 weeks. However, most of these patients are treated unnecessary because eventually

For peer review only - http://bmjopen.bmj.com/site/about/guidelines.xhtml

less than 10% of them are diagnosed with an occult scaphoid fracture. To reduce overtreatment and costs as a result of unnecessary cast treatment in patients with a clinically suspected scaphoid fracture and normal initial radiographs, we designed a study to compare treatment with below-elbow cast or supportive bandage. We hypothesize that the functional outcome after 3 months is similar in both groups, but with lower costs in the supportive bandage group.

Methods and analysis: The SUSPECT study is an open-labeled multicenter randomized controlled trial with non-inferiority design. A total of 180 adult patients with a clinically suspected scaphoid fracture and normal initial radiographs are randomized between two groups: 3 days supportive bandage or 14 days below-elbow cast. We aim to evaluate the functional outcome and cost-effectiveness of both treatments. The primary outcome is the difference in functional outcome after 3 months between both groups, assessed with the Quick Disability of the Arm, Shoulder and Hand score. Secondary outcomes include post-traumatic injuries, functional outcome, recovery of function, pain, patient satisfaction, quality of life and cost-effectiveness measured by medical consumption, absence from work or decreased productivity.

Ethics and dissemination: The medical ethics committee of the Erasmus MC Medical Centre, Rotterdam approved the study protocol (MEC-2017-504). We plan to present the results after completion of the study at (inter)national conferences and publish in general peer-reviewed journals.

Trial registration: www.trialregister.nl, NL6976, February 28th, 2018 registered.

SRENGTHS AND LIMITATIONS OF THIS STUDY

- This is the first multicenter randomized controlled trial to evaluate if treatment with supportive bandage results in equal functional outcome as below-elbow cast in patients with a clinically suspected scaphoid fracture.
- All patients are re-evaluated clinically and radiographically after one year.

For peer review only

For peer review only - http://bmjopen.bmj.com/site/about/guidelines.xhtml

- The cost-effectiveness of the treatment up to one year is studied.
- The main limitation of the study is that the study is not blinded for participants, physicians, and researchers.

INTRODUCTION

Up to 32% of all scaphoid fractures results in non-union, leading to progressive osteoarthritis,(1). While most fractures are visible on radiographs directly after trauma, not all scaphoid fractures can be diagnosed on the initial radiographs. To avoid untreated occult scaphoid fractures, all patients with a clinically suspected scaphoid fracture and normal initial radiographs are treated with a below-elbow cast until re-examination or further diagnostics,(2, 3). Finally, less than 10% of these patients appear to have a scaphoid fracture resulting in a large number of overtreatment, unnecessary absenteeism from work and increased health care costs,(4, 5).

Most occult scaphoid fractures can be diagnosed on repeated conventional radiography after 10-14 days (6). These occult fractures are in general non-displaced fractures of the waist or distal pole of the scaphoid, in which an intact periosteal envelop provides stability,(7, 8). Known risk factors for scaphoid non-union are proximal fracture location, fracture displacement and start of treatment more than 4 weeks after trauma (8-10). When adequate treatment for a scaphoid fracture is started within 4 weeks after the trauma, the non-union rate does not increase,(8). Therefore, supportive bandage and re-examination within 2 weeks can be a good alternative treatment for patients with a clinically suspected scaphoid fracture with normal radiographs. In 1988, one randomized study of 106 patients with a clinically suspected scaphoid fracture showed that treatment with supportive bandage compared with below-elbow cast in these patients decreased the immobilization time and absence of work without negative consequences on fracture healing. However, functional outcome and cost-effectiveness were not evaluated, and the follow-up period was not clearly stated,(8).

We designed a randomized controlled trial with 1 year follow up to evaluate the functional outcome and cost-effectiveness of treatment with a supportive bandage compared with below-

For peer review only - http://bmjopen.bmj.com/site/about/guidelines.xhtml

elbow-cast in patients with a clinically suspected scaphoid fracture and normal initial radiographs. We hypothesize that treatment with supportive bandage results in an equal functional outcome compared to below-elbow cast, with lower costs in the supportive bandage group.

Objectives

We designed a multicenter randomized controlled trial with a non-inferiority design with two groups. The primary objective of this study is to determine if treatment with supportive bandage compared with below-elbow cast in patients with clinically suspected scaphoid fracture and normal initial radiographs, results in equal functional outcome after 3 months, measured with the Quick Disabilities of the Arm, Shoulder and Hand (QDASH).

As secondary objectives, we assess whether treatment of patients with a clinically suspected scaphoid fracture with a bandage is cost-effective compared to cast and we assess post-traumatic injuries, pain, patient satisfaction of the received treatment, functional outcome measured with the QDASH, recovery of function measured with physical examination and the Patient Rated Wrist/Hand Evaluation (PRWHE) and quality of life. Cost-effectiveness is evaluated by medical consumption, absence from work or decreased productivity and quality of life, measured in Quality-adjusted life years (QALYs).

METHODS AND ANALYSIS

This manuscript is written according to the Consolidated Standards for Reporting Trials (CONSORT statement) and Standard Protocol Items: Recommendations for Interventional Trials (SPIRIT guidelines),(11, 12).

Patient and Public Involvement statement

In the absence of a patient association, we formed a panel of patients with a (suspected) scaphoid fracture to think along with, and comment on our study. This panel of patients analyzed the study

For peer review only - http://bmjopen.bmj.com/site/about/guidelines.xhtml

(aim, burden for patient, willingness to participate), patient information sheet and how the future results should be reported to the patients. To improve our study, we evaluate the study procedures and the patient burden with participating patients during the recruitment period. After termination of the study, we present our study results to all participating patients.

Study design

The SUSPECT study is performed in 9 hospitals in the Netherlands. All departments involved in the treatment of patients with a clinically suspected scaphoid fracture in each participating hospital participate in the study.

All patients who present with radial sided wrist pain after trauma at the emergency department (ED), who match the in- and exclusion criteria, are informed about the study by their treating physician. These patients receive the participant information sheet and are invited to ask questions about the study. As direct start of treatment is necessary, patients have to decide on study participation after the information is provided at the ED. Written informed consent is obtained from the patient prior to inclusion. All included patients are randomized at the ED and allocated to treatment with supportive bandage or below-elbow cast.

Patients revisit the outpatient clinic after 14 days and 1 year. After 14 days, the researcher re-examines the patient first. Secondly, radiographs with scaphoid specific views are obtained (without cast or bandage around the wrist) according to the hospital protocol. Lastly, the physician re-examines the patient and determines the diagnosis, treatment and follow-up without interference of the researcher according to their hospital protocol. After 1 year, the researcher re-examines the patients and wrist radiographs are made.

During the follow up period, patients receive questionnaires by email after inclusion, 2 weeks, 6 weeks, 3 months, 6 months, 9 months and 1 year of follow up.

For peer review only

For peer review only - http://bmjopen.bmj.com/site/about/guidelines.xhtml

Due to the Dutch privacy law, it is not allowed to screen eligible patients at the ED to detect how many eligible patients are not participating in our study.

Study population

All adult patients who visit the ED of the participating hospitals, within 48 hours after trauma with a clinically suspected scaphoid fracture without a fracture on the radiograph, are invited to participate in the study.

Inclusion criteria

- Age of 18 years or older
- Trauma maximum 48 hours before
- Anatomical snuff box (ASB) tenderness or scaphoid tubercle (ST) tenderness
- No scaphoid fracture reported by the radiologist or treating physician on the initial radiographs

Exclusion criteria

- Concomitant injury of the ipsilateral extremity that needs treatment with movement restriction, cast or supportive bandage.
- Inability to complete study forms due to insufficient command of the Dutch language.
- A scaphoid fracture diagnosed on the initial radiographs by a supervising radiologist in retrospect.

Randomization

For peer review only - http://bmjopen.bmj.com/site/about/guidelines.xhtml

After written informed consent is obtained, patients are randomized through computer-based variable block randomization (2, 4, 6 blocks) by Castor Electronic Data Capture (EDC),(13). The allocation ratio is 1:1 and randomization is stratified per hospital due to potential differences in applied cast and the number of scaphoid specific views per hospital.

Because of practical reasons, there is no blinding of the treatment group for the physician, patient and researcher, but the radiologist is blinded.

Interventions

Intervention group (supportive bandage)

Patients in the intervention group receive a below-elbow supportive bandage for 3 days at the ED. After 3 days the patients are allowed to remove the bandage and move the wrist driven by the amount of pain. When the pain is too severe despite adequate analgesia, an additional appointment is made at the outpatient clinic to apply a below-elbow cast for analgesic purposes.

Control group (below-elbow cast)

The usual care in the Netherlands for patients with clinically suspected scaphoid fracture at the ED is below-elbow cast and re-examination after 14 days (Figure 1),(2). The applied below-elbow cast type is according to hospital protocol and can be circular or splint, with or without the thumb included.

Outcomes

Outcome

At baseline we collect the following variables; demographic data (e.g. age, gender, height, weight, hand dominance), details about the trauma (e.g. fall onto an outstretched hand (FOOSH),

For peer review only - http://bmjopen.bmj.com/site/about/guidelines.xhtml

For peer review only

high-energy trauma, low-energy trauma, sports injury) and comorbidities such as smoking status, previous arm injuries and general medical history.

Primary outcome

The primary outcome is the difference in functional outcome between the intervention and control group measured with the QDASH after 3 months of follow-up. We selected the QDASH as the primary outcome, as it takes the function of both hands into account. With this information, we can reflect if the wrist problem results in an impaired functional outcome in daily life.

Secondary outcomes

- Functional outcome measured with the QDASH after inclusion and at 2 weeks, 6 weeks and 1 year of follow up (Table 1).
- Recovery of function evaluated with PRWHE after inclusion and at 2 weeks, 6 weeks, 3 months and 1 year of follow up.
- The amount of pain scored with the Visual Analogue Scale (VAS) score at rest and during movement after inclusion and at 2 weeks, 6 weeks, 3 months and 1 year of follow up.
- Patient satisfaction of the treatment is measured after 2 weeks and 3 months of follow up.
- Quality of life assessed by the 5-level EuroQol-5D (EQ-5D-5L) after inclusion and at 2 weeks, 6 weeks, 3 months and 1 year of follow up. When the patient reports medical consumption or decreased productivity due the wrist problems after 6 or 9 months of follow up, the EQ-5D-5L is sent to the patients as well.
- To assess the costs and cost-effectiveness of both interventions, we collect information on diagnostic and therapeutic hospital procedures using the electronic patient status and questionnaires. We use iMTA Medical Consumption Questionnaire (iMCQ) and iMTA

For peer review only

For peer review only - http://bmjopen.bmj.com/site/about/guidelines.xhtml

Productivity Cost Questionnaire (iPCQ) after 6 weeks, 3 months, 6 months, 9 months and 1 year of follow up (Table 1).

- Physical examination is performed at inclusion, after 2 weeks and 1 year of follow up. At inclusion, the physical examination is performed by the treating physician. They will register if the patient has tenderness ASB, tenderness ST, compression pain thumb, radial sided wrist pain with resisted supination and radial sided wrist pain with ulnar deviation. A previous study showed that these clinical tests have the highest sensitivity for a scaphoid fracture,(14). After 2 weeks and 1 year, the function of the wrist is assessed by the researcher. The physical examination includes the abovementioned clinical tests and swelling or hematoma on the radial side of the wrist. Range of motion of the wrist (palmar flexion, dorsal flexion, ulnar deviation, radial deviation, supination, and pronation) and finger-to-palm distance are measured with a goniometer. The finger-to-palm distance is the distance from the tip of the finger to the distal palmar crease, when the fingers are in maximal active flexion,(15). We assess opposition of the thumb with the Kapandji score,(16). The Kapandji score ranges from 0 – 10, whereas 0 indicates no opposition and 10 maximal opposition. Grip strength is measured with a hand dynamometer (Jamar hand dynamometer), and is defined as the maximum grip strength after 3 attempts. Range of motion and grip strength of both wrists are examined. One year after inclusion, we additional test the scapholunate (SL) ligament by identifying tenderness over the dorsal SL interval, performing the finger extension test, and the Watson test (Table 2). The finger extension test is positive when resisted extension of the fingers with the wrist in flexion is painful,(17). To perform the Watson test, the patients' wrist is in dorsal flexion and ulnar deviation, with the researchers thumb on ST and fingers wrapped around the distal radius. Then, the researcher moves the patients' wrist radially and in palmar flexion. The Watson test is positive if a painful clunk is noticed,(17, 18).

For peer review only

For peer review only - http://bmjopen.bmj.com/site/about/guidelines.xhtml

- Radiographic re-examination is performed after 2 weeks and 1 year of follow up. After 2 weeks, the radiographs include scaphoid specific views to identify a scaphoid fracture. After 1 year, a posterior-anterior and lateral wrist radiographs are made to determine the presence of any post-traumatic injuries (e.g. scaphoid non-union, SL dissociation).

Table 1 Description of patient-rated outcome measures and examinations per follow up moment.

	Baseline	2 weeks	6 weeks	3 months	6 months	9 months	1 year
**Radiograph**	Scaphoid	Scaphoid					Wrist
**Physical examination**	x	x					x
**PROM**	QDASH<br>PRWHE<br>VAS<br>EQ-5D-5L	QDASH<br>PRWHE<br>VAS<br>Patient satisfaction<br>EQ-5D-5L	QDASH<br>PRWHE<br>VAS<br>EQ-5D-5L<br>iMCQ<br>iPCQ	QDASH<br>PRWHE<br>VAS<br>Patient satisfaction<br>EQ-5D-5L<br>iMCQ<br>iPCQ	iMCQ<br>iPCQ<br>(EQ-5D-5L)	iMCQ<br>iPCQ<br>(EQ-5D-5L)	QDASH<br>PRWHE<br>VAS<br>EQ-5D-5L<br>iMCQ<br>iPCQ

PROM, Patient-Rated Outcome Measure; QDASH, *Quick Disability of the Arm, Shoulder and Hand; PRWHE, Patient Rated Wrist Hand Evaluation; VAS, Visual Analog Score; EQ-5D-5L, 5-level EuroQol-5D; iMCQ, iMTA Medical Consumption Questionnaire; iPCQ, iMTA Productivity Cost Questionnaire.*

For peer review only

For peer review only - http://bmjopen.bmj.com/site/about/guidelines.xhtml

**Table 2** Physical examination per follow up moment			
	Baseline	2 weeks	1 year
Swelling radial side wrist		x	x
Hematoma radial side wrist		x	x
Tenderness ASB	x	x	x
Tenderness ST	x	x	x
Compression pain thumb	x	x	x
Painful resisted supination	x	x	x
Painful UD	x	x	x
SL ligament			x
Kapandji score		x	x
Range of motion		x	x
Finger-to-palm distance		x	x
Grip strength		x	x

UD, Ulnar deviation; ASB, *Anatomical snuff box; ST, Scaphoid Tubercle; SL, Scapholunate ligament examinations consist of tenderness SL interval, Finger extension test and Watson test. Range of motion include palmar flexion, dorsal flexion, ulnar deviation, radial deviation, supination and pronation.*

Instruments

Functional outcome is measured with the mandatory module of the QDASH. The QDASH is the short version of the DASH and consists of 11 questions about symptoms and physical function of both arms during the last week. Each question can be scored from 1 - 5. A high score indicates severe disability of the upper extremity. The Dutch QDASH has shown good validity, reliability and responsiveness for comparable patients,(19, 20).

The PRWHE questionnaire evaluates the painful wrist during the last week. The questionnaire consists of three subscales about pain, function, and cosmetics. The cosmetic field is not included in the score. The pain and function subscales consist each of 15 questions that can be scored from 0-10. A high PRWHE score indicates severe pain and restricted function of the painful wrist. The Dutch PRWHE has shown good validity, reliability and responsiveness for comparable patients,(20) .

For peer review only - http://bmjopen.bmj.com/site/about/guidelines.xhtml

304 Pain scores at rest and during movement are examined with the elevenpoint Visual Analogue
305 Scale (VAS) for pain. A high VAS score indicates severe pain. The VAS for pain is widely used in
306 research with comparable patients,(21).

307 Patient satisfaction is assessed by the questions 'how satisfied are you with the treatment',
308 'did you prefer the other treatment?', and 'if yes, why?'. The first question can be scored from 0-10.
309 A high score indicates satisfaction.

310 Quality of life is measured with the EQ-5D-5L. This questionnaire consists of five questions and
311 a visual analogue scale. The questions are about mobility, self-care, usual activities, pain or
312 discomfort, and anxiety or depression. Each question can be scored as: no problem, slight problem,
313 moderate problem, severe problem, and extreme problem. Health is reported on a visual analogue
314 scale from 0-100. A high score indicates better health. The EQ-5D-5L is recommended for the
315 assessment of quality of life in trauma patients, especially for economic assessments,(22).

316 To assess the costs and cost-effectiveness of the intervention with the usual care we include
317 the Medical Consumption Questionnaire (iMCQ) and Productivity Cost Questionnaire (iPCQ). The
318 iMCQ measures the total medical consumption by measuring the use of diagnostics, consultations,
319 emergency department visits, physical therapy, medication, and aids. The iPCQ measures
320 productivity costs. This questionnaire evaluates costs of absence of work and decreased productivity
321 at paid or unpaid work. The iMCQ and iPCQ s are short generic measurement instruments,(23).

322

323 **Sample size**

324 Our hypothesis is that patients with a clinically suspected scaphoid fracture with normal initial
325 radiographs treated with a bandage have minimally equal functional outcome after 3 months
326 compared with cast, but with lower costs. The power calculation is based on the proof of non-
327 inferiority. We took 50% of the margin of clinical minimal important difference of QDASH,(24). This
328 resulted in a margin of non-inferiority of 7.50 points on the QDASH. The standard deviation of

For peer review only - http://bmjopen.bmj.com/site/about/guidelines.xhtml

QDASH after 3 months has been reported to be 14,(25). We used a power of 90% and a one-sided alpha of 0.025. To detect non-inferiority of bandage compared to cast 148 patients are needed (74 patients per group). Accounting for a 15% lost to follow-up, a total of 180 patients are required. When the lost to follow-up or change of allocated treatment is higher than 15%, we will include more patients to have 74 patients per group to answer our primary research question. When the patient recruitment rate at the ED is behind our expectations, we invite more hospitals to participate.

Data collection

We will collect data by electronic data capture with Castor EDC,(13). Paper based case report forms are used at the ED and the outpatient department. The researcher inserts this data in Castor EDC. Questionnaires are sent directly to the patients through email via Castor EDC. In case an email address is not available, a paper case report form is sent to the patient. When the patient does not respond, we will contact the patient by telephone.

We screen the electronic patient status to collect information about (serious) adverse events, hospital visits, radiographic examinations and treatment for the wrist injury during the follow-up period of the study.

Data analysis

The primary analysis is 'by intention to treat analysis'. The distribution analysis of all variables is tested by the Shapiro-Wilk test. For normally distributed variables, parametric tests are used. For those variables that are not normally distributed, non-parametric tests will be used.

The difference in functional outcome measured with the QDASH after 3 months follow-up between the two groups is the primary outcome. The primary analyses are performed with a linear regression, with the QDASH score at 3 months follow-up as dependent variable and the allocated intervention as independent variable. A margin of non-inferiority of 7.50 is used in the analyses.

For peer review only - http://bmjopen.bmj.com/site/about/guidelines.xhtml
For peer review only

Adjustment are done for those baseline variables that change the effect estimate with more than 10%.

As secondary outcomes we evaluate the difference in PRWHE, VAS score, EQ5D-5L and physical examination between the two groups after 3 months follow up with a linear regression. Furthermore, we assess the differences in scores of the QDASH, PRWHE, VAS, EQ5D-5L and physical examination at the follow-up time points with repeated measurements in a linear mixed model. Fixed effects are time, and the covariates we adjust for. For patients who are lost to follow-up, we include all observed data in the analysis. Adjustments are made for those baseline variables that change the effect estimate with more than 10%. The assumptions of constant variance and linear relationships are assessed. Should any of these assumptions seriously fail, variable transformations are used.

Cost-effectiveness analysis (CEA)

General considerations

We assess the cost-effectiveness of 3 days supportive bandage versus 14 days below-elbow cast over a period of 12 months. To answer this question, we perform a cost-utility analysis to estimate the incremental cost per QALY gained, to be able to compare our study with other studies in musculoskeletal disorders and in other disease categories. Using non-parametric bootstrapping (randomly drawing 5000 observations with replacement from the patient sample), the degree of uncertainty for costs and health effects and the cost-utility ratio are depicted in a cost-effectiveness plane. In addition, an acceptability curve is drawn, which indicates the probability that the intervention studied has lower incremental costs per QALY gained than various thresholds (dependent on disease severity) for the maximum willingness to pay for an extra QALY.

Cost analysis

For peer review only

For peer review only - http://bmjopen.bmj.com/site/about/guidelines.xhtml

The economic analysis is based on the societal perspective, and on the healthcare perspective in which the direct medical and productivity costs between the groups are compared. To measure the direct costs, an inventory of patients total medical consumption is made using the electronic patient status and the iMCQ. The use of diagnostics, consultations, emergency department visits, physical therapy, medication and aids are evaluated. Productivity costs are measured with the iPCQ. Costs of absence of work and decreased productivity at a paid job or at unpaid work are evaluated. The friction cost method is used to calculate the productivity costs according to the recent Dutch guidelines. The costs per unit of medical consumption is estimated, using information from the most recent Dutch Manual for economic evaluation of health care. Costs are reported for the year 2019.

Cost-utility analysis

Patient outcomes relevant for the CEA are the number of QALYs during 1 year. Patient scores on the EQ 5D-5 level version are converted into utility values, using the valuation algorithm of the Dutch general population.

Budget impact analysis (BIA)

Guidelines of the ISPOR Task Force are used for the BIA. Relevant features and tariffs of the Dutch health care system, anticipated uptake of the new intervention as well as usual care are taken into account. The size and the eligibility of the population, cost of diagnostics, treatment modalities and changes expected in condition-related costs are considered in the BIA. Sensitivity analyses are performed using divergent scenarios from the viewpoint of the decision-makers.

The budget impact per year of implementing the new intervention is estimated. All elements of medical costs for the new intervention as well as for usual care that are paid by third-party payers are considered and calculated.

For peer review only - http://bmjopen.bmj.com/site/about/guidelines.xhtml

ETHICS AND DISSEMINATION

Safety considerations

The study compares treatment of below-elbow cast (standard treatment) with supportive bandage (intervention). Since the study is labeled as low risk, a data safety monitoring board is not required. The study is monitored at least once a year and the progress of the trial is reported once a year to the accredited medical review ethics committee (MEC). Adverse events are recorded by the researcher during follow up. A serious adverse event is reported (www.toetsingonline.nl) within a maximum of 15 days after the researcher is notified.

Adverse events are defined as:

- Cast related problems
- Bandage related problems
- Loss of function
- Complex regional pain syndrome
- Persisting pain after 6 weeks
- Conservative treated scaphoid non-union

Serious adverse events related to our study are defined as:

- Surgically treated scaphoid fracture
- Compartment syndrome
- Surgically treated scaphoid non-union

Because of the multicenter study design, clinical trial site agreements are obtained between the initiating and the participating hospitals. These agreements include liability and insurance aspects.

For peer review only - http://bmjopen.bmj.com/site/about/guidelines.xhtml

The patient information sheet reports on participants insurance and potential complications as a result of participation in the study. Withdrawal from the study is always possible for included patients without any consequences.

Ethics

The study is approved by the MEC of the Erasmus Medical Centrum, Rotterdam, the Netherlands (MEC-2017-504). All amendments are notified to the MEC and changes to the study are made after a favorable opinion of the MEC.

The study is conducted according to the principles of the Declaration of Helsinki (64th version, date: October 2013) and in accordance with the Medical Research Involving Human Subjects Act (WMO) and other guidelines, regulations, and acts.

The study is registered in the Dutch Clinical trials Registry (www.trialregister.nl, trial NL6976).

Participating patients and physicians are not paid for participation in the study.

Data management

Personal data of the participating patients that we collect during the study, is changed to a study number. This study number is used for all study documentation, study reports and publications.

Data is collected with Castor EDC. Paper case report forms are entered in Castor EDC by the researcher. The paper case report forms are securely filed in the hospital. All data is stored for 15 years. The final trial dataset is accessible for the research team.

Dissemination

After completion of the study, we plan to present the results at (inter)national congresses and submit the manuscript to general peer-review journals.

For peer review only - http://bmjopen.bmj.com/site/about/guidelines.xhtml

Authorship is according to the International Committee of Medical Journal Editors guidelines and a SUSPECT study group is made.

REFERENCES

1. Inoue G, Sakuma M. The natural history of scaphoid non-union. Radiographical and clinical analysis in 102 cases. ARCH ORTHOP TRAUMA SURG. 1996;115(1):1-4.
2. Mallee WH, Veltman ES, Doornberg JN, Blankevoort L, van Dijk CN, Goslings JC. [Variations in management of suspected scaphoid fractures]
Variatie in beleid bij vermoeden op scafoidfractuur. Ned Tijdschr Geneeskd. 2012;156(28):A4514.
3. Borel C, Larbi A, Delclaux S, Lapegue F, Chiavassa-Gandois H, Sans N, et al. Diagnostic value of cone beam computed tomography (CBCT) in occult scaphoid and wrist fractures. Eur J Radiol. 2017;97:59-64.
4. Bergh TH, Steen K, Lindau T, Soldal LA, Bernardshaw SV, Lunde L, et al. Costs analysis and comparison of usefulness of acute MRI and 2 weeks of cast immobilization for clinically suspected scaphoid fractures. Acta Orthop. 2015;86(3):303-9.
5. de Zwart AD, Beeres FJ, Rhemrev SJ, Bartlema K, Schipper IB. Comparison of MRI, CT and bone scintigraphy for suspected scaphoid fractures. Eur J Trauma Emerg Surg. 2016;42(6):725-31.
6. Gemme S, Tubbs R. What physical examination findings and diagnostic imaging modalities are most useful in the diagnosis of scaphoid fractures? Ann Emerg Med. 2015;65(3):308-9.
7. DaCruz DJ, Bodiwala GG, Finlay DB. The suspected fracture of the scaphoid: a rational approach to diagnosis. Injury. 1988;19(3):149-52.
8. Sjølin SU, Andersen JC. Clinical fracture of the carpal scaphoid—supportive bandage or plaster cast immobilization? The Journal of Hand Surgery: British & European Volume. 1988;13(1):75-6.
9. Langhoff O, Andersen JL. Consequences of late immobilization of scaphoid fractures. J Hand Surg Br. 1988;13(1):77-9.
10. Leslie IJ, Dickson RA. The fractured carpal scaphoid. Natural history and factors influencing outcome. J Bone Joint Surg Br. 1981;63-B(2):225-30.
11. Schulz KF, Altman DG, Moher D. CONSORT 2010 Statement: updated guidelines for reporting parallel group randomised trials. Bmj. 2010;340:c332.
12. Chan AW, Tetzlaff JM, Gotzsche PC, Altman DG, Mann H, Berlin JA, et al. SPIRIT 2013 explanation and elaboration: guidance for protocols of clinical trials. Bmj. 2013;346:e7586.
13. Ciwit BV. Castor Electronic Data Capture. Amsterdam2016.
14. Mallee WH, Henny EP, van Dijk CN, Kamminga SP, van Enst WA, Kloen P. Clinical diagnostic evaluation for scaphoid fractures: a systematic review and meta-analysis. J Hand Surg Am. 2014;39(9):1683-91 e2.
15. Torok KS, Baker NA, Lucas M, Domsic RT, Boudreau R, Medsger TA, Jr. Reliability and validity of the delta finger-to-palm (FTP), a new measure of finger range of motion in systemic sclerosis. Clin Exp Rheumatol. 2010;28(2 Suppl 58):S28-36.
16. Kapandji A. [Clinical test of apposition and counter-apposition of the thumb]
Cotation clinique de l'opposition et de la contre-opposition du pouce. Ann Chir Main. 1986;5(1):67-73.
17. Skirven T. Clinical examination of the wrist. Journal of Hand Therapy. 1996;9(2):96-107.

For peer review only - http://bmjopen.bmj.com/site/about/guidelines.xhtml

18. Walsh JJ, Berger RA, Cooney WP. Current status of scapholunate interosseous ligament injuries. JAAOS-Journal of the American Academy of Orthopaedic Surgeons. 2002;10(1):32-42.
19. Tsang P, Walton D, Grewal R, MacDermid J. Validation of the QuickDASH and DASH in Patients With Distal Radius Fractures Through Agreement Analysis. Arch Phys Med Rehabil. 2017;98(6):1217-22 e1.
20. Kleinlugtenbelt YV, Krol RG, Bhandari M, Goslings JC, Poolman RW, Scholtes VAB. Are the patient-rated wrist evaluation (PRWE) and the disabilities of the arm, shoulder and hand (DASH) questionnaire used in distal radial fractures truly valid and reliable? Bone Joint Res. 2018;7(1):36-45.
21. Badalamente M, Coffelt L, Elfar J, Gaston G, Hammert W, Huang J, et al. Measurement scales in clinical research of the upper extremity, part 1: general principles, measures of general health, pain, and patient satisfaction. J Hand Surg Am. 2013;38(2):401-6; quiz 6.
22. Hung M-C, Lu W-S, Chen S-S, Hou W-H, Hsieh C-L, Wang J-D. Validation of the EQ-5D in Patients with Traumatic Limb Injury. Journal of Occupational Rehabilitation. 2015;25(2):387-93.
23. Bouwmans C, Krol M, Severens H, Koopmanschap M, Brouwer W, Hakkaart-van Roijen L. The iMTA Productivity Cost Questionnaire: A Standardized Instrument for Measuring and Valuing Health-Related Productivity Losses. Value Health. 2015;18(6):753-8.
24. Franchignoni F, Vercelli S, Giordano A, Sartorio F, Bravini E, Ferriero G. Minimal clinically important difference of the disabilities of the arm, shoulder and hand outcome measure (DASH) and its shortened version (QuickDASH). J Orthop Sports Phys Ther. 2014;44(1):30-9.
25. Buijze GA, Goslings JC, Rhemrev SJ, Weening AA, Van Dijkman B, Doornberg JN, et al. Cast immobilization with and without immobilization of the thumb for nondisplaced and minimally displaced scaphoid waist fractures: a multicenter, randomized, controlled trial. J Hand Surg Am. 2014;39(4):621-7.

Author's contributions:

MR and JC conceived the study idea and secured funding. AC, MR, GK, NM, MK, JV, SM and JC were involved with the initial study design. MR and MK were involved with the statistical analysis for the study. AC, GK, SM, JC, SUSPECT study group are involved with the implementation of the protocol and the data collection. AC is conducting the study. All authors provided critical comments to the manuscript and approved the final version before submitting.

Funding

For this study, we received a grant from ZonMw, a Dutch organization for health research and care innovation and co-funding from CZ, a Dutch health insurance company. Both organizations were not involved in the study design.

Trial status

For peer review only

For peer review only - http://bmjopen.bmj.com/site/about/guidelines.xhtml

We are currently recruiting patients for the study. The date of first enrollment was 7 june 2018.

Competing interests statement

The authors declare to have no competing interests.

Legend:

Figure 1 Treatment of intervention and control group

For peer review only - http://bmjopen.bmj.com/site/about/guidelines.xhtml
For peer review only

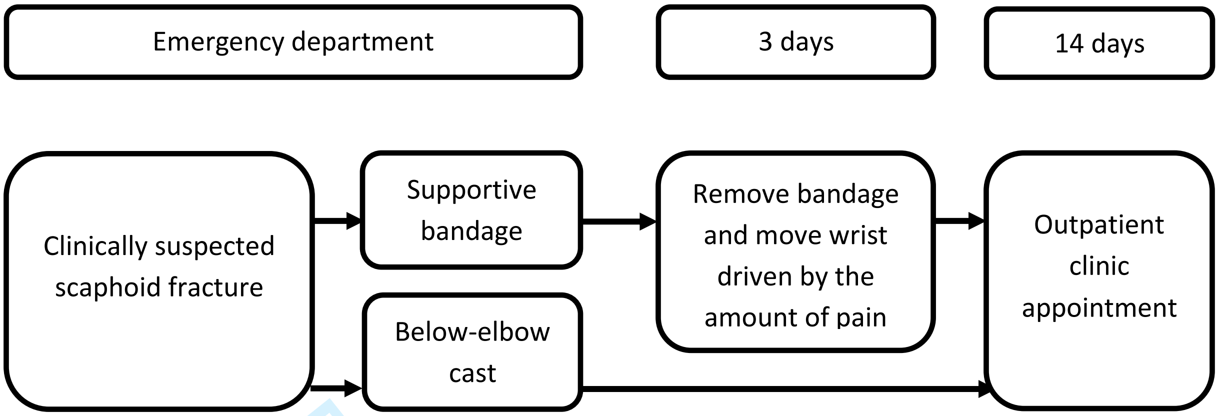

For peer review only

SPIRIT

STANDARD PROTOCOL ITEMS: RECOMMENDATIONS FOR INTERVENTIONAL TRIALS

SPIRIT 2013 Checklist: Recommended items to address in a clinical trial protocol and related documents*

Section/item	ItemNo	Description	Page
**Administrative information**			
Title	1	Descriptive title identifying the study design, population, interventions, and, if applicable, trial acronym	1
Trial registration	2a	Trial identifier and registry name. If not yet registered, name of intended registry	3
	2b	All items from the World Health Organization Trial Registration Data Set	n/a
Protocol version	3	Date and version identifier	footer
Funding	4	Sources and types of financial, material, and other support	3
Roles and responsibilities	5a	Names, affiliations, and roles of protocol contributors	1-3
	5b	Name and contact information for the trial sponsor	2
	5c	Role of study sponsor and funders, if any, in study design; collection, management, analysis, and interpretation of data; writing of the report; and the decision to submit the report for publication, including whether they will have ultimate authority over any of these activities	3
	5d	Composition, roles, and responsibilities of the coordinating centre, steering committee, endpoint adjudication committee, data management team, and other individuals or groups overseeing the trial, if applicable (see Item 21a for data monitoring committee)	n/a

			Page
**Introduction**			
Background and rationale	6a	Description of research question and justification for undertaking the trial, including summary of relevant studies (published and unpublished) examining benefits and harms for each intervention	5-6
	6b	Explanation for choice of comparators	5-6
Objectives	7	Specific objectives or hypotheses	6
Trial design	8	Description of trial design including type of trial (eg, parallel group, crossover, factorial, single group), allocation ratio, and framework (eg, superiority, equivalence, noninferiority, exploratory)	6
**Methods: Participants, interventions, and outcomes**			
Study setting	9	Description of study settings (eg, community clinic, academic hospital) and list of countries where data will be collected. Reference to where list of study sites can be obtained	7
Eligibility criteria	10	Inclusion and exclusion criteria for participants. If applicable, eligibility criteria for study centres and individuals who will perform the interventions (eg, surgeons, psychotherapists)	8
Interventions	11a	Interventions for each group with sufficient detail to allow replication, including how and when they will be administered	9
	11b	Criteria for discontinuing or modifying allocated interventions for a given trial participant (eg, drug dose change in response to harms, participant request, or improving/worsening disease)	9
	11c	Strategies to improve adherence to intervention protocols, and any procedures for monitoring adherence (eg, drug tablet return, laboratory tests)	9
	11d	Relevant concomitant care and interventions that are permitted or prohibited during the trial	9

For peer review only - http://bmjopen.bmj.com/site/about/guidelines.xhtml

			Page
Outcomes	12	Primary, secondary, and other outcomes, including the specific measurement variable (eg, systolic blood pressure), analysis metric (eg, change from baseline, final value, time to event), method of aggregation (eg, median, proportion), and time point for each outcome. Explanation of the clinical relevance of chosen efficacy and harm outcomes is strongly recommended	9-13
Participant timeline	13	Time schedule of enrolment, interventions (including any run-ins and washouts), assessments, and visits for participants. A schematic diagram is highly recommended (see Figure)	Figure 1 Table 1 Table 2
Sample size	14	Estimated number of participants needed to achieve study objectives and how it was determined, including clinical and statistical assumptions supporting any sample size calculations	14-15
Recruitment	15	Strategies for achieving adequate participant enrolment to reach target sample size	15
**Methods: Assignment of interventions (for controlled trials)**			
Allocation:			
Sequence generation	16a	Method of generating the allocation sequence (eg, computer-generated random numbers), and list of any factors for stratification. To reduce predictability of a random sequence, details of any planned restriction (eg, blocking) should be provided in a separate document that is unavailable to those who enrol participants or assign interventions	9
Allocation concealment mechanism	16b	Mechanism of implementing the allocation sequence (eg, central telephone; sequentially numbered, opaque, sealed envelopes), describing any steps to conceal the sequence until interventions are assigned	9
Implementation	16c	Who will generate the allocation sequence, who will enrol participants, and who will assign participants to interventions	7
Blinding (masking)	17a	Who will be blinded after assignment to interventions (eg, trial participants, care providers, outcome assessors, data analysts), and how	9

For peer review only - http://bmjopen.bmj.com/site/about/guidelines.xhtml

	17b	If blinded, circumstances under which unblinding is permissible, and procedure for revealing a participant's allocated intervention during the trial	n/a
**Methods: Data collection, management, and analysis**			
Data collection methods	18a	Plans for assessment and collection of outcome, baseline, and other trial data, including any related processes to promote data quality (eg, duplicate measurements, training of assessors) and a description of study instruments (eg, questionnaires, laboratory tests) along with their reliability and validity, if known. Reference to where data collection forms can be found, if not in the protocol	9-14
	18b	Plans to promote participant retention and complete follow-up, including list of any outcome data to be collected for participants who discontinue or deviate from intervention protocols	15-16
Data management	19	Plans for data entry, coding, security, and storage, including any related processes to promote data quality (eg, double data entry; range checks for data values). Reference to where details of data management procedures can be found, if not in the protocol	19
Statistical methods	20a	Statistical methods for analysing primary and secondary outcomes. Reference to where other details of the statistical analysis plan can be found, if not in the protocol	15-17
	20b	Methods for any additional analyses (eg, subgroup and adjusted analyses)	15-17
	20c	Definition of analysis population relating to protocol non-adherence (eg, as randomised analysis), and any statistical methods to handle missing data (eg, multiple imputation)	16
**Methods: Monitoring**			
Data monitoring	21a	Composition of data monitoring committee (DMC); summary of its role and reporting structure; statement of whether it is independent from the sponsor and competing interests; and reference to where further details about its charter can be found, if not in the protocol. Alternatively, an explanation of why a DMC is not needed	18

For peer review only - http://bmjopen.bmj.com/site/about/guidelines.xhtml

			Page
	21b	Description of any interim analyses and stopping guidelines, including who will have access to these interim results and make the final decision to terminate the trial	n/a
Harms	22	Plans for collecting, assessing, reporting, and managing solicited and spontaneously reported adverse events and other unintended effects of trial interventions or trial conduct	18
Auditing	23	Frequency and procedures for auditing trial conduct, if any, and whether the process will be independent from investigators and the sponsor	18
**Ethics and dissemination**			
Research ethics approval	24	Plans for seeking research ethics committee/institutional review board (REC/IRB) approval	19
Protocol amendments	25	Plans for communicating important protocol modifications (eg, changes to eligibility criteria, outcomes, analyses) to relevant parties (eg, investigators, REC/IRBs, trial participants, trial registries, journals, regulators)	19
Consent or assent	26a	Who will obtain informed consent or assent from potential trial participants or authorised surrogates, and how (see Item 32)	7
	26b	Additional consent provisions for collection and use of participant data and biological specimens in ancillary studies, if applicable	n/a
Confidentiality	27	How personal information about potential and enrolled participants will be collected, shared, and maintained in order to protect confidentiality before, during, and after the trial	19
Declaration of interests	28	Financial and other competing interests for principal investigators for the overall trial and each study site	21
Access to data	29	Statement of who will have access to the final trial dataset, and disclosure of contractual agreements that limit such access for investigators	19
Ancillary and post-trial care	30	Provisions, if any, for ancillary and post-trial care, and for compensation to those who suffer harm from trial participation	18

For peer review only - http://bmjopen.bmj.com/site/about/guidelines.xhtml

			Page
Dissemination policy	31a	Plans for investigators and sponsor to communicate trial results to participants, healthcare professionals, the public, and other relevant groups (eg, via publication, reporting in results databases, or other data sharing arrangements), including any publication restrictions	19
	31b	Authorship eligibility guidelines and any intended use of professional writers	21
	31c	Plans, if any, for granting public access to the full protocol, participant-level dataset, and statistical code	n/a
**Appendices**			
Informed consent materials	32	Model consent form and other related documentation given to participants and authorised surrogates	
Biological specimens	33	Plans for collection, laboratory evaluation, and storage of biological specimens for genetic or molecular analysis in the current trial and for future use in ancillary studies, if applicable	n/a

*It is strongly recommended that this checklist be read in conjunction with the SPIRIT 2013 Explanation & Elaboration for important clarification on the items. Amendments to the protocol should be tracked and dated. The SPIRIT checklist is copyrighted by the SPIRIT Group under the Creative Commons "Attribution-NonCommercial-NoDerivs 3.0 Unported" license.

For peer review only - http://bmjopen.bmj.com/site/about/guidelines.xhtml

BMJ Open

clinically SUspected ScaPhoid fracture: treatment with supportive bandage or CasT? “Study protocol of a multicenter randomized controlled trial” (SUSPECT study)

Journal:	*BMJ Open*
Manuscript ID	bmjopen-2020-036998.R1
Article Type:	Protocol
Date Submitted by the Author:	18-Jun-2020
Complete List of Authors:	Cohen, Abigael; Erasmus MC, Department of Orthopaedics<br>Reijman, M; Erasmus MC, Department of Orthopaedics<br>Kraan, Gerald; Reinier de Graaf Hospital, Department of Orthopaedics<br>Mathijssen, Nina; Reinier de Graaf Hospital, Department of Orthopaedics<br>Koopmanschap, Marc; Erasmus University Rotterdam, Erasmus School of Health Policy and Management<br>Verhaar, Jan; Erasmus MC, Department of Orthopaedics<br>Mol, Sander; Franciscus Gasthuis en Vlietland, Department of Emergency Medicine<br>Colaris, Joost; Erasmus MC, Department of Orthopaedics
**Primary Subject Heading**:	Surgery
Secondary Subject Heading:	Health economics, Emergency medicine
Keywords:	Adult orthopaedics < ORTHOPAEDIC & TRAUMA SURGERY, Trauma management < ORTHOPAEDIC & TRAUMA SURGERY, Hand & wrist < ORTHOPAEDIC & TRAUMA SURGERY, ACCIDENT & EMERGENCY MEDICINE

SCHOLARONE™
Manuscripts

I, the Submitting Author has the right to grant and does grant on behalf of all authors of the Work (as defined in the below author licence), an exclusive licence and/or a non-exclusive licence for contributions from authors who are: i) UK Crown employees; ii) where BMJ has agreed a CC-BY licence shall apply, and/or iii) in accordance with the terms applicable for US Federal Government officers or employees acting as part of their official duties; on a worldwide, perpetual, irrevocable, royalty-free basis to BMJ Publishing Group Ltd ("BMJ") its licensees and where the relevant Journal is co-owned by BMJ to the co-owners of the Journal, to publish the Work in this journal and any other BMJ products and to exploit all rights, as set out in our licence.

The Submitting Author accepts and understands that any supply made under these terms is made by BMJ to the Submitting Author unless you are acting as an employee on behalf of your employer or a postgraduate student of an affiliated institution which is paying any applicable article publishing charge ("APC") for Open Access articles. Where the Submitting Author wishes to make the Work available on an Open Access basis (and intends to pay the relevant APC), the terms of reuse of such Open Access shall be governed by a Creative Commons licence – details of these licences and which Creative Commons licence will apply to this Work are set out in our licence referred to above.

Other than as permitted in any relevant BMJ Author's Self Archiving Policies, I confirm this Work has not been accepted for publication elsewhere, is not being considered for publication elsewhere and does not duplicate material already published. I confirm all authors consent to publication of this Work and authorise the granting of this licence.

For peer review only

clinically SUspected ScaPhoid fracturE: treatment with supportive bandage or CasT? "Study protocol of a multicenter randomized controlled trial" (SUSPECT study)

Abigael Cohen[1], Max Reijman[1] , Gerald A Kraan[2], Nina M C Mathijssen[2], Marc A Koopmanschap[3], Jan A N Verhaar[1], Sander Mol[5], Joost W Colaris[1], on behalf of the SUSPECT study group

SUSPECT study group:

Peer van der Zwaal[6], Merel van Loon[7], Steven J Rhemrev[8], Daphne A van Rijssel[9], Mark R de Vries[10], Flip van Beek[11], Vanessa Brown[5], Tjebbe Hagenaars[12], Els H Jansen[13], Alexander P A Greeven[14], Lenneke C M Scholtens[15], Ruud L M Deijkers[16], Niels W L Schep[17], Akkie N Ringburg[18], Milko M M Bruijninckx[19], Kirsten F van Meerten[20], Willem-Maarten P F Bosman[21]

1 Department of Orthopaedic Surgery, Erasmus MC University Medical Center, Rotterdam, The Netherlands

2 Department of Orthopaedic Surgery, Reinier de Graaf Hospital, Delft, The Netherlands

3 Erasmus School of Health Policy and Management, Erasmus University Rotterdam, Rotterdam, The Netherlands

5 Department of Emergency Medicine, Franciscus Hospital, Rotterdam, The Netherlands

6 Department of Orthopaedic Surgery, Haaglanden Medical Centre, The Hague, The Netherlands

7 Department of Emergency Medicine, Haaglanden Medical Centre, The Hague, The Netherlands

8 Department of Surgery, Haaglanden Medical Centre, The Hague, The Netherlands,

9 Department of Emergency Medicine, Reinier de Graaf Hospital, Delft, The Netherlands

10 Department of Surgery, Reinier de Graaf Hospital, Delft, The Netherlands

11 Department of Surgery, Franciscus Hospital, Rotterdam, The Netherlands

For peer review only

For peer review only - http://bmjopen.bmj.com/site/about/guidelines.xhtml

12 Trauma Research Unit, Department of Surgery, Erasmus MC University Medical Center, Rotterdam, The Netherlands

13 Department of Emergency Medicine, Erasmus MC University Medical Center, Rotterdam, The Netherlands

14 Department of Surgery, Haga Teaching Hospital, The Hague, The Netherlands

15 Department of Emergency Medicine, Haga Teaching Hospital, The Hague, The Netherlands

16 Department of Orthopaedic Surgery, Haga Teaching Hospital, The Hague, The Netherlands

17 Department of Surgery, Maasstad Hospital, Rotterdam, The Netherlands

18 Department of Surgery, Ikazia Hospital, Rotterdam, The Netherlands

19 Department of Surgery, Ijsselland Hospital, Capelle aan den Ijssel, The Netherlands

20 Department of Emergency Medicine, Albert Schweitzer Hospital, Dordrecht, The Netherlands

21 Department of Surgery, Albert Schweitzer Hospital, Dordrecht, The Netherlands

Corresponding Author: Abigael Cohen, Telephone +31107043982, Email: a.cohen.1@erasmusmc.nl Department of Orthopedic Surgery, Erasmus MC University Medical Center, PO box 2040, 3000 CA Rotterdam, the Netherlands

Trial sponsor: Prof. dr. J.A.N. Verhaar, Email: j.verhaar@erasmusmc.nl Department of Orthopedic Surgery, Erasmus University MC Medical Center, PO box 2040, 3000 CA Rotterdam, the Netherlands

Authors' Contributions

MR and JC conceived the study idea and secured funding. AC, MR, GK, NM, MK, JV, SM and JC were involved with the initial study design. MR and MK were involved with the statistical analysis for the study. AC, GK, SM, JC, SUSPECT study group are involved with the implementation of the protocol

For peer review only

For peer review only - http://bmjopen.bmj.com/site/about/guidelines.xhtml

and the data collection. AC is conducting the study. All authors provided critical comments to the manuscript and approved the final version before submitting.

Funding statement

For this study, we received a grant from ZonMw, a Dutch organization for health research and care innovation and co-funding from CZ, a Dutch health insurance company. Both organizations were not involved in the study design.

Competing interests

None of the authors declared competing interests.

Trial registration

This study was prospectively registered at the Netherlands National Trial Register on February 28th, 2018, (www.trialregister.nl, NL6976)

Word count: 4022

Keywords: Scaphoid Bone, Fractures, Bone, Recovery of Function, Cost-Benefit Analysis, Wrist injuries.

For peer review only

ABSTRACT

Introduction: Some scaphoid fractures become visible on radiographs weeks after a trauma which makes normal radiographs directly after trauma unreliable. Untreated scaphoid fractures can lead to scaphoid non-union progressing to osteoarthritis. Therefore, the general treatment for patients with a clinically suspected scaphoid fracture and normal initial radiographs, is immobilization with below-elbow cast for 2 weeks. However, most of these patients are treated unnecessary because eventually

For peer review only - http://bmjopen.bmj.com/site/about/guidelines.xhtml

less than 10% of them are diagnosed with an occult scaphoid fracture. To reduce overtreatment and costs as a result of unnecessary cast treatment in patients with a clinically suspected scaphoid fracture and normal initial radiographs, we designed a study to compare treatment with below-elbow cast or supportive bandage. We hypothesize that the functional outcome after 3 months is similar in both groups, but with lower costs in the supportive bandage group.

Methods and analysis: The SUSPECT study is an open-labeled multicenter randomized controlled trial with non-inferiority design. A total of 180 adult patients with a clinically suspected scaphoid fracture and normal initial radiographs are randomized between two groups: 3 days supportive bandage or 14 days below-elbow cast. We aim to evaluate the functional outcome and cost-effectiveness of both treatments. The primary outcome is the difference in functional outcome after 3 months between both groups, assessed with the Quick Disability of the Arm, Shoulder and Hand score. Secondary outcomes include post-traumatic injuries, functional outcome, recovery of function, pain, patient satisfaction, quality of life and cost-effectiveness measured by medical consumption, absence from work or decreased productivity.

Ethics and dissemination: The medical ethics committee of the Erasmus MC Medical Centre, Rotterdam approved the study protocol (MEC-2017-504). We plan to present the results after completion of the study at (inter)national conferences and publish in general peer-reviewed journals.

Trial registration: www.trialregister.nl, NL6976, February 28th, 2018 registered.

STRENGTHS AND LIMITATIONS OF THIS STUDY

- This is the first multicenter randomized controlled trial to evaluate if treatment with supportive bandage results in not inferior functional outcome to below-elbow cast in patients with a clinically suspected scaphoid fracture.
- All patients are re-evaluated clinically and radiographically after one year.

For peer review only - http://bmjopen.bmj.com/site/about/guidelines.xhtml
For peer review only

- The cost-effectiveness of the treatment up to one year is studied.
- The main limitation of the study is that the study is not blinded for participants, physicians, and researchers.

INTRODUCTION

Up to 32% of all scaphoid fractures results in non-union, leading to progressive osteoarthritis,(1). While most fractures are visible on radiographs directly after trauma, not all scaphoid fractures can be diagnosed on the initial radiographs. To avoid untreated occult scaphoid fractures, all patients with a clinically suspected scaphoid fracture and normal initial radiographs are treated with a below-elbow cast until re-examination or further diagnostics,(2, 3). Finally, less than 10% of these patients appear to have a scaphoid fracture resulting in a large number of overtreatment, unnecessary absenteeism from work and increased health care costs,(4, 5).

Most occult scaphoid fractures can be diagnosed on repeated conventional radiography after 10-14 days (6). These occult fractures are in general non-displaced fractures of the waist or distal pole of the scaphoid, in which an intact periosteal envelop provides stability,(7, 8). Known risk factors for scaphoid non-union are proximal fracture location, fracture displacement and start of treatment more than 4 weeks after trauma (8-10). When adequate treatment for a scaphoid fracture is started within 4 weeks after the trauma, the non-union rate does not increase,(8). Therefore, supportive bandage and re-examination within 2 weeks can be a good alternative treatment for patients with a clinically suspected scaphoid fracture with normal radiographs. In 1988, one randomized study of 106 patients with a clinically suspected scaphoid fracture showed that treatment with supportive bandage compared with below-elbow cast in these patients decreased the immobilization time and absence of work without negative consequences on fracture healing. However, functional outcome and cost-effectiveness were not evaluated, and the follow-up period was not clearly stated,(8).

We designed a pragmatic, randomized controlled trial with 1 year follow up to evaluate the functional outcome and cost-effectiveness of treatment with a supportive bandage compared with

For peer review only - http://bmjopen.bmj.com/site/about/guidelines.xhtml
For peer review only

below-elbow-cast in patients with a clinically suspected scaphoid fracture and normal initial radiographs. We hypothesize that treatment with supportive bandage results in an not inferior functional outcome compared to below-elbow cast, with lower costs in the supportive bandage group.

Objectives

We designed a pragmatic, multicenter randomized controlled trial with a non-inferiority design with two groups. The primary objective of this study is to determine if treatment with supportive bandage compared with below-elbow cast in patients with clinically suspected scaphoid fracture and normal initial radiographs, results in not inferior functional outcome after 3 months, measured with the Quick Disabilities of the Arm, Shoulder and Hand (QDASH).

As secondary objectives, we assess whether treatment of patients with a clinically suspected scaphoid fracture with a bandage is cost-effective compared to cast. Cost-effectiveness is evaluated by medical consumption, absence from work or decreased productivity, and quality of life, measured in Quality-adjusted life years (QALYs).

Furthermore, we assess if supportive bandage compared to cast results in not inferior pain, patient satisfaction of the received treatment, functional outcome measured with the QDASH, recovery of function measured with physical examination and the Patient Rated Wrist/Hand Evaluation (PRWHE), and quality of life.

METHODS AND ANALYSIS

This manuscript is written according to the Consolidated Standards for Reporting Trials (CONSORT statement) and Standard Protocol Items: Recommendations for Interventional Trials (SPIRIT guidelines),(11, 12).

Patient and Public Involvement statement

For peer review only - http://bmjopen.bmj.com/site/about/guidelines.xhtml

151 In the absence of a patient association, we formed a panel of 3 patients with a (suspected) scaphoid fracture to think along with, and comment on our study. This panel of patients analyzed the study (aim, burden for patient, willingness to participate), patient information sheet and how the future results should be reported to the patients. To improve our study, we evaluate the study procedures and the patient burden with participating patients during the recruitment period. After termination of the study, we present our study results to all participating patients.

Study design

The SUSPECT study is performed in 9 hospitals in the Netherlands. All departments involved in the treatment of patients with a clinically suspected scaphoid fracture in each participating hospital participate in the study.

All patients who present with a clinically suspected scaphoid fractureafter trauma at the emergency department (ED), who match the in- and exclusion criteria, are informed about the study by their treating physician. These patients receive the participant information sheet and are invited to ask questions about the study. As direct start of treatment is necessary, patients have to decide on study participation after the information is provided at the ED. Written informed consent is obtained from the patient prior to inclusion (Supplementary appendix 1). All included patients are randomized at the ED and allocated to treatment with supportive bandage or below-elbow cast.

Patients revisit the outpatient clinic after 14 days and 1 year. After 14 days, the researcher re-examines the patient first. Secondly, radiographs with scaphoid specific views are obtained (without cast or bandage around the wrist) according to the hospital protocol. Lastly, the physician re-examines the patient and determines the diagnosis, treatment and follow-up according to their hospital protocol without interference of the researcher . When a scaphoid fracture is diagnosed by the physician, patients are treated for a scaphoid fracture according hospital protocol with either

For peer review only - http://bmjopen.bmj.com/site/about/guidelines.xhtml

cast or surgery. After 1 year, the researcher re-examines the patients and wrist radiographs are made.

During the follow up period, patients receive questionnaires by email after inclusion, 2 weeks, 6 weeks, 3 months, 6 months, 9 months and 1 year of follow up.

Due to the Dutch privacy law, it is not allowed to screen eligible patients at the ED to detect how many eligible patients are not participating in our study.

Study population

All adult patients who visit the ED of the participating hospitals, within 48 hours after trauma with a clinically suspected scaphoid fracture without a fracture on the radiograph, are invited to participate in the study.

Inclusion criteria

- Age of 18 years or older
- Trauma maximum 48 hours before
- Anatomical snuff box (ASB) tenderness or scaphoid tubercle (ST) tenderness
- No scaphoid fracture reported by the radiologist or treating physician on the initial radiographs with scaphoid specific views (minimally 3 views)

Exclusion criteria

- Concomitant injury of the ipsilateral extremity that needs treatment with movement restriction by cast or supportive bandage.
- Inability to complete study forms due to insufficient command of the Dutch language.

For peer review only - http://bmjopen.bmj.com/site/about/guidelines.xhtml
For peer review only

- A scaphoid fracture diagnosed on the initial radiographs by a supervising radiologist in retrospect.

Randomization

After written informed consent is obtained, patients are central randomized through computer-based variable block randomization (2, 4, 6 blocks) by Castor Electronic Data Capture (EDC) to enable allocation concealment,(13). The allocation ratio is 1:1 and randomization is stratified per hospital due to potential differences in applied cast and the number of scaphoid specific views per hospital (Figure 1).

Because of practical reasons, there is no blinding of the treatment group for the physician, patient and researcher, but assessment of the radiographs is blinded. We will perform statistical analysis blinded for allocated treatment. .

Interventions

Intervention group (supportive bandage)

Patients in the intervention group receive a below-elbow supportive bandage for 3 days at the ED. After 3 days the patients are allowed to remove the bandage and move the wrist driven by the amount of pain. When patients experience too much pain despite adequate analgesia, an additional appointment is made at the outpatient clinic to apply a below-elbow cast for analgesic purposes. A similar below-elbow cast, to the control group, is applied conform hospital protocol. The patients that cross-over to cast remain in the study.

Control group (below-elbow cast)

For peer review only - http://bmjopen.bmj.com/site/about/guidelines.xhtml

The usual care in the Netherlands for patients with clinically suspected scaphoid fracture at the ED is below-elbow cast and re-examination after 14 days (Figure 2),(2). The applied below-elbow cast type is according to hospital protocol and can be circular or splint, with or without the thumb included.

Outcome

We collect the following baseline variables; demographic data (e.g. age, gender, height, weight, hand dominance), details about the trauma (e.g. fall onto an outstretched hand (FOOSH), high-energy trauma, low-energy trauma, sports injury) and comorbidities such as smoking status, previous arm injuries and general medical history.

Primary outcome

The primary outcome is the difference in functional outcome between the intervention and control group measured with the QDASH after 3 months of follow-up. We selected the QDASH as the primary outcome, as it takes the function of both hands into account. With this information, we can reflect if the wrist problem results in an impaired functional outcome in daily life.

Secondary outcomes

- Functional outcome measured with the QDASH after inclusion and at 2 weeks, 6 weeks and 1 year of follow up (Table 1).
- Recovery of function evaluated with PRWHE after inclusion and at 2 weeks, 6 weeks, 3 months and 1 year of follow up.
- The amount of pain scored with the Visual Analogue Scale (VAS) score at rest and during movement after inclusion and at 2 weeks, 6 weeks, 3 months and 1 year of follow up.
- Patient satisfaction of the treatment is measured after 2 weeks and 3 months of follow up.

For peer review only - http://bmjopen.bmj.com/site/about/guidelines.xhtml

- Quality of life assessed by the 5-level EuroQol-5D (EQ-5D-5L) after inclusion and at 2 weeks, 6 weeks, 3 months and 1 year of follow up. When the patient reports medical consumption or decreased productivity due the wrist problems after 6 or 9 months of follow up, the EQ-5D-5L is sent to the patients as well.
- To assess the costs and cost-effectiveness of both interventions, we collect information on diagnostic (additional imaging) and therapeutic hospital procedures using the electronic patient status and questionnaires. We use iMTA Medical Consumption Questionnaire (iMCQ) and iMTA Productivity Cost Questionnaire (iPCQ) after 6 weeks, 3 months, 6 months, 9 months and 1 year of follow up (Table 1).
- Physical examination is performed at inclusion, after 2 weeks and 1 year of follow up. At inclusion, the physical examination is performed by the treating physician. They will register if the patient has tenderness ASB, tenderness ST, compression pain thumb, radial sided wrist pain with resisted supination and radial sided wrist pain with ulnar deviation. A previous study showed that these clinical tests have the highest sensitivity for a scaphoid fracture,(14). After 2 weeks and 1 year, the function of the wrist is assessed by the researcher. The physical examination includes the abovementioned clinical tests and swelling or hematoma on the radial side of the wrist. Range of motion of the wrist (palmar flexion, dorsal flexion, ulnar deviation, radial deviation, supination, and pronation) and finger-to-palm distance are measured with a goniometer. The finger-to-palm distance is the distance from the tip of the finger to the distal palmar crease, when the fingers are in maximal active flexion,(15). We assess opposition of the thumb with the Kapandji score,(16). The Kapandji score ranges from 0 – 10, whereas 0 indicates no opposition and 10 maximal opposition. Grip strength is measured with a hand dynamometer (Jamar hand dynamometer), and is defined as the maximum grip strength after 3 attempts. Range of motion and grip strength of both wrists are examined. One year after inclusion, we additional test the scapholunate (SL) ligament by identifying tenderness over the dorsal SL interval, performing the finger

For peer review only

For peer review only - http://bmjopen.bmj.com/site/about/guidelines.xhtml

**Table 1** Description of patient-rated outcome measures and examinations per follow up moment.							
	Baseline	2 weeks	6 weeks	3 months	6 months	9 months	1 year
**Radiograph**	Scaphoid	Scaphoid					Wrist
**Physical examination**	x	x					x
**PROM**	QDASH PRWHE	QDASH PRWHE	QDASH PRWHE	QDASH PRWHE	iMCQ iPCQ	iMCQ iPCQ	QDASH PRWHE

271 extension test, and the Watson test (Table 2). The finger extension test is positive when
272 resisted extension of the fingers with the wrist in flexion is painful,(17). To perform the
273 Watson test, the patients' wrist is in dorsal flexion and ulnar deviation, with the researchers
274 thumb on ST and fingers wrapped around the distal radius. Then, the researcher moves the
275 patients' wrist radially and in palmar flexion. The Watson test is positive if a painful clunk is
276 noticed,(17, 18).

- 277 Radiographic re-examination is performed after 2 weeks and 1 year of follow up. After 2
278 weeks, the radiographs include scaphoid specific views (minimally 3 views) to identify a
279 scaphoid fracture. After 1 year, a posterior-anterior and lateral wrist radiographs are made
280 to determine the presence of any post-traumatic injuries (e.g. scaphoid non-union, SL
281 dissociation). The radiographs are evaluated by two independent assessors.
- 282 Detailed information about the received treatment is reported. We address the number of
283 patients that cross over from their allocated treatment , days of cast or supportive bandage
284 before the outpatient department appointment and applied treatment after the outpatient
285 department appointment (cast or surgery).
- 286 (Serious) adverse events are noted, such as scaphoid fracture and scaphoid nonunion.
287 (Detailed information can be found at safety considerations).
- 288 Other diagnosis are reported such as other carpal fractures or ligament injuries.

289

290

For peer review only - http://bmjopen.bmj.com/site/about/guidelines.xhtml

	VAS<br>EQ-5D-5L	VAS<br>Patient satisfaction<br>EQ-5D-5L	VAS<br>EQ-5D-5L<br>iMCQ<br>iPCQ	VAS<br>Patient satisfaction<br>EQ-5D-5L<br>iMCQ<br>iPCQ	(EQ-5D-5L)	(EQ-5D-5L)	VAS<br>EQ-5D-5L<br>iMCQ<br>iPCQ

PROM, Patient-Rated Outcome Measure; QDASH, *Quick Disability of the Arm, Shoulder and Hand; PRWHE, Patient Rated Wrist Hand Evaluation; VAS, Visual Analog Score; EQ-5D-5L, 5-level EuroQol-5D; iMCQ, iMTA Medical Consumption Questionnaire; iPCQ, iMTA Productivity Cost Questionnaire.*

Table 2 Physical examination per follow up moment

	Baseline	2 weeks	1 year
Swelling radial side wrist		x	x
Tenderness ST	x	x	x
Compression pain thumb	x	x	x
Painful resisted supination	x	x	x
Painful UD	x	x	x
SL ligament			x
Kapandji score		x	x
Range of motion		x	x
Finger-to-palm distance		x	x
Grip strength		x	x

UD, Ulnar deviation; ASB, *Anatomical snuff box; ST, Scaphoid Tubercle; SL, Scapholunate ligament examinations consist of tenderness SL interval, Finger extension test and Watson test. Range of motion include palmar flexion, dorsal flexion, ulnar deviation, radial deviation, supination and pronation.*

Instruments

For peer review only - http://bmjopen.bmj.com/site/about/guidelines.xhtml

Functional outcome is measured with the mandatory module of the QDASH. The QDASH is the short version of the DASH and consists of 11 questions about symptoms and physical function of both arms during the last week. Each question can be scored from 1 - 5. A high score indicates severe disability of the upper extremity. The Dutch QDASH has shown good validity, reliability and responsiveness for comparable patients,(19, 20).

The PRWHE questionnaire evaluates the painful wrist during the last week. The questionnaire consists of three subscales about pain, function, and cosmetics. The cosmetic field is not included in the score. The pain and function subscales consist each of 15 questions that can be scored from 0-10. A high PRWHE score indicates severe pain and restricted function of the painful wrist. The Dutch PRWHE has shown good validity, reliability and responsiveness for comparable patients,(20) .

Pain scores at rest and during movement are examined with the eleven point Visual Analogue Scale (VAS) for pain. A high VAS score indicates severe pain. The VAS for pain is widely used in research with comparable patients,(21).

Patient satisfaction is assessed by the questions 'how satisfied are you with the treatment', 'did you prefer the other treatment?', and 'if yes, why?'. The first question can be scored from 0-10. A high score indicates satisfaction.

Quality of life is measured with the EQ-5D-5L. This questionnaire consists of five questions and a visual analogue scale. The questions are about mobility, self-care, usual activities, pain or discomfort, and anxiety or depression. Each question can be scored as: no problem, slight problem, moderate problem, severe problem, and extreme problem. Health is reported on a visual analogue scale from 0-100. A high score indicates better health. The EQ-5D-5L is recommended for the assessment of quality of life in trauma patients, especially for economic assessments,(22).

To assess the costs and cost-effectiveness of the intervention with the usual care we include the Medical Consumption Questionnaire (iMCQ) and Productivity Cost Questionnaire (iPCQ). The iMCQ measures the total medical consumption by measuring the use of diagnostics, consultations, emergency department visits, physical therapy, medication, and aids. The iPCQ measures

For peer review only - http://bmjopen.bmj.com/site/about/guidelines.xhtml
For peer review only

331 productivity costs. This questionnaire evaluates costs of absence of work and decreased productivity at paid or unpaid work. The iMCQ and iPCQ s are short generic measurement instruments,(23).

334 **Sample size**

335 Our hypothesis is that patients with a clinically suspected scaphoid fracture with normal initial radiographs treated with a bandage have not inferior functional outcome after 3 months compared with cast, but with lower costs. The power calculation is based on the proof of non-inferiority. We took 50% of the margin of clinical minimal important difference of QDASH,(24). This resulted in a margin of non-inferiority of 7.50 points on the QDASH. The standard deviation of QDASH after 3 months has been reported to be 14,(25). We used a power of 90% and a one-sided alpha of 0.025. To detect non-inferiority of bandage compared to cast 148 patients are needed (74 patients per group). Accounting for a 15% lost to follow-up, a total of 180 patients are required. When the lost to follow-up or change of allocated treatment is higher than 15%, we will include more patients to have 74 patients per group to answer our primary research question. When the patient recruitment rate at the ED is behind our expectations, we invite more hospitals to participate.

347 **Data collection**

348 We will collect data by electronic data capture with Castor EDC,(13). Paper based case report forms are used at the ED and the outpatient department. The researcher inserts this data in Castor EDC. Questionnaires are sent directly to the patients through email via Castor EDC. In case an email address is not available, a paper case report form is sent to the patient. When the patient does not respond, we will contact the patient by telephone.

353 We screen the electronic patient status to collect information about (serious) adverse events, hospital visits, additional imaging and treatment for the wrist injury during the follow-up period of the study.

For peer review only - http://bmjopen.bmj.com/site/about/guidelines.xhtml

Data analysis

We will perform all analyses blinded for treatment allocation. The distribution analysis of baseline variables is tested by the Shapiro-Wilk test. For normally distributed variables, parametric tests are used. For those variables that are not normally distributed, non-parametric tests are used. We report the proportion of diagnosis and adverse events per allocated treatment group.

All outcomes are analyzed ‘by intention to treat analysis’ with a linear mixed model with specified fixed and random effects. The covariance structure is unstructured. Restricted maximum likelihood will be used to estimate parameters. The assumptions of our model (linearity, homoscedasticity and normal distribution of the error terms) are tested. Should any of these assumptions seriously fail, variable transformations are used.

For our primary outcome, QDASH at 3 months, and secondary outcomes PRWHE, VAS score, EQ5D-5L at measurement time, and an interaction term of measurement time by treatment. Hospital is a random intercept and to analyze repeated measurements, we add a random intercept for patients (nested with hospital). Baseline, and 2 weeks, 6 weeks, and 3 months follow up are included in our model as reapeated measures. We perform post-hoc analyses to compare estimated means at 3 months. All outcomes are assessed for non-inferiority, expect cost-effectiveness which is assessed for superiority. A margin of non-inferiority of 7.50 is used for our primary outcome in the analyses. If baseline imbalances are present between the two groups, we add these as fixed effects if the effected estimate changes with more than 10%. Potential confounders we test for are: age, gender, Body Mass Index, presence of comorbidities that influence function of the arms, previous fracture of hand or wrist, diabetes, smoking status, dominant side affected, trauma mechanism(High energetic trauma, Low energetic trauma, sport, traffic accident, assault) and employment status (in school, unemployed, retired, entrepreneur, employee, occupational disabled). We perform additional as-treated analysis to analyze outcomes between patients that received only supportive bandage until the outpatient department appointment after 2 weeks and patients that received cast in the first 2

For peer review only - http://bmjopen.bmj.com/site/about/guidelines.xhtml

weeks (received cast at the ED or crossover from bandage). Patients are included in our mixed models when they responded to the outcome questionnaire at either 2 weeks ,6 weeks or 3 months follow up. When patients respond to neither of these measurements periods, the patient will be scored as loss to follow up. When one or more (but not all) of the measurements are missing, a sensitivity analysis will be performed to clarify if the missing data is at random.

Furthermore, we assess the progress of the QDASH, PRWHE, VAS, EQ5D-5L and physical examination during the 1 year follow-up. The random and fixed effects will remain similar, only all follow up moments will be included in the repeated measurements.

Cost-effectiveness analysis (CEA)

General considerations

We assess the cost-effectiveness of 3 days supportive bandage versus 14 days below-elbow cast over a period of 12 months. To answer this question, we perform a cost-utility analysis to estimate the incremental cost per QALY gained, to be able to compare our study with other studies in musculoskeletal disorders and in other disease categories. We impute missing data by multiple imputation, using the pattern mixture model. Using non-parametric bootstrapping (randomly drawing 5000 observations with replacement from the patient sample), the degree of uncertainty for costs and health effects and the cost-utility ratio are depicted in a cost-effectiveness plane. In addition, an acceptability curve is drawn, which indicates the probability that the intervention studied has lower incremental costs per QALY gained than various thresholds (dependent on disease severity) for the maximum willingness to pay for an extra QALY.

Cost analysis

The economic analysis is based on the societal perspective, and on the healthcare perspective in which the direct medical and productivity costs between the groups are compared. To measure the direct costs, an inventory of patients total medical consumption is made using the electronic patient

For peer review only

For peer review only - http://bmjopen.bmj.com/site/about/guidelines.xhtml

status and the iMCQ. The use of diagnostics (additional radiographs, CT, MRI, ultrasound or bone scintigraphy), consultations, emergency department visits, physical therapy, medication and aids are evaluated. Productivity costs are measured with the iPCQ. Costs of absence of work and decreased productivity at a paid job or at unpaid work are evaluated. The friction cost method is used to calculate the productivity costs according to the recent Dutch guidelines. The costs per unit of medical consumption is estimated, using information from the most recent Dutch Manual for economic evaluation of health care. Costs are reported for the year 2019.

Cost-utility analysis

Patient outcomes relevant for the CEA are the number of QALYs during 1 year. Patient scores on the EQ 5D-5 level version are converted into utility values, using the valuation algorithm of the Dutch general population.

Budget impact analysis (BIA)

Guidelines of the ISPOR Task Force are used for the BIA. Relevant features and tariffs of the Dutch health care system, anticipated uptake of the new intervention as well as usual care are taken into account. The size and the eligibility of the population, cost of diagnostics, treatment modalities and changes expected in condition-related costs are considered in the BIA. Sensitivity analyses are performed using divergent scenarios from the viewpoint of the decision-makers.

The budget impact per year of implementing the new intervention is estimated. All elements of medical costs for the new intervention as well as for usual care that are paid by third-party payers are considered and calculated.

ETHICS AND DISSEMINATION

For peer review only - http://bmjopen.bmj.com/site/about/guidelines.xhtml

Safety considerations

The study compares treatment of below-elbow cast (standard treatment) with supportive bandage (intervention). Since the study is labeled as low risk, a data safety monitoring board is not required. The study is monitored at least once a year and the progress of the trial is reported once a year to the accredited medical review ethics committee (MEC). Adverse events are recorded by the researcher during follow up. A serious adverse event is reported (www.toetsingonline.nl) within a maximum of 15 days after the researcher is notified.

Adverse events are defined as:

- Cast related problems
- Bandage related problems
- Loss of function
- Complex regional pain syndrome
- Persisting pain after 6 weeks
- Conservative treated scaphoid non-union

Serious adverse events related to our study are defined as:

- Surgically treated scaphoid fracture
- Compartment syndrome
- Scaphoid non-union (scaphoid fracture without signs of healing after minimally 12 weeks).

Because of the multicenter study design, clinical trial site agreements are obtained between the initiating and the participating hospitals. These agreements include liability and insurance aspects. The patient information sheet reports on participants insurance and potential complications as a result of participation in the study. Withdrawal from the study is always possible for included patients without any consequences.

For peer review only - http://bmjopen.bmj.com/site/about/guidelines.xhtml

For peer review only

Ethics

The study is approved by the MEC of the Erasmus Medical Centrum, Rotterdam, the Netherlands (MEC-2017-504). All amendments are notified to the MEC and changes to the study are made after a favorable opinion of the MEC.

The study is conducted according to the principles of the Declaration of Helsinki (64th version, date: October 2013) and in accordance with the Medical Research Involving Human Subjects Act (WMO) and other guidelines, regulations, and acts.

The study is registered in the Dutch Clinical trials Registry (www.trialregister.nl, trial NL6976).

Participating patients and physicians are not paid for participation in the study.

Data management

Personal data of the participating patients that we collect during the study, is changed to a study number. This study number is used for all study documentation, study reports and publications.

Data is collected with Castor EDC. Paper case report forms are entered in Castor EDC by the researcher. The paper case report forms are securely filed in the hospital. All data is stored for 15 years. The final trial dataset is accessible for the research team.

Dissemination

After completion of the study, we plan to present the results at (inter)national congresses and submit the manuscript to general peer-review journals.

Authorship is according to the International Committee of Medical Journal Editors guidelines and a SUSPECT study group is made.

For peer review only - http://bmjopen.bmj.com/site/about/guidelines.xhtml

For peer review only

REFERENCES

1. Inoue G, Sakuma M. The natural history of scaphoid non-union. Radiographical and clinical analysis in 102 cases. ARCH ORTHOP TRAUMA SURG. 1996;115(1):1-4.
2. Mallee WH, Veltman ES, Doornberg JN, Blankevoort L, van Dijk CN, Goslings JC. [Variations in management of suspected scaphoid fractures]
Variatie in beleid bij vermoeden op scafoidfractuur. Ned Tijdschr Geneeskd. 2012;156(28):A4514.
3. Borel C, Larbi A, Delclaux S, Lapegue F, Chiavassa-Gandois H, Sans N, et al. Diagnostic value of cone beam computed tomography (CBCT) in occult scaphoid and wrist fractures. Eur J Radiol. 2017;97:59-64.
4. Bergh TH, Steen K, Lindau T, Soldal LA, Bernardshaw SV, Lunde L, et al. Costs analysis and comparison of usefulness of acute MRI and 2 weeks of cast immobilization for clinically suspected scaphoid fractures. Acta Orthop. 2015;86(3):303-9.
5. de Zwart AD, Beeres FJ, Rhemrev SJ, Bartlema K, Schipper IB. Comparison of MRI, CT and bone scintigraphy for suspected scaphoid fractures. Eur J Trauma Emerg Surg. 2016;42(6):725-31.
6. Gemme S, Tubbs R. What physical examination findings and diagnostic imaging modalities are most useful in the diagnosis of scaphoid fractures? Ann Emerg Med. 2015;65(3):308-9.
7. DaCruz DJ, Bodiwala GG, Finlay DB. The suspected fracture of the scaphoid: a rational approach to diagnosis. Injury. 1988;19(3):149-52.
8. Sjølin SU, Andersen JC. Clinical fracture of the carpal scaphoid—supportive bandage or plaster cast immobilization? The Journal of Hand Surgery: British & European Volume. 1988;13(1):75-6.
9. Langhoff O, Andersen JL. Consequences of late immobilization of scaphoid fractures. J Hand Surg Br. 1988;13(1):77-9.
10. Leslie IJ, Dickson RA. The fractured carpal scaphoid. Natural history and factors influencing outcome. J Bone Joint Surg Br. 1981;63-B(2):225-30.
11. Schulz KF, Altman DG, Moher D. CONSORT 2010 Statement: updated guidelines for reporting parallel group randomised trials. Bmj. 2010;340:c332.
12. Chan AW, Tetzlaff JM, Gotzsche PC, Altman DG, Mann H, Berlin JA, et al. SPIRIT 2013 explanation and elaboration: guidance for protocols of clinical trials. Bmj. 2013;346:e7586.
13. Ciwit BV. Castor Electronic Data Capture. Amsterdam2016.
14. Mallee WH, Henny EP, van Dijk CN, Kamminga SP, van Enst WA, Kloen P. Clinical diagnostic evaluation for scaphoid fractures: a systematic review and meta-analysis. J Hand Surg Am. 2014;39(9):1683-91 e2.
15. Torok KS, Baker NA, Lucas M, Domsic RT, Boudreau R, Medsger TA, Jr. Reliability and validity of the delta finger-to-palm (FTP), a new measure of finger range of motion in systemic sclerosis. Clin Exp Rheumatol. 2010;28(2 Suppl 58):S28-36.
16. Kapandji A. [Clinical test of apposition and counter-apposition of the thumb]
Cotation clinique de l'opposition et de la contre-opposition du pouce. Ann Chir Main. 1986;5(1):67-73.
17. Skirven T. Clinical examination of the wrist. Journal of Hand Therapy. 1996;9(2):96-107.
18. Walsh JJ, Berger RA, Cooney WP. Current status of scapholunate interosseous ligament injuries. JAAOS-Journal of the American Academy of Orthopaedic Surgeons. 2002;10(1):32-42.
19. Tsang P, Walton D, Grewal R, MacDermid J. Validation of the QuickDASH and DASH in Patients With Distal Radius Fractures Through Agreement Analysis. Arch Phys Med Rehabil. 2017;98(6):1217-22 e1.
20. Kleinlugtenbelt YV, Krol RG, Bhandari M, Goslings JC, Poolman RW, Scholtes VAB. Are the patient-rated wrist evaluation (PRWE) and the disabilities of the arm, shoulder and hand (DASH) questionnaire used in distal radial fractures truly valid and reliable? Bone Joint Res. 2018;7(1):36-45.

For peer review only - http://bmjopen.bmj.com/site/about/guidelines.xhtml

21. Badalamente M, Coffelt L, Elfar J, Gaston G, Hammert W, Huang J, et al. Measurement scales in clinical research of the upper extremity, part 1: general principles, measures of general health, pain, and patient satisfaction. J Hand Surg Am. 2013;38(2):401-6; quiz 6.
22. Hung M-C, Lu W-S, Chen S-S, Hou W-H, Hsieh C-L, Wang J-D. Validation of the EQ-5D in Patients with Traumatic Limb Injury. Journal of Occupational Rehabilitation. 2015;25(2):387-93.
23. Bouwmans C, Krol M, Severens H, Koopmanschap M, Brouwer W, Hakkaart-van Roijen L. The iMTA Productivity Cost Questionnaire: A Standardized Instrument for Measuring and Valuing Health-Related Productivity Losses. Value Health. 2015;18(6):753-8.
24. Franchignoni F, Vercelli S, Giordano A, Sartorio F, Bravini E, Ferriero G. Minimal clinically important difference of the disabilities of the arm, shoulder and hand outcome measure (DASH) and its shortened version (QuickDASH). J Orthop Sports Phys Ther. 2014;44(1):30-9.
25. Buijze GA, Goslings JC, Rhemrev SJ, Weening AA, Van Dijkman B, Doornberg JN, et al. Cast immobilization with and without immobilization of the thumb for nondisplaced and minimally displaced scaphoid waist fractures: a multicenter, randomized, controlled trial. J Hand Surg Am. 2014;39(4):621-7.

Author's contributions:

MR and JC conceived the study idea and secured funding. AC, MR, GK, NM, MK, JV, SM and JC were involved with the initial study design. MR and MK were involved with the statistical analysis for the study. AC, GK, SM, JC, SUSPECT study group are involved with the implementation of the protocol and the data collection. AC is conducting the study. All authors provided critical comments to the manuscript and approved the final version before submitting.

Funding

For this study, we received a grant from ZonMw, a Dutch organization for health research and care innovation and co-funding from CZ, a Dutch health insurance company. Both organizations were not involved in the study design.

Trial status

We are currently recruiting patients for the study. The date of first enrollment was 7 june 2018.

Competing interests statement

The authors declare to have no competing interests.

For peer review only - http://bmjopen.bmj.com/site/about/guidelines.xhtml

Legend:

Figure 1 SUSPECT study flow chart. PROMs; Patient rated outcome measures

Figure 2 Treatment of intervention and control group

For peer review only

For peer review only - http://bmjopen.bmj.com/site/about/guidelines.xhtml

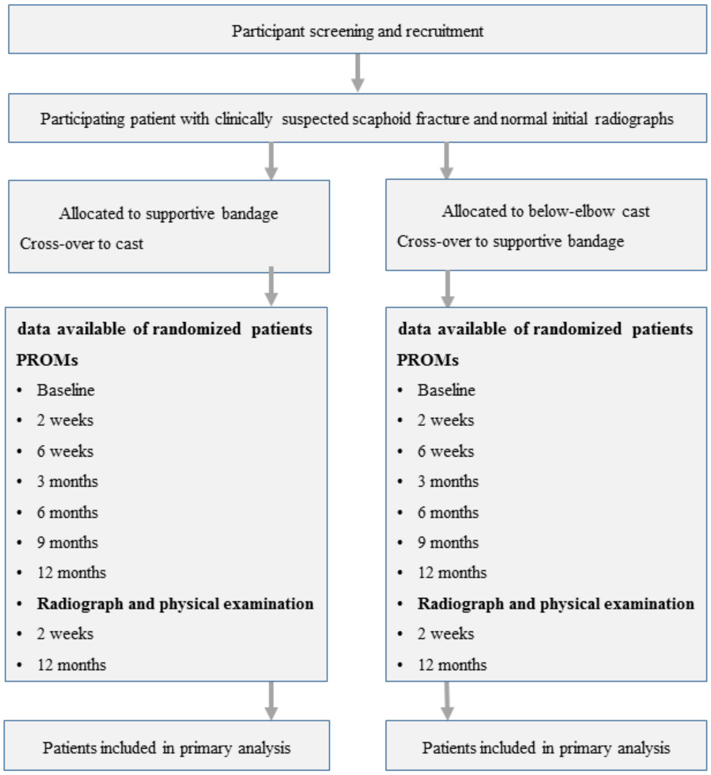

Figure 1 SUSPECT study flow chart. PROMs; Patient rated outcome measures

190x275mm (96 x 96 DPI)

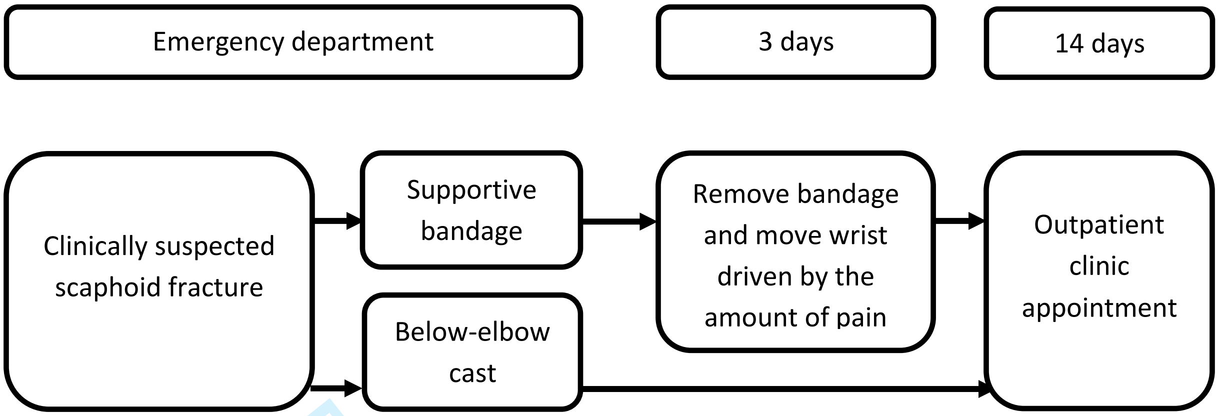

For peer review only

Polspijn zonder afwijkingen op de röntgenfoto's

Proefpersoneninformatie voor deelname aan medisch-wetenschappelijk onderzoek

Titel van het onderzoek

Polspijn zonder afwijkingen op de röntgenfoto's

Geachte heer/mevrouw,

U heeft de spoedeisende hulp bezocht vanwege pijn in uw pols. Wij willen u vragen of u wilt deelnemen aan een medisch-wetenschappelijk onderzoek. U beslist zelf of u wilt meedoen. Voordat u de beslissing neemt, is het belangrijk om meer te weten over het onderzoek. Lees daarom deze informatiebrief rustig door. Bespreek het met partner, vrienden of familie. Ook is er een onafhankelijke persoon, die veel weet van het onderzoek. Lees ook de Algemene brochure. Daar staat veel algemene informatie over medisch-wetenschappelijk onderzoek in. Heeft u na het lezen van de informatie nog vragen? Dan kunt u terecht bij de arts-onderzoeker. In bijlage C vindt u haar contactgegevens.

1. Algemene informatie

Dit onderzoek is opgezet door het Erasmus MC en het Reinier de Graaf Gasthuis en wordt gedaan door artsen in verschillende ziekenhuizen.

Voor dit onderzoek zijn 180 proefpersonen nodig die polspijn hebben na een ongeval.

De medisch-ethische toetsingscommissie van het Erasmus MC heeft dit onderzoek goedgekeurd. Algemene informatie over de toetsing van onderzoek vindt u in de brochure 'Medisch-wetenschappelijk onderzoek'. Deze brochure is ook online beschikbaar. Zie hiervoor de volgende link: file:///H:/Downloads/medisch-wetenschappelijk+onderzoek+algemene+informatie+voor+de+proefpersoon.pdf

2. Wat is het doel van het onderzoek?

Polspijn na een ongeval kan veroorzaakt worden door een kneuzing of een botbreuk. Om het onderscheid te kunnen maken tussen een kneuzing of een botbreuk zijn er bij u een aantal röntgenfoto's gemaakt. Er zijn op dit moment op uw röntgenfoto's geen botbreuken te zien zijn, maar een breuk van het scheepvormige botje kan soms pas te zien zijn na 2 weken. Daarom worden alle patiënten met verdenking op een botbreuk van het scheepvormige botje behandeld met onderarmgips en na 2 weken teruggezien. Aangezien uiteindelijk maar 1 van de 10 patiënten een

For peer review only - http://bmjopen.bmj.com/site/about/guidelines.xhtml

botbreuk heeft worden dus veel patiënten onnodig behandeld met gips. Met dit onderzoek willen we daarom kijken of de behandeling niet even goed is met een drukverband gedurende 3 dagen in plaats van een onderarmgips voor 2 weken.

3. Wat wordt er onderzocht?

In dit onderzoek wordt een drukverband vergeleken met een onderarm gips. Om dit te kunnen beoordelen willen wij u vragen om gedurende de studie een aantal vragenlijsten in te vullen. Voor het onderzoek is het belangrijk dat u dit zo objectief mogelijk invult. Daarnaast zullen wij de functie van de pols beoordelen.

4. Wat meedoen inhoudt?

Als u meedoet, duurt dat totaal ongeveer 1 jaar voor u. De studie gaat in totaal vier jaar duren.

Behandeling

Als u besluit om mee te doen zal er worden geloot tussen de behandeling met onderarmgips gedurende 2 weken en de behandeling met drukverband gedurende 3 dagen. Uw behandelend arts en de onderzoekers hebben geen invloed op de uitslag van de loting.

Bezoek en metingen

Gedurende de studie zullen wij op vijf momenten vragen om vragenlijsten in te vullen (ongeveer 15 minuten per meetmoment). Via deze vragenlijsten zullen wij het dagelijks functioneren van de arm, kwaliteit van leven en de kosten met betrekking tot medische zorg en arbeidsverzuim evalueren. Daarnaast zullen wij tijdens twee bezoeken aan het ziekenhuis de onderarmen onderzoeken op bewegelijkheid en kracht. Ook zullen er röntgenfoto's worden gemaakt om de botten van de pols goed in beeld te brengen. Op het moment dat er tijdens deze controles een breuk wordt ontdekt op de röntgenfoto's zal de behandeling zich richten op herstel van deze breuk. In bijlage B vindt u een overzicht van alles wat wordt gepland als u deelneemt.

Anders dan gebruikelijke zorg

Als u aan het onderzoek deelneemt dan krijgt u afhankelijk van de loting een drukverband. Dit is momenteel niet de standaardbehandeling. Indien u inloot voor de groep met het onderarmgips, dan krijgt u gewoon de behandeling die u ook zou krijgen als u niet meedeed aan dit onderzoek.
Na twee weken wordt er nogmaals een röntgenfoto gemaakt. Dit maakt onderdeel uit van de standaardbehandeling en zal dus ook gebeuren als u niet meedoet aan dit onderzoek. Mocht er dan toch een botbreuk op de röntgenfoto's te zien zijn, wordt u alsnog behandeld voor de botbreuk. Eerder onderzoek heeft aangetoond dat latere behandeling van een botbreuk van het scheepvormige botje geen nadelige gevolgen heeft voor het uiteindelijk herstel. Indien u deelneemt aan het onderzoek krijgt u een extra poliklinische controle na 1 jaar. Deze extra poliklinische controle

For peer review only - http://bmjopen.bmj.com/site/about/guidelines.xhtml

For peer review only

is voor beide groepen hetzelfde. Hier zullen voor u geen extra kosten aan verbonden zijn. De reguliere behandeling bestaat uit een onderarmgips en poliklinische controle met röntgenfoto's twee weken na het ongeval (zie bijlage B).

5. Wat wordt er van u verwacht?

Om het onderzoek goed te laten verlopen is het belangrijk dat u zich aan de volgende afspraken houdt.

De afspraken zijn dat u:

- de gips of verband behandeling volgt volgens de uitleg.
- niet ook nog aan een ander medisch-wetenschappelijk onderzoek meedoet, die invloed heeft op de huidige studie.
- afspraken voor bezoeken nakomt.

Het is belangrijk dat u contact opneemt met de onderzoeker:

- als u in een ziekenhuis wordt opgenomen of behandeld.
- als u plotseling gezondheidsklachten krijgt.
- als u niet meer wilt meedoen aan het onderzoek.
- als uw contactgegevens wijzigen.

6. Welke bijwerkingen en risico's kunt u verwachten?

De behandeling van polspijn zonder afwijkingen op de röntgenfoto's brengt niet veel bijwerkingen of risico's met zich mee. De behandeling met gips zal meer kans geven op drukplekken van het gips en stijfheid van de pols. De behandeling met drukverband geeft mogelijk een groter risico op trager herstel van een mogelijk botbreuk van het scheepvormig botje alhoewel eerdere studies dit ontkrachten.

7. Wat zijn mogelijke voor- en nadelen van deelname aan dit onderzoek?

U heeft zelf geen voordeel van deelname aan dit onderzoek. Voor de toekomst kan het onderzoek wel nuttige gegevens opleveren. Een nadeel van deelname aan de studie is dat wij extra tijd van u vragen voor het invullen van de vragenlijsten en de extra metingen.

8. Wat gebeurt er als u niet wenst deel te nemen aan dit onderzoek?

U beslist zelf of u meedoet aan het onderzoek. Deelname is vrijwillig. Als u besluit niet mee te doen, hoeft u verder niets te doen. U hoeft niets te tekenen. U hoeft ook niet te zeggen waarom u niet wilt meedoen. U krijgt natuurlijk alle noodzakelijke medische behandelingen. Als u wel meedoet, kunt u zich altijd bedenken en toch nog stoppen. Dat kan ook tijdens het onderzoek. Wel moet u dit direct

For peer review only - http://bmjopen.bmj.com/site/about/guidelines.xhtml

melden aan de onderzoeker. Wij vragen u dan wel of we enkele gegevens uit uw patiëntendossier mogen gebruiken voor het onderzoek.

De gegevens die tot dat moment zijn verzameld, worden gebruikt voor het onderzoek.

Als er nieuwe informatie over het onderzoek is die belangrijk voor u is, laat de onderzoeker dit aan u weten. U wordt dan gevraagd of u blijft meedoen.

9. Wat gebeurt er als het onderzoek is afgelopen?

Uw deelname aan het onderzoek stopt als

- alle bezoeken [volgens het schema/zoals beschreven onder punt 4] voorbij zijn
- u zelf kiest om te stoppen
- de onderzoeker het beter voor u vindt om te stoppen
- de subsidiegever van het onderzoek, de overheid of de beoordelende medisch-ethische toetsingscommissie, besluit om het onderzoek te stoppen.

Het hele onderzoek is afgelopen als alle deelnemers klaar zijn.

Na het verwerken van alle gegevens informeert de onderzoeker u over de belangrijkste uitkomsten van het onderzoek.

10. Gebruik en bewaren van uw gegevens

Voor dit onderzoek is het nodig dat uw medische gegevens worden verzameld en gebruikt. Elke proefpersoon krijgt een code die op de gegevens komt te staan. Uw naam en andere persoonlijke gegevens die u direct kunnen identificeren worden daarbij weggelaten

Uw gegevens

Al uw gegevens blijven vertrouwelijk.

Sommige mensen mogen uw medische en persoonlijke gegevens inzien. Dit is om te controleren of het onderzoek goed en betrouwbaar uitgevoerd is. Algemene informatie hierover vindt u in de brochure ‘Medisch-wetenschappelijk onderzoek’.

Mensen die uw gegevens kunnen inzien zijn: het onderzoeksteam, een controleur die voor de opdrachtgever van het onderzoek werkt, de Inspectie voor de Gezondheidszorg. Zij houden uw gegevens geheim. De onderzoeker van het coördinerend centrum (Erasmus MC) ontvangt uw gegevens voor het versturen van de vragenlijsten. Deze gegevens zullen na het einde van het onderzoek vernietigd worden.

Als u de toestemmingsverklaring ondertekent, geeft u toestemming voor het verzamelen, bewaren en inzien van uw medische en persoonlijke gegevens.

De onderzoeker bewaart uw gegevens 15 jaar.

Later gebruik gegevens

For peer review only - http://bmjopen.bmj.com/site/about/guidelines.xhtml

Wij willen uw gegevens graag bewaren. Misschien kunnen we daar later extra onderzoek mee doen. Op het toestemmingsformulier kunt u aangeven of u hiermee akkoord gaat. U kunt deze toestemming altijd weer intrekken.

Inzien gegevens en intrekken toestemming

U heeft te allen tijde het recht om de over u verzamelde gegevens in te zien, te wijzigen of uw toestemming voor gebruik van uw persoonsgegevens weer in te trekken. De onderzoeksgegevens die zijn verzameld tot het moment dat u uw toestemming intrekt worden nog wel gebruikt in het onderzoek.

Meer informatie over uw rechten bij verwerking van gegevens

Voor algemene informatie over uw rechten bij verwerking van uw persoonsgegevens kunt u de website van de Autoriteit Persoonsgegevens raadplegen (https://autoriteitpersoonsgegevens.nl).

Bij vragen over uw rechten kunt u contact opnemen met de verantwoordelijke voor de verwerking van uw persoonsgegevens. Voor dit onderzoek is dat:

Erasmus Medisch Centrum: Zie bijlage C voor contactgegevens.

Bij vragen of klachten over de verwerking van uw persoonsgegevens raden we u aan eerst contact op te nemen met de onderzoekslocatie. U kunt ook contact opnemen met de Functionaris voor de Gegevensbescherming van de instelling [contactgegevens in bijlage C] of de Autoriteit Persoonsgegevens.

11. Verzekering voor proefpersonen

Voor iedereen die meedoet aan dit onderzoek is een verzekering afgesloten. De verzekering dekt schade als gevolg van het onderzoek. Dit geldt voor schade die naar boven komt gedurende het onderzoek. In bijlage A vindt u de verzekerde bedragen, de uitzonderingen en de adresgegevens van de verzekeraar.

12. Informeren huisarts

Uw huisarts wordt geïnformeerd over uw deelname aan het onderzoek. Dit is voor uw eigen veiligheid. Als u dit niet goed vindt, kunt u niet meedoen aan dit onderzoek. U kunt niet deelnemen aan het onderzoek als u geen huisarts heeft.

13. Zijn er extra kosten/is er een vergoeding wanneer u besluit aan dit onderzoek mee te doen?

Er zullen geen extra kosten in rekening worden gebracht bij deelname aan het onderzoek. Het bezoek aan de spoedeisende hulp is standaard verzekerde zorg. Het extra poliklinische bezoek na 1

For peer review only - http://bmjopen.bmj.com/site/about/guidelines.xhtml

jaar en de daarbij behorende röntgenfoto's zullen betaald worden vanuit onderzoeksgeld en dus niet aan u worden doorberekend.

14. Wilt u verder nog iets weten?

Wilt u graag een onafhankelijk advies over meedoen aan dit onderzoek? Dan kunt u terecht bij een onafhankelijke arts. Zijn gegevens vindt u in *lokale informatie onafhankelijk arts* (bijlage C).
Indien u een klacht heeft, dan kunt u deze het beste eerst bespreken met degene die er rechtstreeks bij betrokken is. Kunt u uw klacht of ongenoegen, om welke reden dan ook, niet rechtstreeks bespreken of bent u na het bespreken van uw klacht niet tevreden, dan helpt de klachtenfunctionaris u graag verder. De gegevens van de klachtenfunctionaris vindt u in bijlage C.

15. Ondertekening toestemmingsformulier

Indien u toestemming geeft over deelname aan dit onderzoek, zullen wij u vragen deze op de bijbehorende toestemmingsverklaring schriftelijk te bevestigen. Door uw schriftelijke toestemming geeft u aan dat u de informatie heeft begrepen en instemt met deelname aan het onderzoek.
Het handtekeningenblad wordt door de onderzoeker bewaard. U krijgt een kopie of een tweede exemplaar van deze toestemmingsverklaring.

Dank voor uw aandacht.

Orthopedisch chirurgen Erasmus MC en het onderzoeksteam

For peer review only

For peer review only - http://bmjopen.bmj.com/site/about/guidelines.xhtml

Polspijn zonder afwijkingen op de röntgenfoto's

Bijlagen

A. Informatie over de verzekering
B. Overzicht metingen onderzoek
C. Lokale informatie
D. Toestemmingsverklaring

For peer review only

For peer review only - http://bmjopen.bmj.com/site/about/guidelines.xhtml

Polspijn zonder afwijkingen op de röntgenfoto's

Bijlage A: Informatie over de verzekering

Voor iedereen die meedoet aan dit onderzoek is een verzekering afgesloten. De verzekering dekt schade die het gevolg is van deelname aan het onderzoek. Dit geldt voor schade die naar boven komt tijdens het onderzoek, of binnen vier jaar na het einde van het onderzoek. U moet de schade ook binnen die 4 jaar aan de verzekeraar hebben gemeld.

In geval van schade kunt u zich direct wenden tot de verzekeraar.

De verzekeraar van het onderzoek is:

CAN Insurance Company Limited,

Mevrouw Esther van Herk

+31(0)205737274

Esther.vanherk@cnahardy.com

De verzekering biedt een maximum dekking van € 650.000 per proefpersoon, met een maximumbedrag van € 5.000.000 voor het gehele onderzoek. Indien de opdrachtgever van dit onderzoek meerdere onderzoeken heeft lopen, geldt een maximumbedrag van € 7.500.000 euro per verzekeringsjaar voor álle onderzoeken. De dekking van specifieke schades en kosten is verder tot bepaalde bedragen beperkt. Deze vindt u in het *Besluit verplichte verzekering bij medisch wetenschappelijk onderzoek met mensen*. Informatie hierover kunt u vinden op de website van de Centrale Commissie Mensgebonden Onderzoek: www.ccmo.nl.

Voor deze verzekering gelden voorts een aantal uitsluitingen. De verzekering dekt niet:

- schade waarvan op grond van de aard van het onderzoek (nagenoeg) zeker was dat deze zich zou voordoen;
- schade aan de gezondheid die ook zou zijn ontstaan als u niet aan het onderzoek had deelgenomen;
- schade die het gevolg is van het (niet) volledig nakomen van aanwijzingen of instructies;
- schade aan nakomelingen, als gevolg van een nadelige inwerking van het onderzoek op u of uw nakomeling;
- bij onderzoek naar bestaande behandelmethoden: schade die het gevolg is van één van deze behandelmethoden;
- bij onderzoek naar de behandeling van specifieke gezondheidsproblemen: schade die het gevolg is van uitblijvende verbetering of van verslechtering van deze gezondheidsproblemen.

For peer review only - http://bmjopen.bmj.com/site/about/guidelines.xhtml

Polspijn zonder afwijkingen op de röntgenfoto's

	**T1** Start studie	**T 2** 2 weken na start studie	**T3** 6 weken na start studie	**T4** 3 maanden na start studie	**T5** 6 maanden na start studie	**T6** 9 maanden na start studie	**T7**12 maanden na start studie
Vragenlijsten (thuis)	+	+	+	+	+	+	+
Telefonisch contact					+	+	
Lichamelijk onderzoek (polikliniek)	*	*					+
Röntgenfoto’s (polikliniek)	*	*					+

Bijlage B: overzicht metingen onderzoek

+ = extra metingen voor onderzoek, wordt uitgevoerd / afgenomen bij deze controle
* = metingen die behoren bij gebruikelijke zorg, wordt uitgevoerd bij dit bezoek

For peer review only - http://bmjopen.bmj.com/site/about/guidelines.xhtml

Polspijn zonder afwijkingen op de röntgenfoto's

Bijlage C. Lokale informatie Erasmus Medisch Centrum

Hoofdonderzoeker:

dr. J.W. Colaris 010-7034686

orthopedisch Chirurg

(via secretariaat Orthopedie)

Onafhankelijk arts:

dr. D.E. Meuffels, 010-7034686

orthopaedisch chirurg Erasmus MC

(via secretariaat Orthopaedie)

Arts-onderzoeker:

Abigaël Cohen 06-24788338

suspect@erasmusmc.nl

Klachtencommissie:

Klachtenfunctionarissen Erasmus MC 010-7033198

Functionaris voor de Gegevensbescherming van de instelling:

Voor meer informatie over uw rechten: 010-7034986

(via secretariaat afdeling Juridische Zaken)

Autoriteit Persoonsgegevens https://autoriteitpersoonsgegevens.nl

For peer review only - http://bmjopen.bmj.com/site/about/guidelines.xhtml

Bijlage D: Toestemmingsformulier proefpersoon

Titel van het onderzoek: Polspijn zonder afwijkingen op de röntgenfoto's

- Ik heb de informatiebrief voor de proefpersoon gelezen. Ik kon aanvullende vragen stellen. Mijn vragen zijn genoeg beantwoord. Ik had genoeg tijd om te beslissen of ik meedoe.
- Ik weet dat meedoen helemaal vrijwillig is. Ik weet dat ik op ieder moment kan beslissen om toch niet mee te doen. Daarvoor hoef ik geen reden te geven.
- Ik geef toestemming om mijn huisarts te vertellen dat ik meedoe aan dit onderzoek.
- Ik weet dat sommige mensen mijn gegevens kunnen zien. Die mensen staan vermeld in deze informatiebrief.
- Ik geef toestemming om mijn gegevens te gebruiken, voor de doelen die in de informatiebrief staan.
- Ik geef toestemming om mijn onderzoeksgegevens 15 jaar na afloop van dit onderzoek te bewaren.
- Ik geef toestemming om in de toekomst opnieuw benaderd te worden voor vervolg onderzoek. Indien ik dit niet wens, zal ik dat kenbaar maken.
- Ik wil meedoen aan dit onderzoek.

Naam proefpersoon: Handtekening:

Datum : __ / __ / __

Ik verklaar hierbij dat ik deze proefpersoon volledig heb geïnformeerd over het genoemde onderzoek.

Als er tijdens het onderzoek informatie bekend wordt die de toestemming van de proefpersoon zou kunnen beïnvloeden, dan breng ik hem/haar daarvan tijdig op de hoogte.

Naam onderzoeker (of diens vertegenwoordiger):

Handtekening: Datum: __ / __ / __

* Doorhalen wat niet van toepassing is.

For peer review only - http://bmjopen.bmj.com/site/about/guidelines.xhtml

SPIRIT

STANDARD PROTOCOL ITEMS: RECOMMENDATIONS FOR INTERVENTIONAL TRIALS

SPIRIT 2013 Checklist: Recommended items to address in a clinical trial protocol and related documents*

Section/item	ItemNo	Description	Page
**Administrative information**			
Title	1	Descriptive title identifying the study design, population, interventions, and, if applicable, trial acronym	1
Trial registration	2a	Trial identifier and registry name. If not yet registered, name of intended registry	3
	2b	All items from the World Health Organization Trial Registration Data Set	n/a
Protocol version	3	Date and version identifier	footer
Funding	4	Sources and types of financial, material, and other support	3
Roles and responsibilities	5a	Names, affiliations, and roles of protocol contributors	1-3
	5b	Name and contact information for the trial sponsor	2
	5c	Role of study sponsor and funders, if any, in study design; collection, management, analysis, and interpretation of data; writing of the report; and the decision to submit the report for publication, including whether they will have ultimate authority over any of these activities	3
	5d	Composition, roles, and responsibilities of the coordinating centre, steering committee, endpoint adjudication committee, data management team, and other individuals or groups overseeing the trial, if applicable (see Item 21a for data monitoring committee)	n/a

For peer review only - http://bmjopen.bmj.com/site/about/guidelines.xhtml

			Page
**Introduction**			
Background and rationale	6a	Description of research question and justification for undertaking the trial, including summary of relevant studies (published and unpublished) examining benefits and harms for each intervention	5-6
	6b	Explanation for choice of comparators	5-6
Objectives	7	Specific objectives or hypotheses	6
Trial design	8	Description of trial design including type of trial (eg, parallel group, crossover, factorial, single group), allocation ratio, and framework (eg, superiority, equivalence, noninferiority, exploratory)	6
**Methods: Participants, interventions, and outcomes**			
Study setting	9	Description of study settings (eg, community clinic, academic hospital) and list of countries where data will be collected. Reference to where list of study sites can be obtained	7
Eligibility criteria	10	Inclusion and exclusion criteria for participants. If applicable, eligibility criteria for study centres and individuals who will perform the interventions (eg, surgeons, psychotherapists)	8
Interventions	11a	Interventions for each group with sufficient detail to allow replication, including how and when they will be administered	9
	11b	Criteria for discontinuing or modifying allocated interventions for a given trial participant (eg, drug dose change in response to harms, participant request, or improving/worsening disease)	9
	11c	Strategies to improve adherence to intervention protocols, and any procedures for monitoring adherence (eg, drug tablet return, laboratory tests)	9
	11d	Relevant concomitant care and interventions that are permitted or prohibited during the trial	9

For peer review only - http://bmjopen.bmj.com/site/about/guidelines.xhtml

			Page
Outcomes	12	Primary, secondary, and other outcomes, including the specific measurement variable (eg, systolic blood pressure), analysis metric (eg, change from baseline, final value, time to event), method of aggregation (eg, median, proportion), and time point for each outcome. Explanation of the clinical relevance of chosen efficacy and harm outcomes is strongly recommended	9-13
Participant timeline	13	Time schedule of enrolment, interventions (including any run-ins and washouts), assessments, and visits for participants. A schematic diagram is highly recommended (see Figure)	Figure 1 Table 1 Table 2
Sample size	14	Estimated number of participants needed to achieve study objectives and how it was determined, including clinical and statistical assumptions supporting any sample size calculations	14-15
Recruitment	15	Strategies for achieving adequate participant enrolment to reach target sample size	15
**Methods: Assignment of interventions (for controlled trials)**			
Allocation:			
Sequence generation	16a	Method of generating the allocation sequence (eg, computer-generated random numbers), and list of any factors for stratification. To reduce predictability of a random sequence, details of any planned restriction (eg, blocking) should be provided in a separate document that is unavailable to those who enrol participants or assign interventions	9
Allocation concealment mechanism	16b	Mechanism of implementing the allocation sequence (eg, central telephone; sequentially numbered, opaque, sealed envelopes), describing any steps to conceal the sequence until interventions are assigned	9
Implementation	16c	Who will generate the allocation sequence, who will enrol participants, and who will assign participants to interventions	7
Blinding (masking)	17a	Who will be blinded after assignment to interventions (eg, trial participants, care providers, outcome assessors, data analysts), and how	9

For peer review only - http://bmjopen.bmj.com/site/about/guidelines.xhtml

	17b	If blinded, circumstances under which unblinding is permissible, and procedure for revealing a participant's allocated intervention during the trial	n/a
**Methods: Data collection, management, and analysis**			
Data collection methods	18a	Plans for assessment and collection of outcome, baseline, and other trial data, including any related processes to promote data quality (eg, duplicate measurements, training of assessors) and a description of study instruments (eg, questionnaires, laboratory tests) along with their reliability and validity, if known. Reference to where data collection forms can be found, if not in the protocol	9-14
	18b	Plans to promote participant retention and complete follow-up, including list of any outcome data to be collected for participants who discontinue or deviate from intervention protocols	15-16
Data management	19	Plans for data entry, coding, security, and storage, including any related processes to promote data quality (eg, double data entry; range checks for data values). Reference to where details of data management procedures can be found, if not in the protocol	19
Statistical methods	20a	Statistical methods for analysing primary and secondary outcomes. Reference to where other details of the statistical analysis plan can be found, if not in the protocol	15-17
	20b	Methods for any additional analyses (eg, subgroup and adjusted analyses)	15-17
	20c	Definition of analysis population relating to protocol non-adherence (eg, as randomised analysis), and any statistical methods to handle missing data (eg, multiple imputation)	16
**Methods: Monitoring**			
Data monitoring	21a	Composition of data monitoring committee (DMC); summary of its role and reporting structure; statement of whether it is independent from the sponsor and competing interests; and reference to where further details about its charter can be found, if not in the protocol. Alternatively, an explanation of why a DMC is not needed	18

For peer review only - http://bmjopen.bmj.com/site/about/guidelines.xhtml

			Page
	21b	Description of any interim analyses and stopping guidelines, including who will have access to these interim results and make the final decision to terminate the trial	n/a
Harms	22	Plans for collecting, assessing, reporting, and managing solicited and spontaneously reported adverse events and other unintended effects of trial interventions or trial conduct	18
Auditing	23	Frequency and procedures for auditing trial conduct, if any, and whether the process will be independent from investigators and the sponsor	18
**Ethics and dissemination**			
Research ethics approval	24	Plans for seeking research ethics committee/institutional review board (REC/IRB) approval	19
Protocol amendments	25	Plans for communicating important protocol modifications (eg, changes to eligibility criteria, outcomes, analyses) to relevant parties (eg, investigators, REC/IRBs, trial participants, trial registries, journals, regulators)	19
Consent or assent	26a	Who will obtain informed consent or assent from potential trial participants or authorised surrogates, and how (see Item 32)	7
	26b	Additional consent provisions for collection and use of participant data and biological specimens in ancillary studies, if applicable	n/a
Confidentiality	27	How personal information about potential and enrolled participants will be collected, shared, and maintained in order to protect confidentiality before, during, and after the trial	19
Declaration of interests	28	Financial and other competing interests for principal investigators for the overall trial and each study site	21
Access to data	29	Statement of who will have access to the final trial dataset, and disclosure of contractual agreements that limit such access for investigators	19
Ancillary and post-trial care	30	Provisions, if any, for ancillary and post-trial care, and for compensation to those who suffer harm from trial participation	18

For peer review only - http://bmjopen.bmj.com/site/about/guidelines.xhtml

			Page
Dissemination policy	31a	Plans for investigators and sponsor to communicate trial results to participants, healthcare professionals, the public, and other relevant groups (eg, via publication, reporting in results databases, or other data sharing arrangements), including any publication restrictions	19
	31b	Authorship eligibility guidelines and any intended use of professional writers	21
	31c	Plans, if any, for granting public access to the full protocol, participant-level dataset, and statistical code	n/a
**Appendices**			
Informed consent materials	32	Model consent form and other related documentation given to participants and authorised surrogates	
Biological specimens	33	Plans for collection, laboratory evaluation, and storage of biological specimens for genetic or molecular analysis in the current trial and for future use in ancillary studies, if applicable	n/a

*It is strongly recommended that this checklist be read in conjunction with the SPIRIT 2013 Explanation & Elaboration for important clarification on the items. Amendments to the protocol should be tracked and dated. The SPIRIT checklist is copyrighted by the SPIRIT Group under the Creative Commons "Attribution-NonCommercial-NoDerivs 3.0 Unported" license.

BMJ Open

clinically SUspected ScaPhoid fracture: treatment with supportive bandage or CasT? "Study protocol of a multicenter randomized controlled trial" (SUSPECT study)

Journal:	*BMJ Open*
Manuscript ID	bmjopen-2020-036998.R2
Article Type:	Protocol
Date Submitted by the Author:	12-Aug-2020
Complete List of Authors:	Cohen, Abigael; Erasmus MC, Department of Orthopaedics<br>Reijman, M; Erasmus MC, Department of Orthopaedics<br>Kraan, Gerald; Reinier de Graaf Hospital, Department of Orthopaedics<br>Mathijssen, Nina; Reinier de Graaf Hospital, Department of Orthopaedics<br>Koopmanschap, Marc; Erasmus University Rotterdam, Erasmus School of Health Policy and Management<br>Verhaar, Jan; Erasmus MC, Department of Orthopaedics<br>Mol, Sander; Franciscus Gasthuis en Vlietland, Department of Emergency Medicine<br>Colaris, Joost; Erasmus MC, Department of Orthopaedics
**Primary Subject Heading**:	Surgery
Secondary Subject Heading:	Health economics, Emergency medicine
Keywords:	Adult orthopaedics < ORTHOPAEDIC & TRAUMA SURGERY, Trauma management < ORTHOPAEDIC & TRAUMA SURGERY, Hand & wrist < ORTHOPAEDIC & TRAUMA SURGERY, ACCIDENT & EMERGENCY MEDICINE

SCHOLARONE™
Manuscripts

I, the Submitting Author has the right to grant and does grant on behalf of all authors of the Work (as defined in the below author licence), an exclusive licence and/or a non-exclusive licence for contributions from authors who are: i) UK Crown employees; ii) where BMJ has agreed a CC-BY licence shall apply, and/or iii) in accordance with the terms applicable for US Federal Government officers or employees acting as part of their official duties; on a worldwide, perpetual, irrevocable, royalty-free basis to BMJ Publishing Group Ltd ("BMJ") its licensees and where the relevant Journal is co-owned by BMJ to the co-owners of the Journal, to publish the Work in this journal and any other BMJ products and to exploit all rights, as set out in our licence.

The Submitting Author accepts and understands that any supply made under these terms is made by BMJ to the Submitting Author unless you are acting as an employee on behalf of your employer or a postgraduate student of an affiliated institution which is paying any applicable article publishing charge ("APC") for Open Access articles. Where the Submitting Author wishes to make the Work available on an Open Access basis (and intends to pay the relevant APC), the terms of reuse of such Open Access shall be governed by a Creative Commons licence – details of these licences and which Creative Commons licence will apply to this Work are set out in our licence referred to above.

Other than as permitted in any relevant BMJ Author's Self Archiving Policies, I confirm this Work has not been accepted for publication elsewhere, is not being considered for publication elsewhere and does not duplicate material already published. I confirm all authors consent to publication of this Work and authorise the granting of this licence.

For peer review only

clinically SUspected ScaPhoid fracturE: treatment with supportive bandage or CasT? "Study protocol of a multicenter randomized controlled trial" (SUSPECT study)

Abigael Cohen[1], Max Reijman[1] , Gerald A Kraan[2], Nina M C Mathijssen[2], Marc A Koopmanschap[3], Jan A N Verhaar[1], Sander Mol[5], Joost W Colaris[1], on behalf of the SUSPECT study group

SUSPECT study group:

Peer van der Zwaal[6], Merel van Loon[7], Steven J Rhemrev[8], Daphne A van Rijssel[9], Mark R de Vries[10], Flip van Beek[11], Vanessa Brown[5], Tjebbe Hagenaars[12], Els H Jansen[13], Alexander P A Greeven[14], Lenneke C M Scholtens[15], Ruud L M Deijkers[16], Niels W L Schep[17], Akkie N Ringburg[18], Milko M M Bruijninckx[19], Kirsten F van Meerten[20], Willem-Maarten P F Bosman[21]

1 Department of Orthopaedic Surgery, Erasmus MC University Medical Center, Rotterdam, The Netherlands

2 Department of Orthopaedic Surgery, Reinier de Graaf Hospital, Delft, The Netherlands

3 Erasmus School of Health Policy and Management, Erasmus University Rotterdam, Rotterdam, The Netherlands

5 Department of Emergency Medicine, Franciscus Hospital, Rotterdam, The Netherlands

6 Department of Orthopaedic Surgery, Haaglanden Medical Centre, The Hague, The Netherlands

7 Department of Emergency Medicine, Haaglanden Medical Centre, The Hague, The Netherlands

8 Department of Surgery, Haaglanden Medical Centre, The Hague, The Netherlands,

9 Department of Emergency Medicine, Reinier de Graaf Hospital, Delft, The Netherlands

10 Department of Surgery, Reinier de Graaf Hospital, Delft, The Netherlands

11 Department of Surgery, Franciscus Hospital, Rotterdam, The Netherlands

For peer review only - http://bmjopen.bmj.com/site/about/guidelines.xhtml

12 Trauma Research Unit, Department of Surgery, Erasmus MC University Medical Center, Rotterdam, The Netherlands

13 Department of Emergency Medicine, Erasmus MC University Medical Center, Rotterdam, The Netherlands

14 Department of Surgery, Haga Teaching Hospital, The Hague, The Netherlands

15 Department of Emergency Medicine, Haga Teaching Hospital, The Hague, The Netherlands

16 Department of Orthopaedic Surgery, Haga Teaching Hospital, The Hague, The Netherlands

17 Department of Surgery, Maasstad Hospital, Rotterdam, The Netherlands

18 Department of Surgery, Ikazia Hospital, Rotterdam, The Netherlands

19 Department of Surgery, Ijsselland Hospital, Capelle aan den Ijssel, The Netherlands

20 Department of Emergency Medicine, Albert Schweitzer Hospital, Dordrecht, The Netherlands

21 Department of Surgery, Albert Schweitzer Hospital, Dordrecht, The Netherlands

Corresponding Author: Abigael Cohen, Telephone +31107043982, Email: a.cohen.1@erasmusmc.nl Department of Orthopedic Surgery, Erasmus MC University Medical Center, PO box 2040, 3000 CA Rotterdam, the Netherlands

Trial sponsor: Prof. dr. J.A.N. Verhaar, Email: j.verhaar@erasmusmc.nl Department of Orthopedic Surgery, Erasmus University MC Medical Center, PO box 2040, 3000 CA Rotterdam, the Netherlands

Authors' Contributions

MR and JC conceived the study idea and secured funding. AC, MR, GK, NM, MK, JV, SM and JC were involved with the initial study design. MR and MK were involved with the statistical analysis for the study. AC, GK, SM, JC, SUSPECT study group are involved with the implementation of the protocol

For peer review only

For peer review only - http://bmjopen.bmj.com/site/about/guidelines.xhtml

and the data collection. AC is conducting the study. All authors provided critical comments to the manuscript and approved the final version before submitting.

Funding statement

For this study, we received a grant from ZonMw, a Dutch organization for health research and care innovation and co-funding from CZ, a Dutch health insurance company. Both organizations were not involved in the study design.

Competing interests

None of the authors declared competing interests.

Trial registration

This study was prospectively registered at the Netherlands National Trial Register on February 28th, 2018, (www.trialregister.nl, NL6976)

Word count: 4022

Keywords: Scaphoid Bone, Fractures, Bone, Recovery of Function, Cost-Benefit Analysis, Wrist injuries.

ABSTRACT

Introduction: Some scaphoid fractures become visible on radiographs weeks after a trauma which makes normal radiographs directly after trauma unreliable. Untreated scaphoid fractures can lead to scaphoid non-union progressing to osteoarthritis. Therefore, the general treatment for patients with a clinically suspected scaphoid fracture and normal initial radiographs, is immobilization with below-elbow cast for 2 weeks. However, most of these patients are treated unnecessary because eventually

For peer review only - http://bmjopen.bmj.com/site/about/guidelines.xhtml

less than 10% of them are diagnosed with an occult scaphoid fracture. To reduce overtreatment and costs as a result of unnecessary cast treatment in patients with a clinically suspected scaphoid fracture and normal initial radiographs, we designed a study to compare treatment with below-elbow cast or supportive bandage. We hypothesize that the functional outcome after 3 months is similar in both groups, but with lower costs in the supportive bandage group.

Methods and analysis: The SUSPECT study is an open-labeled multicenter randomized controlled trial with non-inferiority design. A total of 180 adult patients with a clinically suspected scaphoid fracture and normal initial radiographs are randomized between two groups: 3 days supportive bandage or 14 days below-elbow cast. We aim to evaluate the functional outcome and cost-effectiveness of both treatments. The primary outcome is the difference in functional outcome after 3 months between both groups, assessed with the Quick Disability of the Arm, Shoulder and Hand score. Secondary outcomes include post-traumatic injuries, functional outcome, recovery of function, pain, patient satisfaction, quality of life and cost-effectiveness measured by medical consumption, absence from work or decreased productivity.

Ethics and dissemination: The medical ethics committee of the Erasmus MC Medical Centre, Rotterdam approved the study protocol (MEC-2017-504). We plan to present the results after completion of the study at (inter)national conferences and publish in general peer-reviewed journals.

Trial registration: www.trialregister.nl, NL6976, February 28th, 2018 registered.

STRENGTHS AND LIMITATIONS OF THIS STUDY

- This is the first multicenter randomized controlled trial to evaluate if treatment with supportive bandage results in not inferior functional outcome to below-elbow cast in patients with a clinically suspected scaphoid fracture.
- All patients are re-evaluated clinically and radiographically after one year.

For peer review only - http://bmjopen.bmj.com/site/about/guidelines.xhtml

- The cost-effectiveness of the treatment up to one year is studied.
- The main limitation of the study is that the study is not blinded for participants, physicians, and researchers.

INTRODUCTION

Up to 32% of all scaphoid fractures results in non-union, leading to progressive osteoarthritis,(1). While most fractures are visible on radiographs directly after trauma, not all scaphoid fractures can be diagnosed on the initial radiographs. To avoid untreated occult scaphoid fractures, all patients with a clinically suspected scaphoid fracture and normal initial radiographs are treated with a below-elbow cast until re-examination or further diagnostics,(2, 3). Finally, less than 10% of these patients appear to have a scaphoid fracture resulting in a large number of overtreatment, unnecessary absenteeism from work and increased health care costs,(4, 5).

Most occult scaphoid fractures can be diagnosed on repeated conventional radiography after 10-14 days (6). These occult fractures are in general non-displaced fractures of the waist or distal pole of the scaphoid, in which an intact periosteal envelop provides stability,(7, 8). Known risk factors for scaphoid non-union are proximal fracture location, fracture displacement and start of treatment more than 4 weeks after trauma (8-10). When adequate treatment for a scaphoid fracture is started within 4 weeks after the trauma, the non-union rate does not increase,(8). Therefore, supportive bandage and re-examination within 2 weeks can be a good alternative treatment for patients with a clinically suspected scaphoid fracture with normal radiographs. In 1988, one randomized study of 106 patients with a clinically suspected scaphoid fracture showed that treatment with supportive bandage compared with below-elbow cast in these patients decreased the immobilization time and absence of work without negative consequences on fracture healing. However, functional outcome and cost-effectiveness were not evaluated, and the follow-up period was not clearly stated,(8).

We designed a pragmatic, randomized controlled trial with 1 year follow up to evaluate the functional outcome and cost-effectiveness of treatment with a supportive bandage compared with

For peer review only - http://bmjopen.bmj.com/site/about/guidelines.xhtml

For peer review only

below-elbow-cast in patients with a clinically suspected scaphoid fracture and normal initial radiographs. We hypothesize that treatment with supportive bandage results in an not inferior functional outcome compared to below-elbow cast, with lower costs in the supportive bandage group.

Objectives

We designed a pragmatic, multicenter randomized controlled trial with a non-inferiority design with two groups. The primary objective of this study is to determine if treatment with supportive bandage compared with below-elbow cast in patients with clinically suspected scaphoid fracture and normal initial radiographs, results in not inferior functional outcome after 3 months, measured with the Quick Disabilities of the Arm, Shoulder and Hand (QDASH).

As secondary objectives, we assess whether treatment of patients with a clinically suspected scaphoid fracture with a bandage is cost-effective compared to cast. Cost-effectiveness is evaluated by medical consumption, absence from work or decreased productivity, and quality of life, measured in Quality-adjusted life years (QALYs).

Furthermore, we assess if supportive bandage compared to cast results in not inferior pain, patient satisfaction of the received treatment, functional outcome measured with the QDASH, recovery of function measured with physical examination and the Patient Rated Wrist/Hand Evaluation (PRWHE), and quality of life.

METHODS AND ANALYSIS

This manuscript is written according to the Consolidated Standards for Reporting Trials (CONSORT statement) and Standard Protocol Items: Recommendations for Interventional Trials (SPIRIT guidelines),(11, 12).

Patient and Public Involvement statement

For peer review only

For peer review only - http://bmjopen.bmj.com/site/about/guidelines.xhtml

In the absence of a patient association, we formed a panel of 3 patients with a (suspected) scaphoid fracture to think along with, and comment on our study. This panel of patients analyzed the study (aim, burden for patient, willingness to participate), patient information sheet and how the future results should be reported to the patients. To improve our study, we evaluate the study procedures and the patient burden with participating patients during the recruitment period. After termination of the study, we present our study results to all participating patients.

Study design

The SUSPECT study is performed in 9 hospitals in the Netherlands. All departments involved in the treatment of patients with a clinically suspected scaphoid fracture in each participating hospital participate in the study.

All patients who present with a clinically suspected scaphoid fractureafter trauma at the emergency department (ED), who match the in- and exclusion criteria, are informed about the study by their treating physician. These patients receive the participant information sheet and are invited to ask questions about the study. As direct start of treatment is necessary, patients have to decide on study participation after the information is provided at the ED. Written informed consent is obtained from the patient prior to inclusion (Supplementary appendix 1). All included patients are randomized at the ED and allocated to treatment with supportive bandage or below-elbow cast.

Patients revisit the outpatient clinic after 14 days and 1 year. After 14 days, the researcher re-examines the patient first. Secondly, radiographs with scaphoid specific views are obtained (without cast or bandage around the wrist) according to the hospital protocol. Lastly, the physician re-examines the patient and determines the diagnosis, treatment and follow-up according to their hospital protocol without interference of the researcher . When a scaphoid fracture is diagnosed by the physician, patients are treated for a scaphoid fracture according hospital protocol with either

For peer review only - http://bmjopen.bmj.com/site/about/guidelines.xhtml

cast or surgery. After 1 year, the researcher re-examines the patients and wrist radiographs are made.

During the follow up period, patients receive questionnaires by email after inclusion, 2 weeks, 6 weeks, 3 months, 6 months, 9 months and 1 year of follow up.

Due to the Dutch privacy law, it is not allowed to screen eligible patients at the ED to detect how many eligible patients are not participating in our study.

For peer review only

Study population

All adult patients who visit the ED of the participating hospitals, within 48 hours after trauma with a clinically suspected scaphoid fracture without a fracture on the radiograph, are invited to participate in the study.

Inclusion criteria

- Age of 18 years or older
- Trauma maximum 48 hours before
- Anatomical snuff box (ASB) tenderness or scaphoid tubercle (ST) tenderness
- No scaphoid fracture reported by the radiologist or treating physician on the initial radiographs with scaphoid specific views (minimally 3 views)

Exclusion criteria

- Concomitant injury of the ipsilateral extremity that needs treatment with movement restriction by cast or supportive bandage.
- Inability to complete study forms due to insufficient command of the Dutch language.

For peer review only - http://bmjopen.bmj.com/site/about/guidelines.xhtml

- A scaphoid fracture diagnosed on the initial radiographs by a supervising radiologist in retrospect.

Randomization

After written informed consent is obtained, patients are central randomized through computer-based variable block randomization (2, 4, 6 blocks) by Castor Electronic Data Capture (EDC) to enable allocation concealment,(13). The allocation ratio is 1:1 and randomization is stratified per hospital due to potential differences in applied cast and the number of scaphoid specific views per hospital (Figure 1).

Because of practical reasons, there is no blinding of the treatment group for the physician, patient and researcher, but assessment of the radiographs is blinded. We will perform statistical analysis blinded for allocated treatment. .

Interventions

Intervention group (supportive bandage)

Patients in the intervention group receive a below-elbow supportive bandage for 3 days at the ED. After 3 days the patients are allowed to remove the bandage and move the wrist driven by the amount of pain. When patients experience too much pain despite adequate analgesia, an additional appointment is made at the outpatient clinic to apply a below-elbow cast for analgesic purposes. A similar below-elbow cast, to the control group, is applied conform hospital protocol. The patients that cross-over to cast remain in the study.

Control group (below-elbow cast)

For peer review only - http://bmjopen.bmj.com/site/about/guidelines.xhtml

220 The usual care in the Netherlands for patients with clinically suspected scaphoid fracture at the ED is below-elbow cast and re-examination after 14 days (Figure 2),(2). The applied below-elbow cast type is according to hospital protocol and can be circular or splint, with or without the thumb included.

Outcome

We collect the following baseline variables; demographic data (e.g. age, gender, height, weight, hand dominance), details about the trauma (e.g. fall onto an outstretched hand (FOOSH), high-energy trauma, low-energy trauma, sports injury) and comorbidities such as smoking status, previous arm injuries and general medical history.

Primary outcome

The primary outcome is the difference in functional outcome between the intervention and control group measured with the QDASH after 3 months of follow-up. We selected the QDASH as the primary outcome, as it takes the function of both hands into account. With this information, we can reflect if the wrist problem results in an impaired functional outcome in daily life.

Secondary outcomes

- Functional outcome measured with the QDASH after inclusion and at 2 weeks, 6 weeks and 1 year of follow up (Table 1).
- Recovery of function evaluated with PRWHE after inclusion and at 2 weeks, 6 weeks, 3 months and 1 year of follow up.
- The amount of pain scored with the Visual Analogue Scale (VAS) score at rest and during movement after inclusion and at 2 weeks, 6 weeks, 3 months and 1 year of follow up.
- Patient satisfaction of the treatment is measured after 2 weeks and 3 months of follow up.

For peer review only - http://bmjopen.bmj.com/site/about/guidelines.xhtml
For peer review only

- Quality of life assessed by the 5-level EuroQol-5D (EQ-5D-5L) after inclusion and at 2 weeks, 6 weeks, 3 months and 1 year of follow up. When the patient reports medical consumption or decreased productivity due the wrist problems after 6 or 9 months of follow up, the EQ-5D-5L is sent to the patients as well.
- To assess the costs and cost-effectiveness of both interventions, we collect information on diagnostic (additional imaging) and therapeutic hospital procedures using the electronic patient status and questionnaires. We use iMTA Medical Consumption Questionnaire (iMCQ) and iMTA Productivity Cost Questionnaire (iPCQ) after 6 weeks, 3 months, 6 months, 9 months and 1 year of follow up (Table 1).
- Physical examination is performed at inclusion, after 2 weeks and 1 year of follow up. At inclusion, the physical examination is performed by the treating physician. They will register if the patient has tenderness ASB, tenderness ST, compression pain thumb, radial sided wrist pain with resisted supination and radial sided wrist pain with ulnar deviation. A previous study showed that these clinical tests have the highest sensitivity for a scaphoid fracture,(14). After 2 weeks and 1 year, the function of the wrist is assessed by the researcher. The physical examination includes the abovementioned clinical tests and swelling or hematoma on the radial side of the wrist. Range of motion of the wrist (palmar flexion, dorsal flexion, ulnar deviation, radial deviation, supination, and pronation) and finger-to-palm distance are measured with a goniometer. The finger-to-palm distance is the distance from the tip of the finger to the distal palmar crease, when the fingers are in maximal active flexion,(15). We assess opposition of the thumb with the Kapandji score,(16). The Kapandji score ranges from 0 – 10, whereas 0 indicates no opposition and 10 maximal opposition. Grip strength is measured with a hand dynamometer (Jamar hand dynamometer), and is defined as the maximum grip strength after 3 attempts. Range of motion and grip strength of both wrists are examined. One year after inclusion, we additional test the scapholunate (SL) ligament by identifying tenderness over the dorsal SL interval, performing the finger

For peer review only - http://bmjopen.bmj.com/site/about/guidelines.xhtml

Table 1 Description of patient-rated outcome measures and examinations per follow up moment.

	Baseline	2 weeks	6 weeks	3 months	6 months	9 months	1 year
**Radiograph**	Scaphoid	Scaphoid					Wrist
**Physical examination**	x	x					x
**PROM**	QDASH PRWHE	QDASH PRWHE	QDASH PRWHE	QDASH PRWHE	iMCQ iPCQ	iMCQ iPCQ	QDASH PRWHE

extension test, and the Watson test (Table 2). The finger extension test is positive when resisted extension of the fingers with the wrist in flexion is painful,(17). To perform the Watson test, the patients' wrist is in dorsal flexion and ulnar deviation, with the researchers thumb on ST and fingers wrapped around the distal radius. Then, the researcher moves the patients' wrist radially and in palmar flexion. The Watson test is positive if a painful clunk is noticed,(17, 18).

- Radiographic re-examination is performed after 2 weeks and 1 year of follow up. After 2 weeks, the radiographs include scaphoid specific views (minimally 3 views) to identify a scaphoid fracture. After 1 year, a posterior-anterior and lateral wrist radiographs are made to determine the presence of any post-traumatic injuries (e.g. scaphoid non-union, SL dissociation). The radiographs are evaluated by two independent assessors.
- Detailed information about the received treatment is reported. We address the number of patients that cross over from their allocated treatment , days of cast or supportive bandage before the outpatient department appointment and applied treatment after the outpatient department appointment (cast or surgery).
- (Serious) adverse events are noted, such as scaphoid fracture and scaphoid nonunion. (Detailed information can be found at safety considerations).
- Other diagnosis are reported such as other carpal fractures or ligament injuries.

For peer review only

For peer review only - http://bmjopen.bmj.com/site/about/guidelines.xhtml

	VAS<br>EQ-5D-5L	VAS<br>Patient satisfaction<br>EQ-5D-5L	VAS<br>EQ-5D-5L<br>iMCQ<br>iPCQ	VAS<br>Patient satisfaction<br>EQ-5D-5L<br>iMCQ<br>iPCQ	(EQ-5D-5L)	(EQ-5D-5L)	VAS<br>EQ-5D-5L<br>iMCQ<br>iPCQ

PROM, Patient-Rated Outcome Measure; QDASH, *Quick Disability of the Arm, Shoulder and Hand; PRWHE, Patient Rated Wrist Hand Evaluation; VAS, Visual Analog Score; EQ-5D-5L, 5-level EuroQol-5D; iMCQ, iMTA Medical Consumption Questionnaire; iPCQ, iMTA Productivity Cost Questionnaire.*

Table 2 Physical examination per follow up moment

	Baseline	2 weeks	1 year
Swelling radial side wrist		x	x
Tenderness ST	x	x	x
Compression pain thumb	x	x	x
Painful resisted supination	x	x	x
Painful UD	x	x	x
SL ligament			x
Kapandji score		x	x
Range of motion		x	x
Finger-to-palm distance		x	x
Grip strength		x	x

UD, Ulnar deviation; ASB, *Anatomical snuff box; ST, Scaphoid Tubercle; SL, Scapholunate ligament examinations consist of tenderness SL interval, Finger extension test and Watson test. Range of motion include palmar flexion, dorsal flexion, ulnar deviation, radial deviation, supination and pronation.*

Instruments

For peer review only - http://bmjopen.bmj.com/site/about/guidelines.xhtml

Functional outcome is measured with the mandatory module of the QDASH. The QDASH is the short version of the DASH and consists of 11 questions about symptoms and physical function of both arms during the last week. Each question can be scored from 1 - 5. A high score indicates severe disability of the upper extremity. The Dutch QDASH has shown good validity, reliability and responsiveness for comparable patients,(19, 20).

The PRWHE questionnaire evaluates the painful wrist during the last week. The questionnaire consists of three subscales about pain, function, and cosmetics. The cosmetic field is not included in the score. The pain and function subscales consist each of 15 questions that can be scored from 0-10. A high PRWHE score indicates severe pain and restricted function of the painful wrist. The Dutch PRWHE has shown good validity, reliability and responsiveness for comparable patients,(20) .

Pain scores at rest and during movement are examined with the eleven point Visual Analogue Scale (VAS) for pain. A high VAS score indicates severe pain. The VAS for pain is widely used in research with comparable patients,(21).

Patient satisfaction is assessed by the questions 'how satisfied are you with the treatment', 'did you prefer the other treatment?', and 'if yes, why?'. The first question can be scored from 0-10. A high score indicates satisfaction.

Quality of life is measured with the EQ-5D-5L. This questionnaire consists of five questions and a visual analogue scale. The questions are about mobility, self-care, usual activities, pain or discomfort, and anxiety or depression. Each question can be scored as: no problem, slight problem, moderate problem, severe problem, and extreme problem. Health is reported on a visual analogue scale from 0-100. A high score indicates better health. The EQ-5D-5L is recommended for the assessment of quality of life in trauma patients, especially for economic assessments,(22).

To assess the costs and cost-effectiveness of the intervention with the usual care we include the Medical Consumption Questionnaire (iMCQ) and Productivity Cost Questionnaire (iPCQ). The iMCQ measures the total medical consumption by measuring the use of diagnostics, consultations, emergency department visits, physical therapy, medication, and aids. The iPCQ measures

For peer review only - http://bmjopen.bmj.com/site/about/guidelines.xhtml

productivity costs. This questionnaire evaluates costs of absence of work and decreased productivity at paid or unpaid work. The iMCQ and iPCQ s are short generic measurement instruments,(23).

Sample size

Our hypothesis is that patients with a clinically suspected scaphoid fracture with normal initial radiographs treated with a bandage have not inferior functional outcome after 3 months compared with cast, but with lower costs. The power calculation is based on the proof of non-inferiority. We took 50% of the margin of clinical minimal important difference of QDASH,(24). This resulted in a margin of non-inferiority of 7.50 points on the QDASH. The standard deviation of QDASH after 3 months has been reported to be 14,(25). We used a power of 90% and a one-sided alpha of 0.025. To detect non-inferiority of bandage compared to cast 148 patients are needed (74 patients per group). Accounting for a 15% lost to follow-up, a total of 180 patients are required. When the lost to follow-up or change of allocated treatment is higher than 15%, we will include more patients to have 74 patients per group to answer our primary research question. When the patient recruitment rate at the ED is behind our expectations, we invite more hospitals to participate.

Data collection

We will collect data by electronic data capture with Castor EDC,(13). Paper based case report forms are used at the ED and the outpatient department. The researcher inserts this data in Castor EDC. Questionnaires are sent directly to the patients through email via Castor EDC. In case an email address is not available, a paper case report form is sent to the patient. When the patient does not respond, we will contact the patient by telephone.

We screen the electronic patient status to collect information about (serious) adverse events, hospital visits, additional imaging and treatment for the wrist injury during the follow-up period of the study.

For peer review only - http://bmjopen.bmj.com/site/about/guidelines.xhtml

Data analysis

We will perform all analyses blinded for treatment allocation. The distribution analysis of baseline variables is tested by the Shapiro-Wilk test. For normally distributed variables, parametric tests are used. For those variables that are not normally distributed, non-parametric tests are used. We report the proportion of diagnosis and adverse events per allocated treatment group.

All outcomes are analyzed 'by intention to treat analysis' with a linear mixed model with specified fixed and random effects. The covariance structure is unstructured. Restricted maximum likelihood will be used to estimate parameters. The assumptions of our model (linearity, homoscedasticity and normal distribution of the error terms) are tested. Should any of these assumptions seriously fail, variable transformations are used.

For our primary outcome, QDASH at 3 months, and secondary outcomes PRWHE, VAS score, EQ5D-5L at measurement time, and an interaction term of measurement time by treatment. Hospital is a random intercept and to analyze repeated measurements, we add a random intercept for patients (nested with hospital). Baseline, and 2 weeks, 6 weeks, and 3 months follow up are included in our model as reapeated measures. We perform post-hoc analyses to compare estimated means at 3 months. All outcomes are assessed for non-inferiority, expect cost-effectiveness which is assessed for superiority. A margin of non-inferiority of 7.50 is used for our primary outcome in the analyses. Since our groups are small, it can be that the baseline characteristics are not in balance between the 2 groups. Therefore, we add the potential confounders as age, gender and presence of comorbidities that influence the function of the arms, as fixed effects.We perform additional as-treated analysis to analyze outcomes between patients that received only supportive bandage until the outpatient department appointment after 2 weeks and patients that received cast in the first 2 weeks (received cast at the ED or crossover from bandage). Patients are included in our mixed models when they responded to the outcome questionnaire irrespective of the number of timepoints. When

For peer review only - http://bmjopen.bmj.com/site/about/guidelines.xhtml

randomized patients do not respond to the outcome questionnaire at one of time points, we report the reason of no availability of data in detail in our manuscript and discuss the potential bias. If no data of the outcome questionnaire is available, we will report the reasons in detail in our paper and discuss the potential bias. When one or more (but not all) of the measurements are missing, a sensitivity analysis will be performed to clarify if the missing data is at random.

Furthermore, we assess the progress of the QDASH, PRWHE, VAS, EQ5D-5L and physical examination during the 1 year follow-up. The random and fixed effects will remain similar, only all follow up moments will be included in the repeated measurements.

Cost-effectiveness analysis (CEA)

General considerations

We assess the cost-effectiveness of 3 days supportive bandage versus 14 days below-elbow cast over a period of 12 months. To answer this question, we perform a cost-utility analysis to estimate the incremental cost per QALY gained, to be able to compare our study with other studies in musculoskeletal disorders and in other disease categories. We impute missing data by multiple imputation, using the pattern mixture model. Using non-parametric bootstrapping (randomly drawing 5000 observations with replacement from the patient sample), the degree of uncertainty for costs and health effects and the cost-utility ratio are depicted in a cost-effectiveness plane. In addition, an acceptability curve is drawn, which indicates the probability that the intervention studied has lower incremental costs per QALY gained than various thresholds (dependent on disease severity) for the maximum willingness to pay for an extra QALY.

Cost analysis

The economic analysis is based on the societal perspective, and on the healthcare perspective in which the direct medical and productivity costs between the groups are compared. To measure the direct costs, an inventory of patients total medical consumption is made using the electronic patient

For peer review only - http://bmjopen.bmj.com/site/about/guidelines.xhtml

For peer review only

status and the iMCQ. The use of diagnostics (additional radiographs, CT, MRI, ultrasound or bone scintigraphy), consultations, emergency department visits, physical therapy, medication and aids are evaluated. Productivity costs are measured with the iPCQ. Costs of absence of work and decreased productivity at a paid job or at unpaid work are evaluated. The friction cost method is used to calculate the productivity costs according to the recent Dutch guidelines. The costs per unit of medical consumption is estimated, using information from the most recent Dutch Manual for economic evaluation of health care. Costs are reported for the year 2019.

Cost-utility analysis

Patient outcomes relevant for the CEA are the number of QALYs during 1 year. Patient scores on the EQ 5D-5 level version are converted into utility values, using the valuation algorithm of the Dutch general population.

Budget impact analysis (BIA)

Guidelines of the ISPOR Task Force are used for the BIA. Relevant features and tariffs of the Dutch health care system, anticipated uptake of the new intervention as well as usual care are taken into account. The size and the eligibility of the population, cost of diagnostics, treatment modalities and changes expected in condition-related costs are considered in the BIA. Sensitivity analyses are performed using divergent scenarios from the viewpoint of the decision-makers.

The budget impact per year of implementing the new intervention is estimated. All elements of medical costs for the new intervention as well as for usual care that are paid by third-party payers are considered and calculated.

ETHICS AND DISSEMINATION

For peer review only - http://bmjopen.bmj.com/site/about/guidelines.xhtml

Safety considerations

The study compares treatment of below-elbow cast (standard treatment) with supportive bandage (intervention). Since the study is labeled as low risk, a data safety monitoring board is not required. The study is monitored at least once a year and the progress of the trial is reported once a year to the accredited medical review ethics committee (MEC). Adverse events are recorded by the researcher during follow up. A serious adverse event is reported (www.toetsingonline.nl) within a maximum of 15 days after the researcher is notified.

Adverse events are defined as:

- Cast related problems
- Bandage related problems
- Loss of function
- Complex regional pain syndrome
- Persisting pain after 6 weeks

Serious adverse events related to our study are defined as:

- Surgically treated scaphoid fracture
- Compartment syndrome
- Scaphoid non-union (scaphoid fracture without signs of healing after minimally 12 weeks).

Because of the multicenter study design, clinical trial site agreements are obtained between the initiating and the participating hospitals. These agreements include liability and insurance aspects. The patient information sheet reports on participants insurance and potential complications as a result of participation in the study. Withdrawal from the study is always possible for included patients without any consequences.

For peer review only - http://bmjopen.bmj.com/site/about/guidelines.xhtml

Ethics

The study is approved by the MEC of the Erasmus Medical Centrum, Rotterdam, the Netherlands (MEC-2017-504). All amendments are notified to the MEC and changes to the study are made after a favorable opinion of the MEC.

The study is conducted according to the principles of the Declaration of Helsinki (64th version, date: October 2013) and in accordance with the Medical Research Involving Human Subjects Act (WMO) and other guidelines, regulations, and acts.

The study is registered in the Dutch Clinical trials Registry (www.trialregister.nl, trial NL6976).

Participating patients and physicians are not paid for participation in the study.

Data management

Personal data of the participating patients that we collect during the study, is changed to a study number. This study number is used for all study documentation, study reports and publications.

Data is collected with Castor EDC. Paper case report forms are entered in Castor EDC by the researcher. The paper case report forms are securely filed in the hospital. All data is stored for 15 years. The final trial dataset is accessible for the research team.

Dissemination

After completion of the study, we plan to present the results at (inter)national congresses and submit the manuscript to general peer-review journals.

Authorship is according to the International Committee of Medical Journal Editors guidelines and a SUSPECT study group is made.

For peer review only - http://bmjopen.bmj.com/site/about/guidelines.xhtml

REFERENCES

1. Inoue G, Sakuma M. The natural history of scaphoid non-union. Radiographical and clinical analysis in 102 cases. ARCH ORTHOP TRAUMA SURG. 1996;115(1):1-4.
2. Mallee WH, Veltman ES, Doornberg JN, Blankevoort L, van Dijk CN, Goslings JC. [Variations in management of suspected scaphoid fractures]
Variatie in beleid bij vermoeden op scafoidfractuur. Ned Tijdschr Geneeskd. 2012;156(28):A4514.
3. Borel C, Larbi A, Delclaux S, Lapegue F, Chiavassa-Gandois H, Sans N, et al. Diagnostic value of cone beam computed tomography (CBCT) in occult scaphoid and wrist fractures. Eur J Radiol. 2017;97:59-64.
4. Bergh TH, Steen K, Lindau T, Soldal LA, Bernardshaw SV, Lunde L, et al. Costs analysis and comparison of usefulness of acute MRI and 2 weeks of cast immobilization for clinically suspected scaphoid fractures. Acta Orthop. 2015;86(3):303-9.
5. de Zwart AD, Beeres FJ, Rhemrev SJ, Bartlema K, Schipper IB. Comparison of MRI, CT and bone scintigraphy for suspected scaphoid fractures. Eur J Trauma Emerg Surg. 2016;42(6):725-31.
6. Gemme S, Tubbs R. What physical examination findings and diagnostic imaging modalities are most useful in the diagnosis of scaphoid fractures? Ann Emerg Med. 2015;65(3):308-9.
7. DaCruz DJ, Bodiwala GG, Finlay DB. The suspected fracture of the scaphoid: a rational approach to diagnosis. Injury. 1988;19(3):149-52.
8. Sjølin SU, Andersen JC. Clinical fracture of the carpal scaphoid—supportive bandage or plaster cast immobilization? The Journal of Hand Surgery: British & European Volume. 1988;13(1):75-6.
9. Langhoff O, Andersen JL. Consequences of late immobilization of scaphoid fractures. J Hand Surg Br. 1988;13(1):77-9.
10. Leslie IJ, Dickson RA. The fractured carpal scaphoid. Natural history and factors influencing outcome. J Bone Joint Surg Br. 1981;63-B(2):225-30.
11. Schulz KF, Altman DG, Moher D. CONSORT 2010 Statement: updated guidelines for reporting parallel group randomised trials. Bmj. 2010;340:c332.
12. Chan AW, Tetzlaff JM, Gotzsche PC, Altman DG, Mann H, Berlin JA, et al. SPIRIT 2013 explanation and elaboration: guidance for protocols of clinical trials. Bmj. 2013;346:e7586.
13. Ciwit BV. Castor Electronic Data Capture. Amsterdam2016.
14. Mallee WH, Henny EP, van Dijk CN, Kamminga SP, van Enst WA, Kloen P. Clinical diagnostic evaluation for scaphoid fractures: a systematic review and meta-analysis. J Hand Surg Am. 2014;39(9):1683-91 e2.
15. Torok KS, Baker NA, Lucas M, Domsic RT, Boudreau R, Medsger TA, Jr. Reliability and validity of the delta finger-to-palm (FTP), a new measure of finger range of motion in systemic sclerosis. Clin Exp Rheumatol. 2010;28(2 Suppl 58):S28-36.
16. Kapandji A. [Clinical test of apposition and counter-apposition of the thumb]
Cotation clinique de l'opposition et de la contre-opposition du pouce. Ann Chir Main. 1986;5(1):67-73.
17. Skirven T. Clinical examination of the wrist. Journal of Hand Therapy. 1996;9(2):96-107.
18. Walsh JJ, Berger RA, Cooney WP. Current status of scapholunate interosseous ligament injuries. JAAOS-Journal of the American Academy of Orthopaedic Surgeons. 2002;10(1):32-42.
19. Tsang P, Walton D, Grewal R, MacDermid J. Validation of the QuickDASH and DASH in Patients With Distal Radius Fractures Through Agreement Analysis. Arch Phys Med Rehabil. 2017;98(6):1217-22 e1.
20. Kleinlugtenbelt YV, Krol RG, Bhandari M, Goslings JC, Poolman RW, Scholtes VAB. Are the patient-rated wrist evaluation (PRWE) and the disabilities of the arm, shoulder and hand (DASH) questionnaire used in distal radial fractures truly valid and reliable? Bone Joint Res. 2018;7(1):36-45.
21. Badalamente M, Coffelt L, Elfar J, Gaston G, Hammert W, Huang J, et al. Measurement scales in clinical research of the upper extremity, part 1: general principles, measures of general health, pain, and patient satisfaction. J Hand Surg Am. 2013;38(2):401-6; quiz 6.

For peer review only - http://bmjopen.bmj.com/site/about/guidelines.xhtml

22. Hung M-C, Lu W-S, Chen S-S, Hou W-H, Hsieh C-L, Wang J-D. Validation of the EQ-5D in Patients with Traumatic Limb Injury. Journal of Occupational Rehabilitation. 2015;25(2):387-93.

23. Bouwmans C, Krol M, Severens H, Koopmanschap M, Brouwer W, Hakkaart-van Roijen L. The iMTA Productivity Cost Questionnaire: A Standardized Instrument for Measuring and Valuing Health-Related Productivity Losses. Value Health. 2015;18(6):753-8.

24. Franchignoni F, Vercelli S, Giordano A, Sartorio F, Bravini E, Ferriero G. Minimal clinically important difference of the disabilities of the arm, shoulder and hand outcome measure (DASH) and its shortened version (QuickDASH). J Orthop Sports Phys Ther. 2014;44(1):30-9.

25. Buijze GA, Goslings JC, Rhemrev SJ, Weening AA, Van Dijkman B, Doornberg JN, et al. Cast immobilization with and without immobilization of the thumb for nondisplaced and minimally displaced scaphoid waist fractures: a multicenter, randomized, controlled trial. J Hand Surg Am. 2014;39(4):621-7.

Author's contributions:

MR and JC conceived the study idea and secured funding. AC, MR, GK, NM, MK, JV, SM and JC were involved with the initial study design. MR and MK were involved with the statistical analysis for the study. AC, GK, SM, JC, SUSPECT study group are involved with the implementation of the protocol and the data collection. AC is conducting the study. All authors provided critical comments to the manuscript and approved the final version before submitting.

Funding

For this study, we received a grant from ZonMw, a Dutch organization for health research and care innovation and co-funding from CZ, a Dutch health insurance company. Both organizations were not involved in the study design.

Trial status

We are currently recruiting patients for the study. The date of first enrollment was 7 june 2018.

Competing interests statement

The authors declare to have no competing interests.

For peer review only

For peer review only - http://bmjopen.bmj.com/site/about/guidelines.xhtml

562 **Legend:**

563 **Figure 1** SUSPECT study flow chart. PROMs; Patient rated outcome measures

564 **Figure 2** Treatment of intervention and control group

565

566

567

568

569

For peer review only

For peer review only - http://bmjopen.bmj.com/site/about/guidelines.xhtml

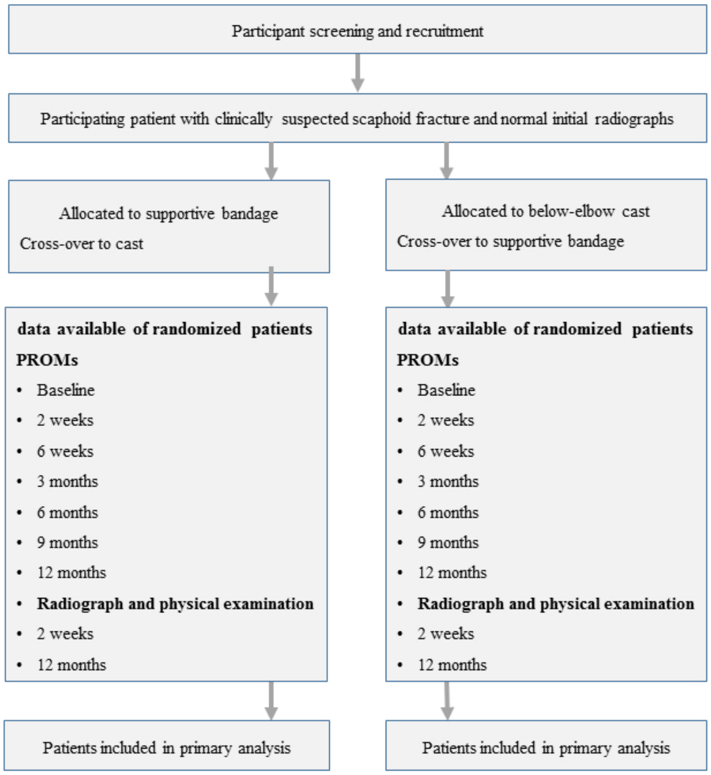

Figure 1 SUSPECT study flow chart. PROMs; Patient rated outcome measures

190x275mm (96 x 96 DPI)

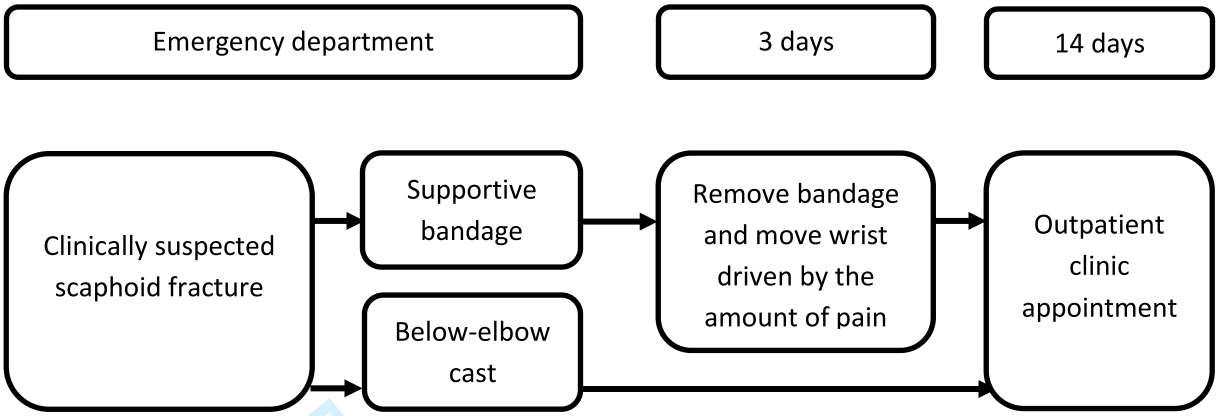

For peer review only

Polspijn zonder afwijkingen op de röntgenfoto's

Proefpersoneninformatie voor deelname aan medisch-wetenschappelijk onderzoek

Titel van het onderzoek

Polspijn zonder afwijkingen op de röntgenfoto's

Geachte heer/mevrouw,

U heeft de spoedeisende hulp bezocht vanwege pijn in uw pols. Wij willen u vragen of u wilt deelnemen aan een medisch-wetenschappelijk onderzoek. U beslist zelf of u wilt meedoen. Voordat u de beslissing neemt, is het belangrijk om meer te weten over het onderzoek. Lees daarom deze informatiebrief rustig door. Bespreek het met partner, vrienden of familie. Ook is er een onafhankelijke persoon, die veel weet van het onderzoek. Lees ook de Algemene brochure. Daar staat veel algemene informatie over medisch-wetenschappelijk onderzoek in. Heeft u na het lezen van de informatie nog vragen? Dan kunt u terecht bij de arts-onderzoeker. In bijlage C vindt u haar contactgegevens.

1. Algemene informatie

Dit onderzoek is opgezet door het Erasmus MC en het Reinier de Graaf Gasthuis en wordt gedaan door artsen in verschillende ziekenhuizen.

Voor dit onderzoek zijn 180 proefpersonen nodig die polspijn hebben na een ongeval.

De medisch-ethische toetsingscommissie van het Erasmus MC heeft dit onderzoek goedgekeurd.

Algemene informatie over de toetsing van onderzoek vindt u in de brochure 'Medisch-wetenschappelijk onderzoek'. Deze brochure is ook online beschikbaar. Zie hiervoor de volgende link: file:///H:/Downloads/medisch-wetenschappelijk+onderzoek+algemene+informatie+voor+de+proefpersoon.pdf

2. Wat is het doel van het onderzoek?

Polspijn na een ongeval kan veroorzaakt worden door een kneuzing of een botbreuk. Om het onderscheid te kunnen maken tussen een kneuzing of een botbreuk zijn er bij u een aantal röntgenfoto's gemaakt. Er zijn op dit moment op uw röntgenfoto's geen botbreuken te zien zijn, maar een breuk van het scheepvormige botje kan soms pas te zien zijn na 2 weken. Daarom worden alle patiënten met verdenking op een botbreuk van het scheepvormige botje behandeld met onderarmgips en na 2 weken teruggezien. Aangezien uiteindelijk maar 1 van de 10 patiënten een

For peer review only - http://bmjopen.bmj.com/site/about/guidelines.xhtml

botbreuk heeft worden dus veel patiënten onnodig behandeld met gips. Met dit onderzoek willen we daarom kijken of de behandeling niet even goed is met een drukverband gedurende 3 dagen in plaats van een onderarmgips voor 2 weken.

3. Wat wordt er onderzocht?

In dit onderzoek wordt een drukverband vergeleken met een onderarm gips. Om dit te kunnen beoordelen willen wij u vragen om gedurende de studie een aantal vragenlijsten in te vullen. Voor het onderzoek is het belangrijk dat u dit zo objectief mogelijk invult. Daarnaast zullen wij de functie van de pols beoordelen.

4. Wat meedoen inhoudt?

Als u meedoet, duurt dat totaal ongeveer 1 jaar voor u. De studie gaat in totaal vier jaar duren.

Behandeling

Als u besluit om mee te doen zal er worden geloot tussen de behandeling met onderarmgips gedurende 2 weken en de behandeling met drukverband gedurende 3 dagen. Uw behandelend arts en de onderzoekers hebben geen invloed op de uitslag van de loting.

Bezoek en metingen

Gedurende de studie zullen wij op vijf momenten vragen om vragenlijsten in te vullen (ongeveer 15 minuten per meetmoment). Via deze vragenlijsten zullen wij het dagelijks functioneren van de arm, kwaliteit van leven en de kosten met betrekking tot medische zorg en arbeidsverzuim evalueren. Daarnaast zullen wij tijdens twee bezoeken aan het ziekenhuis de onderarmen onderzoeken op bewegelijkheid en kracht. Ook zullen er röntgenfoto's worden gemaakt om de botten van de pols goed in beeld te brengen. Op het moment dat er tijdens deze controles een breuk wordt ontdekt op de röntgenfoto's zal de behandeling zich richten op herstel van deze breuk. In bijlage B vindt u een overzicht van alles wat wordt gepland als u deelneemt.

Anders dan gebruikelijke zorg

Als u aan het onderzoek deelneemt dan krijgt u afhankelijk van de loting een drukverband. Dit is momenteel niet de standaardbehandeling. Indien u inloot voor de groep met het onderarmgips, dan krijgt u gewoon de behandeling die u ook zou krijgen als u niet meedeed aan dit onderzoek.
Na twee weken wordt er nogmaals een röntgenfoto gemaakt. Dit maakt onderdeel uit van de standaardbehandeling en zal dus ook gebeuren als u niet meedoet aan dit onderzoek. Mocht er dan toch een botbreuk op de röntgenfoto's te zien zijn, wordt u alsnog behandeld voor de botbreuk. Eerder onderzoek heeft aangetoond dat latere behandeling van een botbreuk van het scheepvormige botje geen nadelige gevolgen heeft voor het uiteindelijk herstel. Indien u deelneemt aan het onderzoek krijgt u een extra poliklinische controle na 1 jaar. Deze extra poliklinische controle

For peer review only - http://bmjopen.bmj.com/site/about/guidelines.xhtml

For peer review only

is voor beide groepen hetzelfde. Hier zullen voor u geen extra kosten aan verbonden zijn. De reguliere behandeling bestaat uit een onderarmgips en poliklinische controle met röntgenfoto's twee weken na het ongeval (zie bijlage B).

5. Wat wordt er van u verwacht?

Om het onderzoek goed te laten verlopen is het belangrijk dat u zich aan de volgende afspraken houdt.

De afspraken zijn dat u:

- de gips of verband behandeling volgt volgens de uitleg.
- niet ook nog aan een ander medisch-wetenschappelijk onderzoek meedoet, die invloed heeft op de huidige studie.
- afspraken voor bezoeken nakomt.

Het is belangrijk dat u contact opneemt met de onderzoeker:

- als u in een ziekenhuis wordt opgenomen of behandeld.
- als u plotseling gezondheidsklachten krijgt.
- als u niet meer wilt meedoen aan het onderzoek.
- als uw contactgegevens wijzigen.

6. Welke bijwerkingen en risico's kunt u verwachten?

De behandeling van polspijn zonder afwijkingen op de röntgenfoto's brengt niet veel bijwerkingen of risico's met zich mee. De behandeling met gips zal meer kans geven op drukplekken van het gips en stijfheid van de pols. De behandeling met drukverband geeft mogelijk een groter risico op trager herstel van een mogelijk botbreuk van het scheepvormig botje alhoewel eerdere studies dit ontkrachten.

7. Wat zijn mogelijke voor- en nadelen van deelname aan dit onderzoek?

U heeft zelf geen voordeel van deelname aan dit onderzoek. Voor de toekomst kan het onderzoek wel nuttige gegevens opleveren. Een nadeel van deelname aan de studie is dat wij extra tijd van u vragen voor het invullen van de vragenlijsten en de extra metingen.

8. Wat gebeurt er als u niet wenst deel te nemen aan dit onderzoek?

U beslist zelf of u meedoet aan het onderzoek. Deelname is vrijwillig. Als u besluit niet mee te doen, hoeft u verder niets te doen. U hoeft niets te tekenen. U hoeft ook niet te zeggen waarom u niet wilt meedoen. U krijgt natuurlijk alle noodzakelijke medische behandelingen. Als u wel meedoet, kunt u zich altijd bedenken en toch nog stoppen. Dat kan ook tijdens het onderzoek. Wel moet u dit direct

For peer review only - http://bmjopen.bmj.com/site/about/guidelines.xhtml

melden aan de onderzoeker. Wij vragen u dan wel of we enkele gegevens uit uw patiëntendossier mogen gebruiken voor het onderzoek.

De gegevens die tot dat moment zijn verzameld, worden gebruikt voor het onderzoek.

Als er nieuwe informatie over het onderzoek is die belangrijk voor u is, laat de onderzoeker dit aan u weten. U wordt dan gevraagd of u blijft meedoen.

9. Wat gebeurt er als het onderzoek is afgelopen?

Uw deelname aan het onderzoek stopt als

- alle bezoeken [volgens het schema/zoals beschreven onder punt 4] voorbij zijn
- u zelf kiest om te stoppen
- de onderzoeker het beter voor u vindt om te stoppen
- de subsidiegever van het onderzoek, de overheid of de beoordelende medisch-ethische toetsingscommissie, besluit om het onderzoek te stoppen.

Het hele onderzoek is afgelopen als alle deelnemers klaar zijn.

Na het verwerken van alle gegevens informeert de onderzoeker u over de belangrijkste uitkomsten van het onderzoek.

10. Gebruik en bewaren van uw gegevens

Voor dit onderzoek is het nodig dat uw medische gegevens worden verzameld en gebruikt. Elke proefpersoon krijgt een code die op de gegevens komt te staan. Uw naam en andere persoonlijke gegevens die u direct kunnen identificeren worden daarbij weggelaten

Uw gegevens

Al uw gegevens blijven vertrouwelijk.

Sommige mensen mogen uw medische en persoonlijke gegevens inzien. Dit is om te controleren of het onderzoek goed en betrouwbaar uitgevoerd is. Algemene informatie hierover vindt u in de brochure 'Medisch-wetenschappelijk onderzoek'.

Mensen die uw gegevens kunnen inzien zijn: het onderzoeksteam, een controleur die voor de opdrachtgever van het onderzoek werkt, de Inspectie voor de Gezondheidszorg. Zij houden uw gegevens geheim. De onderzoeker van het coördinerend centrum (Erasmus MC) ontvangt uw gegevens voor het versturen van de vragenlijsten. Deze gegevens zullen na het einde van het onderzoek vernietigd worden.

Als u de toestemmingsverklaring ondertekent, geeft u toestemming voor het verzamelen, bewaren en inzien van uw medische en persoonlijke gegevens.

De onderzoeker bewaart uw gegevens 15 jaar.

Later gebruik gegevens

For peer review only - http://bmjopen.bmj.com/site/about/guidelines.xhtml

Polspijn zonder afwijkingen op de röntgenfoto's

Wij willen uw gegevens graag bewaren. Misschien kunnen we daar later extra onderzoek mee doen. Op het toestemmingsformulier kunt u aangeven of u hiermee akkoord gaat. U kunt deze toestemming altijd weer intrekken.

Inzien gegevens en intrekken toestemming

U heeft te allen tijde het recht om de over u verzamelde gegevens in te zien, te wijzigen of uw toestemming voor gebruik van uw persoonsgegevens weer in te trekken. De onderzoeksgegevens die zijn verzameld tot het moment dat u uw toestemming intrekt worden nog wel gebruikt in het onderzoek.

Meer informatie over uw rechten bij verwerking van gegevens

Voor algemene informatie over uw rechten bij verwerking van uw persoonsgegevens kunt u de website van de Autoriteit Persoonsgegevens raadplegen (https://autoriteitpersoonsgegevens.nl).

Bij vragen over uw rechten kunt u contact opnemen met de verantwoordelijke voor de verwerking van uw persoonsgegevens. Voor dit onderzoek is dat:

Erasmus Medisch Centrum: Zie bijlage C voor contactgegevens.

Bij vragen of klachten over de verwerking van uw persoonsgegevens raden we u aan eerst contact op te nemen met de onderzoekslocatie. U kunt ook contact opnemen met de Functionaris voor de Gegevensbescherming van de instelling [contactgegevens in bijlage C] of de Autoriteit Persoonsgegevens.

11. Verzekering voor proefpersonen

Voor iedereen die meedoet aan dit onderzoek is een verzekering afgesloten. De verzekering dekt schade als gevolg van het onderzoek. Dit geldt voor schade die naar boven komt gedurende het onderzoek. In bijlage A vindt u de verzekerde bedragen, de uitzonderingen en de adresgegevens van de verzekeraar.

12. Informeren huisarts

Uw huisarts wordt geïnformeerd over uw deelname aan het onderzoek. Dit is voor uw eigen veiligheid. Als u dit niet goed vindt, kunt u niet meedoen aan dit onderzoek. U kunt niet deelnemen aan het onderzoek als u geen huisarts heeft.

13. Zijn er extra kosten/is er een vergoeding wanneer u besluit aan dit onderzoek mee te doen?

Er zullen geen extra kosten in rekening worden gebracht bij deelname aan het onderzoek. Het bezoek aan de spoedeisende hulp is standaard verzekerde zorg. Het extra poliklinische bezoek na 1

For peer review only - http://bmjopen.bmj.com/site/about/guidelines.xhtml

jaar en de daarbij behorende röntgenfoto's zullen betaald worden vanuit onderzoeksgeld en dus niet aan u worden doorberekend.

14. Wilt u verder nog iets weten?

Wilt u graag een onafhankelijk advies over meedoen aan dit onderzoek? Dan kunt u terecht bij een onafhankelijke arts. Zijn gegevens vindt u in *lokale informatie onafhankelijk arts* (bijlage C).
Indien u een klacht heeft, dan kunt u deze het beste eerst bespreken met degene die er rechtstreeks bij betrokken is. Kunt u uw klacht of ongenoegen, om welke reden dan ook, niet rechtstreeks bespreken of bent u na het bespreken van uw klacht niet tevreden, dan helpt de klachtenfunctionaris u graag verder. De gegevens van de klachtenfunctionaris vindt u in bijlage C.

15. Ondertekening toestemmingsformulier

Indien u toestemming geeft over deelname aan dit onderzoek, zullen wij u vragen deze op de bijbehorende toestemmingsverklaring schriftelijk te bevestigen. Door uw schriftelijke toestemming geeft u aan dat u de informatie heeft begrepen en instemt met deelname aan het onderzoek.
Het handtekeningenblad wordt door de onderzoeker bewaard. U krijgt een kopie of een tweede exemplaar van deze toestemmingsverklaring.

Dank voor uw aandacht.

Orthopedisch chirurgen Erasmus MC en het onderzoeksteam

For peer review only

For peer review only - http://bmjopen.bmj.com/site/about/guidelines.xhtml

Polspijn zonder afwijkingen op de röntgenfoto's

Bijlagen

A. Informatie over de verzekering
B. Overzicht metingen onderzoek
C. Lokale informatie
D. Toestemmingsverklaring

For peer review only

For peer review only - http://bmjopen.bmj.com/site/about/guidelines.xhtml

Polspijn zonder afwijkingen op de röntgenfoto's

Bijlage A: Informatie over de verzekering

Voor iedereen die meedoet aan dit onderzoek is een verzekering afgesloten. De verzekering dekt schade die het gevolg is van deelname aan het onderzoek. Dit geldt voor schade die naar boven komt tijdens het onderzoek, of binnen vier jaar na het einde van het onderzoek. U moet de schade ook binnen die 4 jaar aan de verzekeraar hebben gemeld.

In geval van schade kunt u zich direct wenden tot de verzekeraar.

De verzekeraar van het onderzoek is:

CAN Insurance Company Limited,

Mevrouw Esther van Herk

+31(0)205737274

Esther.vanherk@cnahardy.com

De verzekering biedt een maximum dekking van € 650.000 per proefpersoon, met een maximumbedrag van € 5.000.000 voor het gehele onderzoek. Indien de opdrachtgever van dit onderzoek meerdere onderzoeken heeft lopen, geldt een maximumbedrag van € 7.500.000 euro per verzekeringsjaar voor álle onderzoeken. De dekking van specifieke schades en kosten is verder tot bepaalde bedragen beperkt. Deze vindt u in het *Besluit verplichte verzekering bij medisch wetenschappelijk onderzoek met mensen*. Informatie hierover kunt u vinden op de website van de Centrale Commissie Mensgebonden Onderzoek: www.ccmo.nl.

Voor deze verzekering gelden voorts een aantal uitsluitingen. De verzekering dekt niet:

- schade waarvan op grond van de aard van het onderzoek (nagenoeg) zeker was dat deze zich zou voordoen;
- schade aan de gezondheid die ook zou zijn ontstaan als u niet aan het onderzoek had deelgenomen;
- schade die het gevolg is van het (niet) volledig nakomen van aanwijzingen of instructies;
- schade aan nakomelingen, als gevolg van een nadelige inwerking van het onderzoek op u of uw nakomeling;
- bij onderzoek naar bestaande behandelmethoden: schade die het gevolg is van één van deze behandelmethoden;
- bij onderzoek naar de behandeling van specifieke gezondheidsproblemen: schade die het gevolg is van uitblijvende verbetering of van verslechtering van deze gezondheidsproblemen.

For peer review only - http://bmjopen.bmj.com/site/about/guidelines.xhtml

Polspijn zonder afwijkingen op de röntgenfoto's

	**T1** Start studie	**T 2** 2 weken na start studie	**T3** 6 weken na start studie	**T4** 3 maanden na start studie	**T5** 6 maanden na start studie	**T6** 9 maanden na start studie	**T7**12 maanden na start studie
Vragenlijsten (thuis)	+	+	+	+	+	+	+
Telefonisch contact					+	+	
Lichamelijk onderzoek (polikliniek)	*	*					+
Röntgenfoto's (polikliniek)	*	*					+

Bijlage B: overzicht metingen onderzoek

+ = extra metingen voor onderzoek, wordt uitgevoerd / afgenomen bij deze controle
* = metingen die behoren bij gebruikelijke zorg, wordt uitgevoerd bij dit bezoek

For peer review only - http://bmjopen.bmj.com/site/about/guidelines.xhtml

Polspijn zonder afwijkingen op de röntgenfoto's

Bijlage C. Lokale informatie Erasmus Medisch Centrum

Hoofdonderzoeker:

dr. J.W. Colaris 010-7034686

orthopedisch Chirurg

(via secretariaat Orthopedie)

Onafhankelijk arts:

dr. D.E. Meuffels, 010-7034686

orthopaedisch chirurg Erasmus MC

(via secretariaat Orthopaedie)

Arts-onderzoeker:

Abigaël Cohen 06-24788338

suspect@erasmusmc.nl

Klachtencommissie:

Klachtenfunctionarissen Erasmus MC 010-7033198

Functionaris voor de Gegevensbescherming van de instelling:

Voor meer informatie over uw rechten: 010-7034986

(via secretariaat afdeling Juridische Zaken)

Autoriteit Persoonsgegevens https://autoriteitpersoonsgegevens.nl

For peer review only - http://bmjopen.bmj.com/site/about/guidelines.xhtml

Bijlage D: Toestemmingsformulier proefpersoon

Titel van het onderzoek: Polspijn zonder afwijkingen op de röntgenfoto's

- Ik heb de informatiebrief voor de proefpersoon gelezen. Ik kon aanvullende vragen stellen. Mijn vragen zijn genoeg beantwoord. Ik had genoeg tijd om te beslissen of ik meedoe.
- Ik weet dat meedoen helemaal vrijwillig is. Ik weet dat ik op ieder moment kan beslissen om toch niet mee te doen. Daarvoor hoef ik geen reden te geven.
- Ik geef toestemming om mijn huisarts te vertellen dat ik meedoe aan dit onderzoek.
- Ik weet dat sommige mensen mijn gegevens kunnen zien. Die mensen staan vermeld in deze informatiebrief.
- Ik geef toestemming om mijn gegevens te gebruiken, voor de doelen die in de informatiebrief staan.
- Ik geef toestemming om mijn onderzoeksgegevens 15 jaar na afloop van dit onderzoek te bewaren.
- Ik geef toestemming om in de toekomst opnieuw benaderd te worden voor vervolg onderzoek. Indien ik dit niet wens, zal ik dat kenbaar maken.
- Ik wil meedoen aan dit onderzoek.

Naam proefpersoon: Handtekening:

Datum : __ / __ / __

Ik verklaar hierbij dat ik deze proefpersoon volledig heb geïnformeerd over het genoemde onderzoek.

Als er tijdens het onderzoek informatie bekend wordt die de toestemming van de proefpersoon zou kunnen beïnvloeden, dan breng ik hem/haar daarvan tijdig op de hoogte.

Naam onderzoeker (of diens vertegenwoordiger):

Handtekening: Datum: __ / __ / __

* Doorhalen wat niet van toepassing is.

For peer review only - http://bmjopen.bmj.com/site/about/guidelines.xhtml

STANDARD PROTOCOL ITEMS: RECOMMENDATIONS FOR INTERVENTIONAL TRIALS

SPIRIT 2013 Checklist: Recommended items to address in a clinical trial protocol and related documents*

Section/item	ItemNo	Description	Page
**Administrative information**			
Title	1	Descriptive title identifying the study design, population, interventions, and, if applicable, trial acronym	1
Trial registration	2a	Trial identifier and registry name. If not yet registered, name of intended registry	3
	2b	All items from the World Health Organization Trial Registration Data Set	n/a
Protocol version	3	Date and version identifier	footer
Funding	4	Sources and types of financial, material, and other support	3
Roles and responsibilities	5a	Names, affiliations, and roles of protocol contributors	1-3
	5b	Name and contact information for the trial sponsor	2
	5c	Role of study sponsor and funders, if any, in study design; collection, management, analysis, and interpretation of data; writing of the report; and the decision to submit the report for publication, including whether they will have ultimate authority over any of these activities	3
	5d	Composition, roles, and responsibilities of the coordinating centre, steering committee, endpoint adjudication committee, data management team, and other individuals or groups overseeing the trial, if applicable (see Item 21a for data monitoring committee)	n/a

For peer review only - http://bmjopen.bmj.com/site/about/guidelines.xhtml

			Page
**Introduction**			
Background and rationale	6a	Description of research question and justification for undertaking the trial, including summary of relevant studies (published and unpublished) examining benefits and harms for each intervention	5-6
	6b	Explanation for choice of comparators	5-6
Objectives	7	Specific objectives or hypotheses	6
Trial design	8	Description of trial design including type of trial (eg, parallel group, crossover, factorial, single group), allocation ratio, and framework (eg, superiority, equivalence, noninferiority, exploratory)	6
**Methods: Participants, interventions, and outcomes**			
Study setting	9	Description of study settings (eg, community clinic, academic hospital) and list of countries where data will be collected. Reference to where list of study sites can be obtained	7
Eligibility criteria	10	Inclusion and exclusion criteria for participants. If applicable, eligibility criteria for study centres and individuals who will perform the interventions (eg, surgeons, psychotherapists)	8
Interventions	11a	Interventions for each group with sufficient detail to allow replication, including how and when they will be administered	9
	11b	Criteria for discontinuing or modifying allocated interventions for a given trial participant (eg, drug dose change in response to harms, participant request, or improving/worsening disease)	9
	11c	Strategies to improve adherence to intervention protocols, and any procedures for monitoring adherence (eg, drug tablet return, laboratory tests)	9
	11d	Relevant concomitant care and interventions that are permitted or prohibited during the trial	9

For peer review only - http://bmjopen.bmj.com/site/about/guidelines.xhtml

			Page
Outcomes	12	Primary, secondary, and other outcomes, including the specific measurement variable (eg, systolic blood pressure), analysis metric (eg, change from baseline, final value, time to event), method of aggregation (eg, median, proportion), and time point for each outcome. Explanation of the clinical relevance of chosen efficacy and harm outcomes is strongly recommended	9-13
Participant timeline	13	Time schedule of enrolment, interventions (including any run-ins and washouts), assessments, and visits for participants. A schematic diagram is highly recommended (see Figure)	Figure 1<br>Table 1<br>Table 2
Sample size	14	Estimated number of participants needed to achieve study objectives and how it was determined, including clinical and statistical assumptions supporting any sample size calculations	14-15
Recruitment	15	Strategies for achieving adequate participant enrolment to reach target sample size	15
**Methods: Assignment of interventions (for controlled trials)**			
Allocation:			
Sequence generation	16a	Method of generating the allocation sequence (eg, computer-generated random numbers), and list of any factors for stratification. To reduce predictability of a random sequence, details of any planned restriction (eg, blocking) should be provided in a separate document that is unavailable to those who enrol participants or assign interventions	9
Allocation concealment mechanism	16b	Mechanism of implementing the allocation sequence (eg, central telephone; sequentially numbered, opaque, sealed envelopes), describing any steps to conceal the sequence until interventions are assigned	9
Implementation	16c	Who will generate the allocation sequence, who will enrol participants, and who will assign participants to interventions	7
Blinding (masking)	17a	Who will be blinded after assignment to interventions (eg, trial participants, care providers, outcome assessors, data analysts), and how	9

For peer review only - http://bmjopen.bmj.com/site/about/guidelines.xhtml

	17b	If blinded, circumstances under which unblinding is permissible, and procedure for revealing a participant's allocated intervention during the trial	n/a
**Methods: Data collection, management, and analysis**			
Data collection methods	18a	Plans for assessment and collection of outcome, baseline, and other trial data, including any related processes to promote data quality (eg, duplicate measurements, training of assessors) and a description of study instruments (eg, questionnaires, laboratory tests) along with their reliability and validity, if known. Reference to where data collection forms can be found, if not in the protocol	9-14
	18b	Plans to promote participant retention and complete follow-up, including list of any outcome data to be collected for participants who discontinue or deviate from intervention protocols	15-16
Data management	19	Plans for data entry, coding, security, and storage, including any related processes to promote data quality (eg, double data entry; range checks for data values). Reference to where details of data management procedures can be found, if not in the protocol	19
Statistical methods	20a	Statistical methods for analysing primary and secondary outcomes. Reference to where other details of the statistical analysis plan can be found, if not in the protocol	15-17
	20b	Methods for any additional analyses (eg, subgroup and adjusted analyses)	15-17
	20c	Definition of analysis population relating to protocol non-adherence (eg, as randomised analysis), and any statistical methods to handle missing data (eg, multiple imputation)	16
**Methods: Monitoring**			
Data monitoring	21a	Composition of data monitoring committee (DMC); summary of its role and reporting structure; statement of whether it is independent from the sponsor and competing interests; and reference to where further details about its charter can be found, if not in the protocol. Alternatively, an explanation of why a DMC is not needed	18

For peer review only - http://bmjopen.bmj.com/site/about/guidelines.xhtml

			Page
	21b	Description of any interim analyses and stopping guidelines, including who will have access to these interim results and make the final decision to terminate the trial	n/a
Harms	22	Plans for collecting, assessing, reporting, and managing solicited and spontaneously reported adverse events and other unintended effects of trial interventions or trial conduct	18
Auditing	23	Frequency and procedures for auditing trial conduct, if any, and whether the process will be independent from investigators and the sponsor	18
**Ethics and dissemination**			
Research ethics approval	24	Plans for seeking research ethics committee/institutional review board (REC/IRB) approval	19
Protocol amendments	25	Plans for communicating important protocol modifications (eg, changes to eligibility criteria, outcomes, analyses) to relevant parties (eg, investigators, REC/IRBs, trial participants, trial registries, journals, regulators)	19
Consent or assent	26a	Who will obtain informed consent or assent from potential trial participants or authorised surrogates, and how (see Item 32)	7
	26b	Additional consent provisions for collection and use of participant data and biological specimens in ancillary studies, if applicable	n/a
Confidentiality	27	How personal information about potential and enrolled participants will be collected, shared, and maintained in order to protect confidentiality before, during, and after the trial	19
Declaration of interests	28	Financial and other competing interests for principal investigators for the overall trial and each study site	21
Access to data	29	Statement of who will have access to the final trial dataset, and disclosure of contractual agreements that limit such access for investigators	19
Ancillary and post-trial care	30	Provisions, if any, for ancillary and post-trial care, and for compensation to those who suffer harm from trial participation	18

For peer review only - http://bmjopen.bmj.com/site/about/guidelines.xhtml

			Page
Dissemination policy	31a	Plans for investigators and sponsor to communicate trial results to participants, healthcare professionals, the public, and other relevant groups (eg, via publication, reporting in results databases, or other data sharing arrangements), including any publication restrictions	19
	31b	Authorship eligibility guidelines and any intended use of professional writers	21
	31c	Plans, if any, for granting public access to the full protocol, participant-level dataset, and statistical code	n/a
**Appendices**			
Informed consent materials	32	Model consent form and other related documentation given to participants and authorised surrogates	
Biological specimens	33	Plans for collection, laboratory evaluation, and storage of biological specimens for genetic or molecular analysis in the current trial and for future use in ancillary studies, if applicable	n/a

*It is strongly recommended that this checklist be read in conjunction with the SPIRIT 2013 Explanation & Elaboration for important clarification on the items. Amendments to the protocol should be tracked and dated. The SPIRIT checklist is copyrighted by the SPIRIT Group under the Creative Commons "Attribution-NonCommercial-NoDerivs 3.0 Unported" license.

For peer review only - http://bmjopen.bmj.com/site/about/guidelines.xhtml
